# Examining the Use of Glucose and Physical Activity Self-Monitoring Technologies in Individuals at Moderate to High Risk of Developing Type 2 Diabetes: Randomized Trial

**DOI:** 10.2196/14195

**Published:** 2019-10-28

**Authors:** Maxine E Whelan, Mark W Orme, Andrew P Kingsnorth, Lauren B Sherar, Francesca L Denton, Dale W Esliger

**Affiliations:** 1 Nuffield Department of Primary Care Health Sciences University of Oxford Oxford United Kingdom; 2 National Centre for Sport and Exercise Medicine Loughborough University Loughborough United Kingdom; 3 School of Sport, Exercise and Health Sciences Loughborough University Loughborough United Kingdom; 4 Department of Respiratory Sciences University of Leicester Leicester United Kingdom; 5 Centre for Exercise and Rehabilitation Science National Institute for Health Research Leicester Biomedical Research Centre-Respiratory Leicester United Kingdom; 6 National Institute for Health Research Leicester Biomedical Research Centre-Lifestyle Leicester United Kingdom

**Keywords:** usage, self-monitoring, feedback, behavior, physiology, wearable electronic devices, biobehavioral sciences

## Abstract

**Background:**

Self-monitoring of behavior (namely, diet and physical activity) and physiology (namely, glucose) has been shown to be effective in type 2 diabetes (T2D) and prediabetes prevention. By combining self-monitoring technologies, the acute physiological consequences of behaviors could be shown, prompting greater consideration to physical activity levels today, which impact the risk of developing diabetes years or decades later. However, until recently, commercially available technologies have not been able to show individuals the health benefits of being physically active.

**Objective:**

The objective of this study was to examine the usage, feasibility, and acceptability of behavioral and physiological self-monitoring technologies in individuals at risk of developing T2D.

**Methods:**

A total of 45 adults aged ≥40 years and at moderate to high risk of T2D were recruited to take part in a 3-arm feasibility trial. Each participant was provided with a behavioral (Fitbit Charge 2) and physiological (FreeStyle Libre flash glucose monitor) monitor for 6 weeks, masked according to group allocation. Participants were allocated to glucose feedback (4 weeks) followed by glucose and physical activity (biobehavioral) feedback (2 weeks; group 1), physical activity feedback (4 weeks) followed by biobehavioral feedback (2 weeks; group 2), or biobehavioral feedback (6 weeks; group 3). Participant usage (including time spent on the apps and number of glucose scans) was the primary outcome. Secondary outcomes were the feasibility (including recruitment and number of sensor displacements) and acceptability (including monitor wear time) of the intervention. Semistructured qualitative interviews were conducted at the 6-week follow-up appointment.

**Results:**

For usage, time spent on the Fitbit and FreeStyle Libre apps declined over the 6 weeks for all groups. Of the FreeStyle Libre sensor scans conducted by participants, 17% (1798/10,582) recorded rising or falling trends in glucose, and 24% (13/45) of participants changed ≥1 of the physical activity goals. For feasibility, 49% (22/45) of participants completed the study using the minimum number of FreeStyle Libre sensors, and a total of 41 sensors were declared faulty or displaced. For acceptability, participants wore the Fitbit for 40.1 (SD 3.2) days, and 20% (9/45) of participants and 53% (24/45) of participants were prompted by email to charge or sync the Fitbit, respectively. Interviews unearthed participant perceptions on the study design by suggesting refinements to the eligibility criteria and highlighting important issues about the usability, wearability, and features of the technologies.

**Conclusions:**

Individuals at risk of developing T2D engaged with wearable digital health technologies providing behavioral and physiological feedback. Modifications are required to both the study and to commercially available technologies to maximize the chances of sustained usage and behavior change. The study and intervention were feasible to conduct and acceptable to most participants.

**Trial Registration:**

International Standard Randomized Controlled Trial Number (ISRCTN) 17545949; isrctn.com/ISRCTN17545949

## Introduction

The prevalence of diabetes was estimated to be more than 3 million in England in 2017 [1]. It is a fast-growing health crisis in the United Kingdom and globally [2], but given that 3 out of 5 cases of type 2 diabetes (T2D) are preventable [3], efforts to prevent the onset of T2D in people identified at increased risk are a clear public health priority.

The British Medical Association reported in 2018 the need to prioritize prevention over cure for long-term conditions to secure the long-term sustainability of the National Health Service (NHS). In parallel, the NHS Long Term Plan outlined the need to promote digitally enabled care and patient empowerment around accessing digital tools [4]. NHS Digital is a key driving force attempting to harness the power of information and technology into existing health care pathways, using technologies such as wearables and Web-based platforms. In combination, the ability to self-monitor behavior and health using wearables has created new opportunities for people to actively participate in their health care in nonclinical settings [5,6].

Flash glucose monitoring (FGM), brought onto the market in 2016, allows users to monitor their interstitial glucose fluctuations. Early studies have demonstrated reductions in hypoglycemia, increased time spent in the target range, and greater levels of patient satisfaction compared with traditional fingerstick monitoring for individuals living with type 1 diabetes and T2D [7,8]. However, this technology has not been examined in the context of diabetes prevention.

In parallel, physical activity has an important role in disease prevention [9] and is a major risk factor for long-term conditions including T2D [10]. In the laboratory setting, brief bouts of physical activity have resulted in acute reductions in postprandial glucose and insulin in normal weight, overweight, and obese adults [11-13]. With commercially available technologies increasingly capable of monitoring real-time physical activity and glucose levels, it is an exciting opportunity to see changes in glucose in relation to physical activity outside of the laboratory setting. Even if people are not diagnosed with T2D, a relationship between physical activity and glucose still exists, which means those at risk could also be targeted as a preventative strategy. This approach could offer a unique opportunity for individuals to see these relationships in a *real-world* setting, which could influence their daily behaviors and subsequently their acute health.

The Sensing Interstitial Glucose Levels to Nudge Active Lifestyles (SIGNAL) study examined the usage of FGM and physical activity self-monitoring technologies for people identified as moderate to high risk of developing T2D. Secondary objectives were to assess the feasibility and acceptability of the trial design, recruitment, methodology, and technology. Interviews with participants were also conducted to understand their perspectives of using self-monitoring technologies in the context of T2D prevention. Assessing the feasibility was crucial to evaluate whether a full trial would be feasible.

## Methods

### Study Design

This trial was a randomized, 3-arm feasibility trial. The full protocol has been published [14], and the study was registered prospectively (ISRCTN17545949). All participants provided written informed consent. Loughborough University’s Ethics Advisory Committee provided ethical approval for the study (reference R17-P049).

### Participants and Setting

Participants were recruited between May and September 2017 by circulating posters, letters, and emails across Leicestershire, United Kingdom. In brief, participants were aged ≥40 years and owned a compatible Android smartphone. Participants must not have had a self-reported diagnosis of diabetes (type 1, type 2, or gestational) or a glycated hemoglobin (HbA_1c_) measurement of ≥6.5%. Interested individuals were directed to complete the Leicester Risk Assessment, which is a validated tool [15] to determine the level of risk for T2D via a Web-based survey (Qualtrics; Multimedia Appendix 1). Questions included age, sex, ethnic background, waist circumference, height, weight, and family history of diabetes. After completing the Web-based survey, individuals who received a score of moderate (16-24 points) or high (≥25/47 points) risk were contacted by a researcher by telephone or email and subsequently sent the participant information sheet by email if they were interested in taking part. An in-person appointment was scheduled at Loughborough University if participants continued to express an interest in taking part after reading the participant information sheet. During this in-person appointment, a point-of-care measurement of HbA_1c_ was taken to confirm eligibility before obtaining consent.

### Randomization and Masking

An independent researcher produced a computer-generated randomization list with 1:1:1 allocation. After the researcher confirmed eligibility, group allocation was revealed to the participant. The researcher was informed of the participant’s allocation on the day of the appointment to ensure adequate preparation (study paperwork and equipment needs varied between treatment allocations), meaning it was not possible to blind the researcher or participant to treatment allocation. Participants were encouraged to use the self-monitoring technologies as they wished, with no expectation or judgment from the researchers.

### Interventions

**Figure 1:**
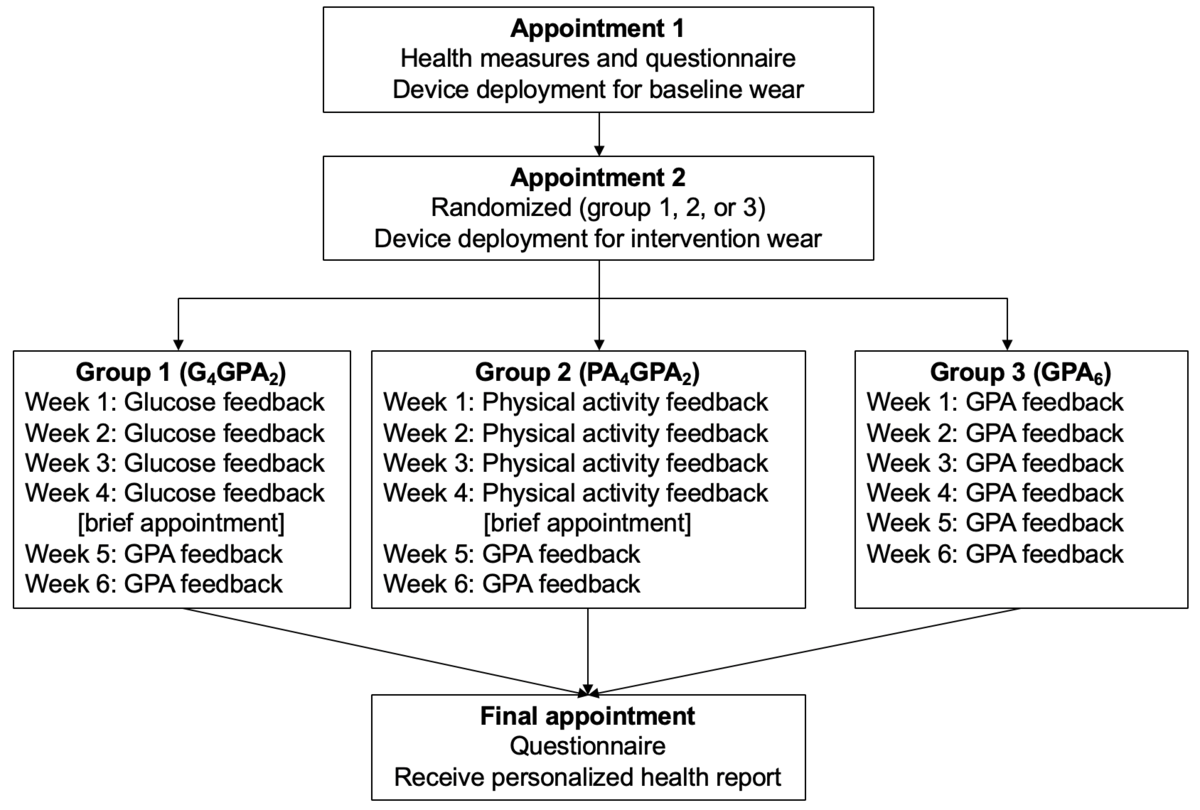
An outline of the study flow from the first appointment through to the end of participation. GPA: glucose and physical activity.

Participants were allocated to 1 of the 3 6-week interventions (Figure 1). The 3 groups were developed so that usage could be identified for participants accessing glucose feedback alone, physical activity feedback alone, and both types of feedback (in parallel). The authors anticipated that usage would be higher in group 3 than in groups 1 and 2 from the start of the intervention. Group 1 participants were given access to glucose feedback by the FreeStyle Libre (Abbott Diabetes Care) for the first 4 weeks. In the remaining 2 weeks, these participants could access physical activity feedback by the Fitbit Charge 2 (Fitbit Inc; from here on simply referred to as Fitbit) in parallel (hereon Group 1 will be referred to as G_4_GPA_2_). Group 2 participants were given feedback by the Fitbit for the first 4 weeks before also accessing feedback from the FreeStyle Libre (as well as the Fitbit) for the remaining 2 weeks (hereon Group 2 will be referred to as PA_4_GPA_2_). Group 3 participants were given feedback from both the FreeStyle Libre and Fitbit (in parallel) for the full 6 weeks (hereon Group 3 will be referred to as GPA_6_).

The 4+2-week design was used to identify how the patterns of use might differ depending on how the technologies were deployed. In particular, whether there were any additive benefits to receiving a single device initially before receiving feedback from the second device at a later point compared with being given access to feedback from both devices from the start, and how this affected their usage. This design was used to identify how usage varied by how the devices were deployed.

The Fitbit provided feedback regarding the number of steps taken, distance traveled, heart rate, calories expended, and flights of stairs climbed. Similarly, the FreeStyle Libre app provided feedback concerning glucose level (in mmol/L), direction of glucose trend (increasing, decreasing, or stable), time in range (above, below, or normal), and daily trends for individual days as well as previous 7, 14, or 28 days. Example screenshots of the feedback displayed by the FreeStyle Libre and Fitbit are provided in Multimedia Appendix 2. Feedback from the FreeStyle Libre was accessible via the smartphone app, and feedback from the Fitbit was accessible via the wrist-worn display and smartphone app. Both the FreeStyle Libre and Fitbit were worn throughout the 6 weeks, but settings were restricted or unrestricted, and monitors were masked or unmasked as per group allocation. If a participant should not access physical activity feedback during the study, the feedback was switched off (via the app, hiding the feedback icons normally displayed and switching off notifications) or covered in tape (the wrist-worn display). Restricting glucose feedback meant participants wore the sensor (as normal) but did not scan it. These participants were informed that the device was automatically logging data in the background, but as they were not scanning the sensor, no data were being stored and it was considered a nonfunctioning monitor. If we allowed these participants to scan the sensor, there was no way of restricting their access to seeing feedback.

Fitbit monitors were initialized using the Fitbit app, and minute-level data were downloaded via Fitabase (Small Steps Labs LLC) and processed using Kinesoft version 3.3.80 (Kinesoft). A minimum of 3 FreeStyle Libre glucose sensors were deployed to each participant, with each offering a lifespan of 2 weeks. Glucose levels were captured by the LibreLink app and extracted in 15-min epochs using Diasend (Diasend Inc). Interstitial glucose levels were categorized as below range (<4.0 mmol/L), normal (4.0-5.9 mmol/L), or above range (>5.9 mmol/L) [16]. Scans were also characterized as rising quickly, rising, changing slowly, falling, falling quickly, or no trend arrow (determined by proprietary algorithms).

Participants were asked to ensure that the Fitbit had enough charge and was synced regularly with the Fitbit app during the intervention. Participants were notified when the battery level reached <25% or if ≥5 days had passed since a previous sync (both remotely monitored by the researchers using Fitabase). For the FreeStyle Libre, participants were asked to scan the sensor once every 7 to 8 hours to minimize data loss but were not reminded by the researchers if they failed to adhere to this. Scanning the sensor was the only way to access glucose feedback by the participants.

### Procedures

We deployed ActiGraph wGT3x-BT accelerometers (ActiGraph) to measure physical activity over 7 consecutive days. These were deployed over the right hip, midclavicular line. ActiGraphs were initialized and downloaded using ActiLife (ActiGraph; Multimedia Appendix 2). Participants met the UK physical activity guidelines if they achieved a total of 150 min of moderate-to-vigorous (or ≥75 min of vigorous) physical activity in bouts of ≥10 min [17]. Participants were also asked to wear a Fitbit at baseline with settings adjusted and masked (notifications switched off and covered in tape) as to not provide feedback. They were instructed to wear them during waking hours and remove for water-based activities.

Self-reported age, sex, ethnic background, employment status, household income, highest level of education, and home postcode were recorded at baseline. Index of Multiple Deprivation was calculated using home postcode and was then segmented into 1 of the 10 categories ranging from ≤8.49 (least deprived) to ≥34.18 (most deprived) [18]. Height and waist circumference were measured. A digital scale (Tanita MC780MA) was used to measure weight and body fat. HbA_1c_ was assessed using a point-of-care Afinion AS100 Analyzer (Alere Inc), with prediabetes classified as having an HbA_1c_ of 6.0% to 6.4% [19]. Resting blood pressure was recorded using an Omron digital monitor (Omron Corporation).

From the start of the study, consecutive participants were invited to take part in a semistructured interview after completing the intervention. Interviews were completed at the 6-week follow-up appointment by a member of the research team independent from the quantitative data collection procedures and intervention delivery.

### Study Outcomes

#### Primary Outcome

Participant usage of the Fitbit and FreeStyle Libre were assessed by time spent on the associated apps using Ethica Data (Kitchener). Usage was also assessed by the frequency with which participants scanned the FreeStyle Libre, the frequency with which the Fitbit was synced, and the number (and type) of changes to the physical activity goals. Before participants left the appointment, verbal and written information was provided about how to apply, activate, and scan the FreeStyle Libre and how to sync and charge the Fitbit. Default Fitbit physical activity goals were 10,000 steps, 30 active minutes, 10 flights of stairs, 2500 calories, and 8 kilometers per day. To record changes to these goals, the researchers checked the participant’s study-specific Fitbit accounts daily via the Web-based Fitbit platform.

#### Secondary Outcomes

The indicators used to assess feasibility included the number of individuals who accessed and completed the Web-based survey, the number of individuals deemed eligible, uptake and retention, the number of FreeStyle Libre sensors provided to participants, and nonusage attrition [20]. Notes were made to identify the number of additional sensors provided. For acceptability, the indicators used were Fitbit wear time (defined as the presence of a heart rate signal and not categorized as sleep by Fitbit’s proprietary algorithm), the number of times the research team prompted participants to sync or charge the Fitbit, the number of minutes of missing data, and the proportion of expected data for the FreeStyle Libre.

### Sample Size Calculation

As typical with feasibility studies, no sample size was calculated, but a target of 45 participants was prespecified [14]. This approach was used because the trial sought to assess the feasibility of the recruitment processes.

### Statistical Analyses

Descriptive statistics were reported as mean (standard deviation) or frequency (%) using Statistical Package for Social Sciences version 24.0 (SPSS Inc). Semistructured interviews were conducted at the final appointment, with a convenience sample of 26 participants, and 5 further interviews were conducted to confirm data saturation. Interviews were transcribed verbatim and analyzed thematically with the support of NVivo software, version 11 (QSR International PTY Ltd). Thematic analysis comprised data familiarization; generating initial codes; searching for, reviewing, defining, and naming themes; and producing the report [21]. Members of the research team (MO and FD) conducted initial coding. Randomly allocated subsets of transcripts were coded by the remaining team members to ensure validity and consistency and to enhance interpretive authenticity. Team members met during data analyses to review emerging themes and to search for and collate participant views. Participants were contacted via email to provide feedback to ensure interpretations made by the team reflected the experiences of the participants [22].

## Results

### Feasibility of the Trial

#### Eligibility, Uptake, and Retention

**Figure 2:**
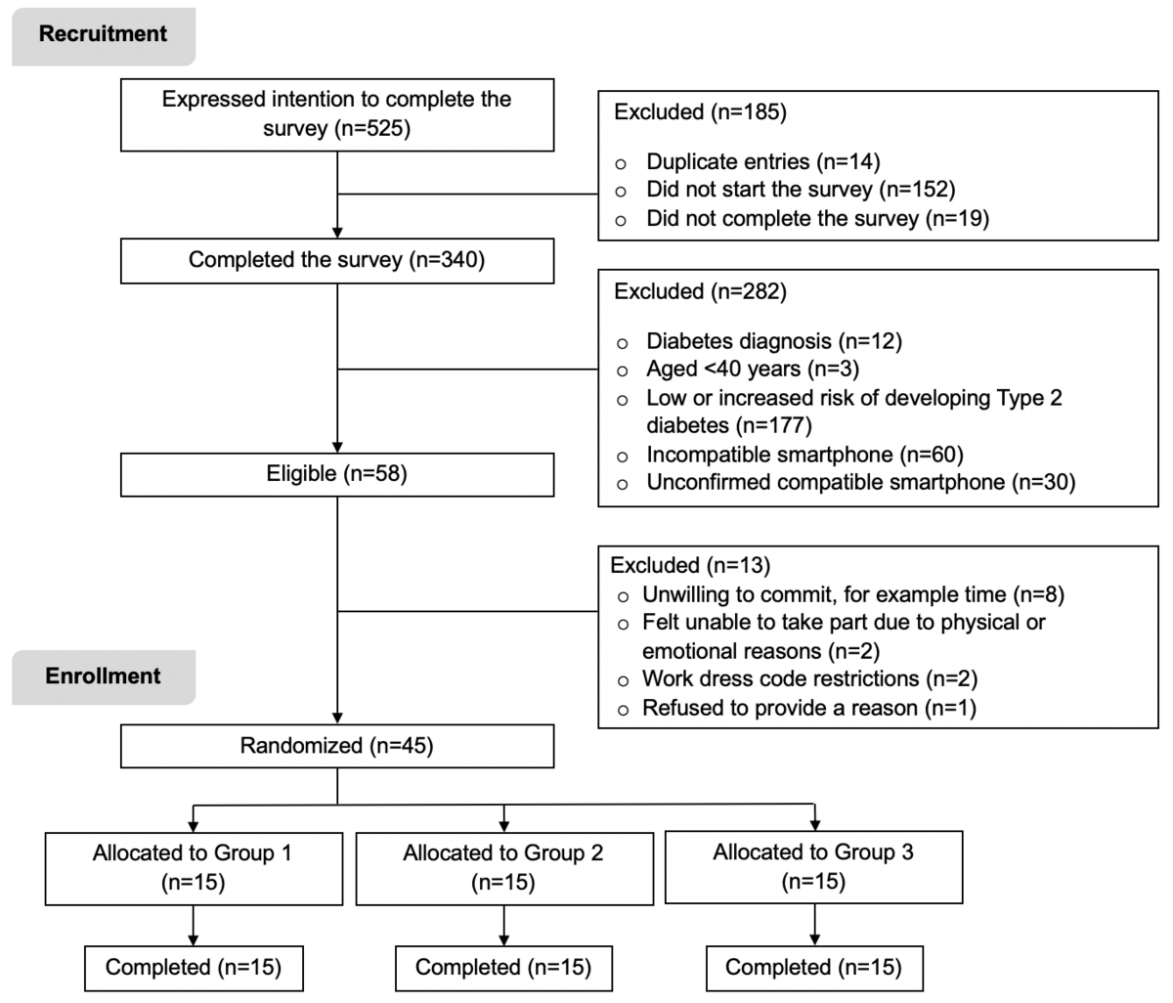
A flow chart of participant recruitment, enrollment and allocation for the study.

In total, 525 people visited the Web-based survey, 340 (64.8%) individuals completed the survey, and 58 individuals (17.1%; 11% of those visiting the survey) were eligible for the study. A total of 45 individuals (77.6% of those eligible) consented to take part, and no participants withdrew from the study (Figure 2).

#### Participant Characteristics

The sample was made up of more females (60%), the participants had a mean age of 56 (SD 8.7) years, and the participants were predominantly white British (88.9%) (Table 1). Most participants (53.4%) had completed undergraduate or postgraduate education, 19 (42.2%) had a household income of ≥£52,000, and 20 (44.4%) lived in a postcode considered least deprived. A total of 7 participants (15.6%) were identified as being at high risk of developing T2D, 3 (6.7%) were classified as living with prediabetes, 17 (37.8%) were overweight, and 23 (51.1%) had obesity.

The sample was highly compliant with wearing the ActiGraph and Fitbit during baseline. A total of 36 (80%) participants recorded 7 valid days of wear, with an average of 6.6 valid days and 861.5 min of daily wear recorded for the ActiGraph (Table 2). A total of 40 participants (88.9%) did not comply with the UK physical activity guidelines, and an average step count of 6905 steps was recorded.

**Table 1:** Participants’ baseline characteristics stratified by group.

Baseline characteristics				Total sample (N=45)	Group 1: G_4_GPA_2_ (n=15)	Group 2: PA_4_GPA_2_ (n=15)	Group 3: GPA_6_ (n=15)
**Demographics**							
	Age (years), mean (SD)			56 (9)	58.8 (9)	55.3 (9)	53.9 (7)
	Female gender, n (%)			27 (60)	6 (40)	9 (60)	12 (80)
	**Employment status, n (%)**						
		Employed		30 (67)	9 (60)	10 (67)	11 (73)
		Retired		10 (22)	4 (27)	4 (27)	2 (13)
		Other^a^		5 (11)	2 (13)	1 (7)	2 (13)
	**Education level, n (%)**						
		Postgraduate university		16 (36)	9 (60)	3 (20)	4 (27)
		Undergraduate university		8 (18)	3 (20)	0 (0)	5 (33)
		Some additional training		16 (36)	3 (20)	9 (60)	4 (27)
		Completed secondary school		5 (11)	0 (0)	3 (20)	2 (13)
	**Household income (£), n (%)**						
		>100,000		4 (8.9)	3 (20)	1 (7)	0 (0)
		52,000-100,000		15 (33)	3 (20)	4 (27)	8 (53)
		18,000-51,999		18 (40)	7 (47)	7 (47)	4 (27)
		<18,000		6 (13)	2 (13)	2 (13)	2 (13)
		Unknown		2 (4.4)	0 (0)	1 (7)	1 (7)
	**Index of multiple deprivation, n (%)^b^**						
		Least deprived		20 (44.4)	6 (40)	7 (46.7)	7 (46.7)
		Most deprived		3 (6.7)	0 (0)	1 (6.7)	2 (13.3)
**Body composition, mean (SD)**							
	Body mass index (kg/m^2^)			31.6 (7)	29.6 (5)	34.8 (9)	30.4 (4)
	Waist circumference (cm)			101.5 (15)	98.8 (14)	108.4 (15)	97.4 (13)
**Cardiometabolic health**							
	Prediabetic, n (%)			3 (7)	1 (7)	0 (0)	2 (13)
	Glycated hemoglobin (measured in %), mean (SD)			5.6 (0.3)	5.6 (0.3)	5.5 (0.3)	5.6 (0.3)
	Systolic BP^c^ (mmHg), mean (SD)			132 (16)	135.9 (15)	131.7 (16)	128.5 (16)
	Diastolic BP (mmHg), mean (SD)			81.7 (10)	82.7 (10)	79.9 (9)	82.3 (12)

^a^Other denotes looking after home and/or family, doing unpaid or voluntary work, or unable to work because of sickness or disability.

^b^Postcode deprivation offers 10 categories, but only the 2 most extreme categories have been presented for clarity.

^c^BP: blood pressure.

**Table 2:** Participants’ baseline physical activity characteristics stratified by group.

Physical activity characteristics		Total sample (N=45)		Group 1: G_4_GPA_2_ (n=15)		Group 2: PA_4_GPA_2_ (n=15)		Group 3: GPA_6_ (n=15)	
		Fitbit	ActiGraph	Fitbit	ActiGraph	Fitbit	ActiGraph	Fitbit	ActiGraph

Number of valid days, mean (SD)		6.7 (0.7)	6.6 (0.7)	6.8 (0.8)	6.8 (0.8)	6.5 (0.8)	6.5 (0.7)	6.8 (0.4)	6.5 (0.6)
**Valid day, n (%); cumulative %**									
	7	36 (80); 80	31 (69); 69	14 (93); 93	14 (93); 93	10 (67); 67	9 (60); 60	12 (80); 80	8 (53); 53
	≥6	7 (16); 96	10 (22); 91.1	0 (0); 93	0 (0); 93	4 (27); 93	4 (27); 87	3 (20); 100	6 (40); 93
	≥5	0 (0); 96	3 (7); 98	0 (0); 93	0 (0); 93	0 (0); 93	2 (13); 100	0 (0); 100	1 (7); 100
	≥4	2 (4); 100	1 (2); 100	1 (7); 100	1 (7); 100	1 (7); 100	0 (0); 100	0 (0); 100	0 (0); 100
Wear time (minute/day), mean (SD)		865.1 (69.6)	861.5 (86.9)	912.4 (63.3)	911.4 (88.5)	832 (61.1)	833.2 (74)	851 (60.7)	839.8 (80)
Counts per valid wear minute, mean (SD)		—^a^	328.7 (144.6)	—	342.2 (107.6)	—	281.3 (123.1)	—	362.5 (187.5)
Step count per day, mean (SD)		8575 (4530)	6905 (3776)	9329 (4251)	7331 (3433)	7650 (4007)	5637 (1963)	8747 (5367)	7748 (5148)
Sedentary (minute/day), mean (SD)		—	540.1 (95.3)	—	569.9 (90.1)	—	536.6 (89.9)	—	513.9 (103.2)
Light physical activity (minute/day), mean (SD)		—	288.2 (83.4)	—	304.4 (97.1)	—	271.4 (77.3)	—	288.7 (76.7)
MVPA^b^ (minute/day), mean (SD)		—	33.1 (28.4)	—	37 (21.2)	—	25.2 (18.6)	—	37.2 (40.5)
MVPA in bouts ≥10 min (minute/day), mean (SD)		—	10.1 (21.9)	—	9.6 (14.4)	—	5 (6.2)	—	15.8 (34.5)
Met physical activity guidelines^c^, n (%)		—	5 (11)	—	2 (13)	—	1 (7)	—	2 (13)
Active minutes^c,d^, mean (SD)		39.8 (41.5)	—	39.5 (28.2)	—	43.5 (54.1)	—	36.9 (42.9)	—
Number of floors climbed^d^, mean (SD)		11.8 (9.5)	—	13.8 (8.2)	—	7 (6.4)	—	13.8 (11.7)	—
Reminders to move^d,e,f^, mean (SD)		3.6 (1.5)	—	3.3 (1.7)	—	3.9 (1.6)	—	3.6 (1.2)	—

^a^Not available from the device.

^b^MVPA: moderate-to-vigorous physical activity.

^c^Active minutes are calculated by Fitbit’s undisclosed proprietary algorithm but are said to represent activity of ≥3 Metabolic Equivalent of Task (METS) and are only awarded if duration of activity is ≥10 consecutive minutes.

^d^ActiGraph accelerometers do not capture a comparable variable.

^e^Reminders to move track hourly step counts, and notifications delivered with a slight vibration are sent when the user does not reach 250 steps by 50 minutes of each hour (between 09:00 am-5:00 pm).

^f^No notifications were active during this wear period; this intends to suggest how many would have been shown had the settings been unmasked.

### Feasibility of the Technology: Quantitative Insights

A total of 22 participants (48.9%) completed the study using the minimum number of 3 FreeStyle Libre sensors. Moreover, 11 of the 23 participants requiring extra sensors (47.8%) had 1 faulty or misplaced sensor, 8 (34.8%) had 2, 2 (8.7%) had 3, and the remaining 2 (8.7%) had 4. A total of 262 displacements were reported and there were more in groups G_4_GPA_2_ and GPA_6_. It was noted that 27 participants (60%) set the LibreLink app to remind them to scan the glucose sensor. There were no instances of nonusage attrition.

### Feasibility of the Technology: Qualitative Insights

Participants evaluated the technologies (a main theme) and discussed the usability, wearability, reliability, durability, preferences, privacy, and cost (as subthemes). Additional quotes to support the described subthemes are presented in Multimedia Appendix 3.

#### Usability

Participants generally found both technologies easy to use, in particular, how they found the Fitbit easy to charge and were complimentary about the battery life. Comments relating to applying the FreeStyle Libre emphasized the initial trepidation felt by the participants, mostly because of the visible needle and concern that the insertion process would be painful (across all 3 groups). However, there was a pleasant surprise with which the FreeStyle Libre adhered to the skin, and this nervousness typically subsided following the first application. The requirement of scanning the FreeStyle Libre at least every 8 hours to avoid data loss was highlighted as a flaw with the technology, as some participants forgot to scan regularly enough, and several participants noticed periods of missing data during sleep:

I thought it was all very straightforward, all very easy.

female, GPA_6_

I can take it off at night and charge it at night so there's no issues with that. I put it on charge every second night really. Charging a Fitbit lasts about five days so if I put it on every second night it was fine; never had no issues with it running out at all.

male, PA_4_GPA_2_

I was pleasantly surprised that it didn’t hurt. When I first saw the thing I thought, God this is going to be awful and hurt my arm but I couldn’t believe how painless it was.

female, GPA_6_

When you go to sleep, seven hours isn’t all that long really. You generally need more than seven hours’ sleep and not everybody would wake up in the night. I do but not everybody would, so that wasn’t quite long enough.

female, GPA_6_

#### Wearability

The majority of the participants found the FreeStyle Libre comfortable, with many forgetting the sensor was there (particularly participants in groups G_4_GPA_2_ and GPA_6_). A few indicated issues with skin irritation from the Tegaderm and having trouble applying the sensor correctly. The issues with the wearability of the sensor appear to relate more to participants being self-conscious about wearing the sensor, with some resorting to covering it up with additional or alternative clothing and others suggesting refinements to the sensor and Tegaderm. Similarly, participants across all groups noted that the design of the Fitbit could be improved. In particular, participants suggested aligning the design with traditional watches, and for some, this issue meant they were not pleased when asked to replace their existing jewelry with the Fitbit. Reasons for discomfort were also related to the impact of temperature on the Fitbit strap:

It was fine though, I think it’s brilliant. I forgot it was even there and in fact I’m a bit lost without it now it’s not there, it is quite strange.

female, GPA_6_

It was more about that but when I had the glucose monitor on, for a start, when it was hot, I put sleeveless on but then I was getting, what’s that, what’s that, what’s that? So, I started wearing things with just sleeves on.

female, PA_4_GPA_2_

Because I have got all these lovely watches at home that I can’t wear, because I have got that. Somebody at work has got one of those on, I noticed she has got it on her other hand. She has got her watch on one hand and her Fitbit on other hand. I thought well that’s one way of doing it. But I couldn’t wear it on there.

female, GPA_6_

#### Reliability

Some participants questioned the glucose level provided by the FreeStyle Libre when it classified them as *low* or in the *red* when this was not normally the case. They tended to attribute this observation to the timing of using a new sensor or altering the location of the FreeStyle Libre on the arm. When talking about the Fitbit, the accuracy of the data provided by the technology was questioned (by the participants across all 3 groups), specifically for step count, calories, distance, mode of activity, stairs climbed, heart rate, and sleep. During masked Fitbit wear, very few problems with automatic syncing were revealed, but during unmasked periods, many participants reported the need for multiple attempts at manually syncing the Fitbit. Similarly, multiple attempts were sometimes needed to scan the FreeStyle Libre:

I think when I first got it, it was quite low actually for the first…well for the first day, it was below the 4 and I was thinking ooh but then it went down to the normal sort of range. And actually, the last one a week ago and the same thing actually, that’s been lower, I don’t know if it’s the device or if I’ve done something different or what.

female, G_4_GPA_2_

I think sometimes it’s been quite generous with steps. Turning around to get the towel is giving me steps and some days it said, “you’ve walked ‘x’ miles” and I thought, “I can’t have.” Some days it’s told me I’ve walked eleven miles and I thought, “have I walked eleven miles?” Eleven miles is quite a…so I’m not sure it’s accurate to that extent.

male, G_4_GPA_2_

I had a couple of issues…sometimes it's a bit difficult to link it to my phone, it just takes a bit of perseverance rather than everything happening on one flick of the screen, it might take two or three minutes just to catch on the Bluetooth. It's not a major issue, it's just a little niggle, that's probably a better word.

male, PA_4_GPA_2_

#### Durability

Several participants spoke about their experience of having the FreeStyle Libre sensor fall off, and, in part, this was because they were getting less aware of wearing it as time went on (more so for participants in G_4_GPA_2_ and GPA_6_). Particular reasons reported by participants included walking into door frames, catching the FreeStyle Libre sensor on clothing, or showers and perspiration weakening the sensor's attachment. Participants also raised annoyances about the memory of the FreeStyle Libre, suggesting that the memory is not sufficient, and perhaps, if it lasted a day or two, it would avoid some of the data losses, as some participants kept forgetting to scan it regularly:

I caught one on the car doorframe when I was getting something in and out of the car. The other one, I walked into a doorframe and caught it and it just bent the needle and it stopped working.

male, GPA_6_

I thought it might have a better memory, last a day or two but because I kept forgetting to, because I am so busy I just kept forgetting to scan it so there are gaps and also at night time I go to bed like 9, 10 o’clock at night time and then get up sort of 12 hours later after a really good sleep and there are huge gaps.

male, G_4_GPA_2_

#### Privacy

Participants discussed the topic of privacy, explaining how they did not mind or have any problems with having data collected on how much they used their phone and the study apps during the study period. Some participants mentioned that this was mainly because they had nothing to hide. Others raised concerns about other apps and their monitoring activities, such as Google Maps, which captures a lot of information about user location:

It was interesting that you could have all this stuff going on on your phone but it also is a bit spooky as well because people are watching your performance. I suppose that, in real life, can be quite scary. It is anyway - you don’t know who’s watching you at any point.

female, GPA_6_

#### Preference

Participants described accessing an array of feedback metrics for their activity. Preferred Fitbit features were typically step count and heart rate, with calories burned considered the least meaningful. There were conflicting remarks about the Fitbit prompts, the associated *motivational* messages were seen as childish and receiving the notification to move hourly was deemed too frequent. Nonphysical activity features such as food logging and relaxation were not used as often or considered as useful as the physical activity features of the Fitbit. This was typically because of the manual entering of data required on the app or preference for using alternative apps. The FreeStyle Libre features were talked about positively by participants with no clear preferences for the feedback provided. A suggested improvement was for greater flexibility in viewing historical data. There was an overall preference toward the FreeStyle Libre compared with the Fitbit, mostly because of the novelty of monitoring glucose levels (regardless of group):

I’d quite often look at my heartbeat because that was the most interesting thing on there.

male, GPA_6_

It gives you those daily reports which are helpful. And I thought it was helpful that you could add in a note when you had eaten something. So, you could then align, I mean in the end you didn’t really need to because you could see the patterns for yourself, but to start with that’s useful that you could put in when you had eaten something. Or put a note in of some sort which is helpful.

female, PA_4_GPA_2_

I have actually enjoyed the glucose monitor results, looking at the graphs and charts more than the Fitbit.

female, GPA_6_

I could have delved a lot more deeply into the Fitbit but I was more interested in the app and the glucose levels…I’ve never monitored my glucose before and I was fascinated by the whole thing. I was amazed how the device worked and all sorts of things about it.

male, G_4_GPA_2_

#### Cost

Several participants expressed their concern about the cost of the FreeStyle Libre sensors if they were to buy them, in particular, because they only last 2 weeks, which would restrict many people from accessing this technology:

It is a lot. I am sure they will come down as people use them more and more, but, and they only last two weeks, so you know it is a lot of money.

female, PA_4_GPA_2_

### Acceptability of the Technology: Quantitative Insights

During the 6 weeks, 22 of 45 participants (48.9%) provided 42 days of valid Fitbit wear, with all participants averaging a total of 40.1 (SD 3.2) valid days. Compliance with syncing the Fitbit data noted that 12 participants (26.7%) received a prompt from the researchers to sync (encourage data transfer), whereas 5 (11.1%), 3 (6.7%), 2 (4.4%), and 2 (4.4%) participants received 2, 3, 4, or 5 prompts, respectively. In terms of charging the Fitbit, 9 (20%) participants received a prompt to charge the Fitbit as battery status reached <25%. No data losses were recorded for the Fitbit across the 3 groups. The level of data capture for the FreeStyle Libre was high—an average of 87.6% (SD 3.8) and 82% (SD 19) in the first and sixth week, respectively—and this was relatively consistent between the 3 groups (Multimedia Appendix 4).

There was no clear trend toward increased physical activity over the 6 weeks using step count, active minutes, number of flights of stairs, or reductions in the number of reminders to move (Multimedia Appendix 5). Similarly, there was no improvement in interstitial glucose levels using time in range (Multimedia Appendix 6).

### Acceptability of the Trial: Qualitative Insights

Participants evaluated the study design (a main theme)—specifically, having a positive experience, wanting full access to feedback (in particular, participants in the groups G_4_GPA_2_ and PA_4_GPA_2_), indicating the study duration being too short, having issues with the eligibility criteria, and being uncertain of what was expected of them (as subthemes). Additional quotes to support the described subthemes are presented in Multimedia Appendix 7.

There was an overall sense of positivity in taking part in the study. Indeed, participants wanted access to the information provided by the technologies, with some explaining their frustration of having devices masked and others describing their disappointment with not being in GPA_6_. The 6-week duration of the study was deemed not long enough by some participants to gain a full understanding of the relationship between their lifestyle and glucose, represent normal life, set more challenging Fitbit goals, or try out different wear locations for the FreeStyle Libre:

I really enjoyed it. I really learned a lot from it.

male, G_4_GPA_2_

Yes, I was really pleased that I was in the six-week group because I thought that will give me a lot to go on, whereas two weeks is kind of neither here nor there. I thought six weeks is a reasonable amount of time to assess things. I thought that was good for me and it led me to sort of draw some conclusions about glucose and how I was using it.

female, GPA_6_

It is difficult to do that over six weeks. Perhaps over several months you might be able to pin it down to what you eat and when you eat it. Perhaps keeping a food and exercise diary and linking them together, but we weren’t asked to do that.

female, GPA_6_

Some participants suggested that the FreeStyle Libre might be better suited to individuals at greater risk of developing T2D or people already with a diagnosis of diabetes. Only including individuals with a compatible Android smartphone was highlighted as an important limitation of the study:

I said to [the researcher] you would have to change what app you use or something because I think you potentially would get a lot more people involved with it if it was compatible with an iPhone as well.

female, GPA_6_

There was uncertainty on 2 fronts—changing behavior and engaging with the technology. There was some uncertainty around whether participants felt that the study required them to change their behavior or whether it was purely a monitoring or data collection exercise:

If I had been told, like what we are looking for is you to increase your activity because we are after this certain, we are trying to see this thing, then I would have gone for it.

male, G_4_GPA_2_

There were also inconsistencies in participants' perceptions of how much to engage with the technologies. Some individuals suggested that receiving more instructions on how to use and interpret the feedback would have been useful, whereas others were happy about not being given too much guidance and felt that the instructions given were clear. That said, participants explained how explicitly being told to be more active or to eat differently would have left them better placed to act on the feedback. Other suggestions included directing people to sources of information and being able to compare their glucose levels against a healthy profile via the FreeStyle Libre app:

Perhaps a bit more detailed, again just how to just sort of really make the best use of it would be better.

female, PA_4_GPA_2_

There’s not really that much information to go with the monitor to tell you how to interpret the data.

male, GPA_6_

But [the researcher] did say at the start, use this as you want it’s your, your thing to use as you want. So that was good.

male, G_4_GPA_2_

### Technology Usage

From weeks 1 to 6, the groups G_4_GPA_2_ and GPA_6_ spent a lower amount of time on the FreeStyle Libre app, going from 28.3 to 12.3 min per day and 11.5 to 5.5 min per day, respectively (Table 3). PA_4_GPA_2_ participants logged 7.1 min per day in week 5 and 4.7 min per day in week 6. A similar pattern was observed for the FreeStyle Libre app whereby participants in groups PA_4_GPA_2_ and GPA_6_ observed a reduction in time spent on the Fitbit app, reducing from 6.7 to 3.4 min per day and 7.6 to 3.9 min per day, respectively. Similarly, participants in G_4_GPA_2_ reduced their app usage from weeks 5 to 6 from 16.9 to 12.7 min per day (the only weeks when they could access it).

**Figure 3:**
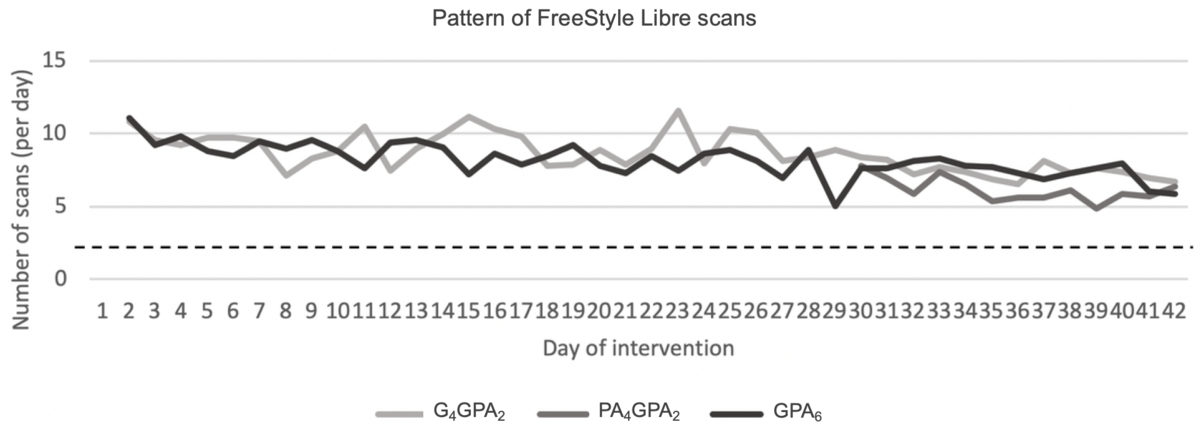
Pattern of participant scans of the glucose sensor over the 6 weeks, with the horizontal line reflecting the recommended minimum number of scans per day. One participant recorded 173 scans on day 12, deemed an outlier, and so was excluded from this figure. G_4_GPA_2_: Group 1; PA_4_GPA_2_: Group 2; GPA_6_: Group 3.

The average number of scans declined over time across all 3 groups (Figure 3). In the groups G_4_GPA_2_ and GPA_6_, participants logged on average 9.4 scans per day in week 1 and 6.8 scans per day in week 6. Across weeks 5 and 6, participants in PA_4_GPA_2_ conducted 6.3 scans per day and 5.6 scans per day, respectively. A number of Fitbit monitors unexpectedly restored to default settings during deployment, resulting in syncs being completed automatically.

A total of 13 of 45 participants (28.9%) changed ≥1 of the physical activity goals from the default settings. Of these participants, 9 (69.2%) changed the daily step goal, whereas the number of floors, active minutes, calories, and distance goals were changed by 5 (38.5%), 3 (23.1%), 2 (15.4%), and 2 (15.4%) participants, respectively. Notably, the daily step goal was reduced by 7 participants (77.8%).

**Table 3:** Pattern of app usage for the Fitbit and FreeStyle Libre.

App usage		Group 1: G_4_GPA_2_	Group 2: PA_4_GPA_2_	Group 3: GPA_6_
**Fitbit, mean (SD)**				
	Week 1, minutes per day	—^a^	6.7 (3.9)	7.6 (3.8)
	Week 2, minutes per day	—	5 (4.7)	4.6 (3.9)
	Week 3, minutes per day	—	3.9 (2.8)	5.3 (5.0)
	Week 4, minutes per day	—	3.1 (1.8)	5.5 (3.7)
	Week 5, minutes per day	16.9 (16.4)	3.5 (2.1)	4.3 (3.3)
	Week 6, minutes per day	12.7 (13.6)	3.4 (2.6)	3.9 (3.4)
**FreeStyle Libre, mean (SD)**				
	Week 1, minutes per day	28.3 (23.6)	—	11.5 (8.5)
	Week 2, minutes per day	21.7 (22.6)	—	7.7 (5.7)
	Week 3, minutes per day	17.6 (14.7)	—	7.7 (7.9)
	Week 4, minutes per day	13.2 (11.2)	—	6.3 (8.2)
	Week 5, minutes per day	8.2 (5.5)	7.1 (3.9)	6.0 (5.5)
	Week 6, minutes per day	12.3 (17.6)	4.7 (6.4)	5.5 (6.0)

^a^Data not available.

## Discussion

### Principal Findings

To the authors’ knowledge, this is the first study to deploy both behavioral and physiological self-monitoring digital health technologies to individuals at risk of developing T2D. Use of the devices reduced over the 6-week intervention period but remained higher than the minimum level of use needed to avoid data loss (eg, scanning the FreeStyle Libre every 8 hours). Despite limitations with smartphone compatibility, it was feasible to conduct the study with a high uptake rate and full retention of participants at the end of the trial. The study and technologies were acceptable to individuals at risk of T2D.

### Specific Recommendations

This feasibility trial has identified several key areas for industrial and research sectors to collectively consider when considering the use of commercially available technologies in health research. These include the following: the need to (1) integrate, or facilitate the integration of, information collected by behavioral and physiological sensors into a single platform and (2) provide intelligent feedback and automated, actionable insights by using multiple data sources in real time.

### Technology Usage

Participants were given minimal instruction as to how to engage with the technologies in an effort to reflect an *off the shelf* deployment. There was reduced use of the Fitbit and FreeStyle Libre over the 6-week intervention period, which was similar between the 3 groups. This suggests that the provision of both technologies did not clearly impact the *honeymoon* period or the novelty effect frequently observed when people newly access digital health technologies [23]. For the FreeStyle Libre, the magnitude of this novelty effect was different between time spent on the app and the frequency of scanning. Scans per day reduced by approximately 28% between week 1 and week 6, whereas time spent on the app decreased by 52% to 57% for both groups accessing the FreeStyle Libre for the full 6 weeks. With participants expressing preference for more simple feedback features, these data suggest that participants ended up preferring to simply scan and see their current glucose levels rather than delve into the more detailed feedback that could be seen by the Freestyle Libre app [24,25]. This appeared to happen once users were content with the instant, and perhaps more understandable, feedback provided with each scan.

Stand-alone telemonitoring systems are an encouraging tool to support motivation [26]. The dual deployment of physical activity and physiological monitors succumbing to the issues of disuse may have been because of the feedback sources treated as distinct entities with distinct purposes. It has been noted elsewhere that relationships between behavior and health vary in how easily they can be detected by the individual [26]. This means that it may be more difficult for people to notice the benefits of being more physically active or having better blood sugar control compared with, for example, improving asthma medication adherence to relieve acute episodes of breathlessness [27].

A potentially helpful technological development would be to combine and relate the feedback provided by the Fitbit and FreeStyle Libre (or other similar devices) into a single module or feedback interface to better represent the acute relationship between behavior and physiology. With such integration, the technology may be more equipped to identify, display, and describe *teachable moments* such as “the walk you have just taken after eating bought your blood sugar back down to baseline 20 minutes faster than if you had stayed sitting down.” Elsewhere, teachable moments have been defined as naturally occurring events that may motivate individuals to adopt positive, risk-reducing behaviors [28]. However, the authors propose that to display feedback showing the physiological consequences of behavior, it might need to be handled in a slightly different way to make it meaningful to the individual. Previous studies have used glucose monitoring technology to show activity-related reductions in glucose to adults living with T2D [29]. The development of such biobehavioral feedback messages will require sophisticated real-time machine learning techniques to capture and process data to produce appropriate notifications to users in an accurate and timely manner. Another provision of *teachable moments* would be to demonstrate these relationships under controlled conditions. The positive impact of physical activity bouts on acute health in the laboratory setting has been demonstrated [11,12] and can highlight key stimulus-response events. Bailey et al [30] used intermittent continuous glucose monitoring to show individuals with prediabetes and T2D their blood glucose as a tool to increase adherence to an exercise program over 8 weeks. However, this approach would not be currently feasible at the population level, which is where further technological development may still play a key role.

### Feasibility of the Trial and Technology

Eligibility of individuals completing the Web-based risk survey was low (17%), with 63% of those who were ineligible for the study classified as having a low risk of developing T2D and 32% having a noncompatible smartphone. Therefore, this recruitment approach mostly attracted the *worried well* and may benefit from more targeted strategies, such as recruiting from primary care records that have identified 17.5% to 26.5% of screened individuals presenting with impaired glucose regulation [31,32]. However, since study completion, the FreeStyle Libre is now compatible with iOS devices, eliminating the limitation of smartphone compatibility that excluded many otherwise eligible survey responders. This was also highlighted by participants as a huge barrier to recruitment and a source of selection bias. That said, once recruited, all participants completed the study, describing their experience of taking part as a positive one.

Characteristics of the participants also offer important insights into the appropriateness of the recruitment strategy and eligibility criteria. Although most participants were classified as overweight or obese and did not meet physical activity recommendations, less than one-fifth of the sample was deemed at *high risk*, only 7% were prediabetic, and there was significant ethnic homogeneity, with almost 90% of the sample being white British, despite recruiting from an area with a multi-ethnic population. This is particularly important given that South Asian adults are 1.4 times more likely to develop T2D than white British adults [33]. Therefore, the next trial must actively seek more at-risk communities. After experiencing the FreeStyle Libre for at least a couple of weeks, many participants saw the glucose monitor as having great potential for people diagnosed with T2D. The FreeStyle Libre has since been made available on prescription by the NHS for people who meet a clear criterion (including currently undertake intensive monitoring >8 times daily and have an impaired awareness of hypoglycemia) with nationwide availability from April 2019 [34].

Participants expressed the need for further instruction and demonstration of the technologies and assistance with interpreting the feedback. The reported uncertainty about engaging with the technologies and hesitation in changing behavior clearly demonstrate that simply providing people with digital health technologies is insufficient for proactive lifestyle modification [35]. Although this was not the primary aim of the study, the minimal guidance approach to the study has illuminated important issues with using sophisticated digital devices in this way. The need for a human element in such prevention approaches to provide education, demonstrate devices, prescribe exercise, or motivate individuals to make positive lifestyle changes is something current digital health technologies cannot replicate or replace. In part, this may be because interfaces are not adequately intuitive or because data are not easily interpretable. Interviewees expressed difficulty knowing whether their glucose pattern was normal and what approaches they should take to improve it. Similarly, many features of the Fitbit and FreeStyle Libre went unexplored or were regarded as surplus. Participants tended to focus on features regarded as more palatable such as step count and heart rate, which are often featured in wearable technologies [36]. Therefore, with ever more sophisticated commercially available devices, important challenges are to ensure the information provided to users is easily accessible, intuitive, and actionable.

### Acceptability of the Trial and Technology

The technologies were perceived as comfortable and easy to use, and there was much positivity around participants’ experience of the study, with some criticisms of the trial design linked to wanting the study to last for longer than 6 weeks as well as disappointment in not receiving the combined feedback for the full length of the intervention period (ie, being allocated to GPA_6_). Adherence to wearing the Fitbit was excellent, as was charging compliance. However, issues with syncing the Fitbit data left participants frustrated and led the authors to conclude that syncing events should be interpreted with caution and were not a suitable measure of usage.

The reliability of the feedback was also bought into question, particularly the numbers presented by the Fitbit, which seemed to classify extraneous arm movements as *steps*. A comparison of baseline step count values from the Fitbit and research-grade ActiGraph may suggest an overestimation from the Fitbit. Commercially available monitors have shown strong correlations with research-grade accelerometers [37], but this does not mean numbers can be directly compared. Consequently, this perceived or real overestimation may partly explain the lack of a clear change in physical activity from baseline because of the potential for a false sense of achievement. Similar notions, although to a lesser extent, were made for the FreeStyle Libre. Some participants noticed interdevice variability and the occasional need for multiple scanning attempts, but most issues were related to the visibility of the FreeStyle Libre and the aesthetics of the device itself. The most common complaint of the FreeStyle Libre was its durability, as supported by more than half of the participants requiring at least one additional sensor. Previous studies using the FreeStyle Libre have also noted concerns with the adhesion of the sensors and have highlighted instances of mild skin irritation and bruising often linked to medical-grade adhesives [7,30]. Efforts to avoid the need for additional sensors are needed to minimize disruption for patients and costs to the NHS.

### Strengths and Limitations

SIGNAL was the first study to deploy self-monitoring wearable technologies presenting behavioral and physiological feedback in real time to individuals at risk of developing T2D, classified using a validated screening questionnaire. The mixed methods design of the trial enabled real-life context to be applied to quantitative data, permitting a more in-depth assessment of participant’s perspectives toward the study design and digital health technologies. The group allocations allowed all participants to experience feedback from both devices, adding to the breadth of experiences contributing to insights from the qualitative interviews. The objective measurement of physical activity allowed the sample to be compared against UK physical activity guidelines.

In addition to the limitations previously disclosed, the study had a relatively small sample size, limiting insight from statistical analyses. Assessor and participant blinding to group allocations was not possible. In addition, it was not possible to quantify what participants specifically looked at within the Fitbit and FreeStyle Libre apps. Owing to its main purpose as an intervention tool, it was not possible to set up the FreeStyle Libre in a masked mode (as achieved with the Fitbit). Consequently, no glucose data were collected from the PA_4_GPA_2_ group for the first 4 weeks of the intervention period. Other studies have used the FreeStyle Libre Pro model that only has a logging mode but is not currently available in all countries [38].

### Conclusions

SIGNAL, to the authors’ knowledge, was the first study to explore levels of use when providing combined behavioral and physiological self-monitoring wearable technologies to individuals at risk of developing T2D. This study highlights several important areas for future research, notably (1) the inclusion of a more diverse pool of individuals at risk of developing T2D or identified as living with prediabetes and (2) the detection and presentation of teachable moments linking behavioral choices with acute physiological consequences to individuals in controlled and free-living settings. Improvements to the usability, wearability, reliability, and durability are needed before such approaches to disease prevention can be implemented into routine health care. However, issues around the integration of feedback from multiple sensors and the need for real-time, actionable feedback need to be resolved for any future trials.

## Appendix

Multimedia Appendix 1 Online survey for assessing the risk of developing type 2 diabetes.

Multimedia Appendix 2 Application screenshots and device deployment settings.

Multimedia Appendix 3 All quotes pertaining to the technologies.

Multimedia Appendix 4 Additional Freestyle Libre data.

Multimedia Appendix 5 Additional Physical activity data.

Multimedia Appendix 6 Additional Freestyle Libre data#2.

Multimedia Appendix 7 All quotes pertaining to the wider study.

Multimedia Appendix 8 CONSORT-EHEALTH checklist (V1.6.1).

## Abbreviations

FGM: flash glucose monitoring
NHS: National Health Service
SIGNAL: Sensing Interstitial Glucose Levels to Nudge Active Lifestyles
T2D: type 2 diabetes
