# Supplementary material for: Examining the Use of Glucose and Physical Activity Self-Monitoring Technologies in Individuals at Moderate to High Risk of Developing Type 2 Diabetes: Randomized Trial
Source: JMIR Mhealth Uhealth. 2019 Oct 28;7(10):e14195. doi: 10.2196/14195 (PMC6913728; doi:10.2196/14195)
Supplement: Multimedia Appendix 1 [file mhealth_v7i10e14195_app1.pdf]

Online survey for assessing the risk of developing type 2 diabetes

What is your gender? Male (1) Female (0)

How old are you? 49 years or younger (0) 50-59 years old (5) 60-69 years old (9) 70 years or older (13)

What is your ethnic background? White (0) South Asian (6) Black (6) Chinese (6) Mixed ethnicity (6) None of these (6)

Do you have a parent, brother, sister and/or own child with diabetes? Yes (5) No (0)

[if male] What is your current trouser waist size in inches?* e.g. 32, 34, 36 36, 38, ...

[if female] What is your current trouser size?* e.g. 10, 12, 14, 16, 18, ...

How does this clothing size fit you?* Loose / Just right / Snug / Don't know / Refused

What is your weight in kg?

What is your height in metres?

**Rather than ask for a specific waist circumference, participants were asked about their clothing size and clothing fit to estimate waist circumference as it is often a question that individuals do not know the answer to.*
