# Supplementary material for: Examining the Use of Glucose and Physical Activity Self-Monitoring Technologies in Individuals at Moderate to High Risk of Developing Type 2 Diabetes: Randomized Trial
Source: JMIR Mhealth Uhealth. 2019 Oct 28;7(10):e14195. doi: 10.2196/14195 (PMC6913728; doi:10.2196/14195)
Supplement: Multimedia Appendix 2 [file mhealth_v7i10e14195_app2.pdf]

Screenshot showing feedback from the Fitbit and Freestyle Libre apps

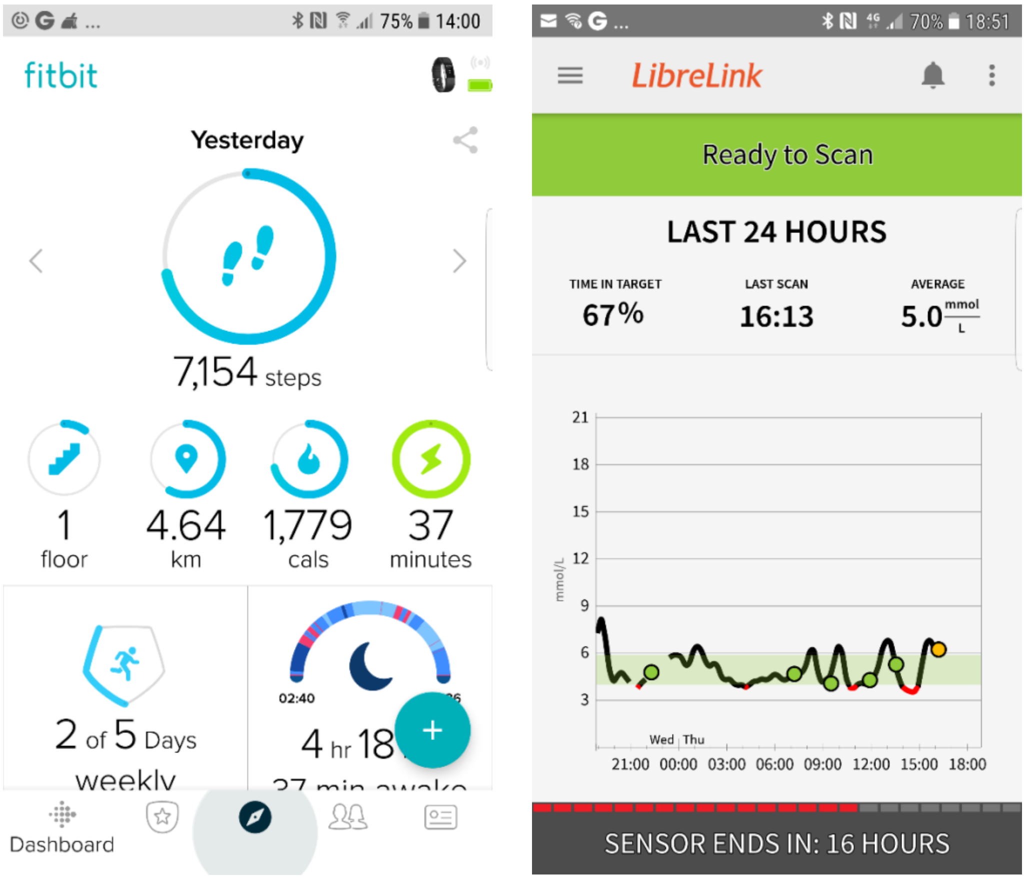

Device deployment

Table. An outline of the data collection procedures for the 3 self-monitoring technologies deployed.

	ActiGraph wGT3x-BT	Fitbit Charge 2	Abbott Freestyle Libre
**Devices**	24 devices used	45 devices used	176 devices used<br>0 handheld readers used
**Sample rate**	100 Hz (.gt3x file format)	60 seconds	900 seconds
**Epoch**	60 seconds	60 seconds	900 seconds
**Initialisation**	Deployed in delay mode on day 0. Commenced logging on day 1 at 00:00:00; stop time applied 7 days after	Commenced logging data at point of initialisation on day 0	Commence logging data 1 hour after point of initialisation on day 8 (Groups 1 – G_4GPA_2 and 3 – GPA_6) and day 29 (Group 2 – PA_4GPA_2) and after any additional sensors fitted
**Deployment**	Fitted by participant (on day 0) with guidance from researcher	Fitted by participant (on day 0) with guidance from researcher	Fitted on by participant (using step-by-step instructions and guidance from research team) on day 0 of intervention to inform subsequent self-deployment of sensors
**Location**	Anterior hip, mid-line of the right thigh	Non-dominant wrist	Upper portion, non-dominant posterior brachium
**Wear duration**	Baseline: 7 days (10,080 epochs)<br>Intervention: Not worn	Baseline: 7 days (10,080 epochs)<br>Intervention: 42 days (60,480 epochs)	Baseline: Not worn<br>Intervention: 42 days (60,480 epochs)

**Wear instructions**	Continual wear except removal for sleep and water-based activities	Continual wear except for sleep and water-based activities	Continual wear (24hr) with adhesive tape over sensor
**Charging**	Not required	Requested to charge overnight every day	Not required
**Non-wear**	≥60 min of consecutive zeros with allowance for 2 minutes of interruptions coded as non-wear.	An absence of heart rate signal.	Lifespan of sensor not met (expected duration of 14 days) and any missing data between sensors.
**Valid day criteria**	≥10 hours of valid waking wear time	≥10 hours of valid waking wear time	≥90% of data points per day
**Valid file**	Baseline: ≥4 valid days<br>Intervention: Not applicable	Baseline: ≥4 valid days<br>Intervention: ≥24 valid days	Baseline: Not applicable<br>Intervention: ≥4 days
