# Supplementary material for: Examining the Use of Glucose and Physical Activity Self-Monitoring Technologies in Individuals at Moderate to High Risk of Developing Type 2 Diabetes: Randomized Trial
Source: JMIR Mhealth Uhealth. 2019 Oct 28;7(10):e14195. doi: 10.2196/14195 (PMC6913728; doi:10.2196/14195)
Supplement: Multimedia Appendix 3 [file mhealth_v7i10e14195_app3.pdf]

Box. All quotes pertinent to the technologies.

**USABILITY** G_4GPA_2 PA_4GPA_2 GPA_6
**Easy to use/finger prick/not waterproof**
"I think it's quite straight forward, so it says you haven't much time and the targets and when you had the low glucose. I think it's quite intuitive and easy to use" (female, G_4GPA_2)
"It was all very simple. It's a really good innovation, the whole thing" (male, G_4GPA_2)
"It's such an easy tool to use, it's a brilliant thing" (female, GPA_6)
"I thought it was all very straightforward, all very easy" (female, GPA_6)
"Well, and the steps really just to know, I mean I downloaded the pedometer to my phone but that was a matter of switching it on each time which is a bit more of a faff. Whereas this is just there, you don't even think about it, it's just on your arm, you don't even think about it. So, yes, the counting of steps really is what I also was hoping to be able to see more easily" (female, GPA_6)
"Yes, it was quite straight forward. You had to sync it make sure it was working. It was quite easy to use and I knew how to use the sensor. It was alright" (female, PA_4GPA_2)
"Fitbits or variations are now becoming very common. People totally accept that technology. Really totally accept it" (male, G_4GPA_2)
"Especially the blood glucose monitor, I think it's just an amazing invention. I feel a bit like a Cyborg kind of thing. And I didn't realise to start with that it would measure it through your clothing. So, the fact that you can do it through your clothing is just amazing" (female, PA_4GPA_2)
"I have been swimming with it, I have been in the shower with it. It hasn't affected me at all" (female, PA_4GPA_2)
"It was partly the technology, but it was that you can check things and you can monitor your progress in a way that is difficult to just do from memory. You could look back at all that sort of data" (female, GPA_6)
"I looked at the Fitbit, just because it's a different interface. Out of interest because I like my tech. I have knew about it, it's a beautiful interface. I can see why people buy them. That's a lovely interface, it really is. Very easy to use" (male, G_4GPA_2)
"I think it would be good because people don't necessarily like the thumb prick, because it's not nice, it is quite tender and you have got to rely on doing that. Whereas this is in all the time so the worst thing they have to do is put the phone next to it" (female, GPA_6)
"It is good because it is always there, whereas the alternative is that you have to take everything with you and have a shot. I imagine the cost of glucose monitor is a lot more expensive, but to me they would be brilliant" (female, GPA_6)
"Must be a lot nicer than having to prick your finger and put blood on the thing and then you don't get the constant feedback, like you do with the sensor" (female, GPA_6)
"I love the ease of use. I wouldn't have done the finger prick test for six weeks. I wouldn't have volunteered for the study to do that, even if you'd said six times a day. I have done that with my wife's, not with the same needle and after a while … this is so easy to use" (male, G_4GPA_2)
"It's just as accurate as doing the blood on your finger but a lot less intrusive. That's the other thing for me of course, if it was intrusive, because I have never had to prick my finger. However, many times people do it. That seems a lot less intrusive that doing that, to me... But having that connected directly to your watch or to that in some manner would be, because then you have got the, can rely on the alarm thing. That's to me the technology is to provide a safeguard to people as well" (male, G_4GPA_2)
"I'm not aware that there's a needle in there, because a needle is an issue for some people but it's certainly much better than pricking your finger frequently for those people so yea" (male, PA_4GPA_2)

"Knowing it was there and you didn't have to have all the performance with needles or whatever. As I say, having seen my dad do it but knowing that, like I say, you could check it any time and then when you did check it oh that's not too bad, that's okay" (female, GPA_6)

Putting it on

“Somehow it just seems squeamish to some people but I don’t know why because it’s a little film that you have got to fire in quickly so it doesn’t bend as it’s going in. I had no problems, I loved doing it, thoroughly enjoyed it” (female, GPA_6)

“I was pleasantly surprised that it didn’t hurt. When I first saw the thing I thought, God this is going to be awful and hurt my arm but I couldn’t believe how painless it was” (female, GPA_6)

“It was so easy to put in and to take off” (female, GPA_6)

“Absolutely no problem at all. It didn’t matter if it went in the same hole or near the same hole, there’s nothing to show now” (female, GPA_6)

“Of course, I saw the size of that needle but actually it’s … I don’t know how they get it in to be honest because it’s so fine but it’s flexible as well. How on earth that manages to get shot in, I don’t know. It’s extraordinary” (female, GPA_6)

“After that I was fine because it didn’t hurt or anything. The first time I did it myself I thought, ‘oooh’ but it was absolutely no problem. You just push it against your skin and press and it’s all over. So easy” (female, GPA_6)

“I’m not bothered with that. I’m fine with needles. If it was my daughter she would have passed out. I just did it myself” (female, GPA_6)

“Needles don’t bother me at all. I had bowel cancer fifteen years ago. You have that many tubes and bits of … no, it doesn’t bother me. It doesn’t hurt at all. It was easier to put it in there than it was to try and put it in there because you can see it. It’s hard there because of the bone. I could just press it and it just went straight in” (female, PA_4GPA_2)

“I didn’t like changing it. Well you know, it’s a needle going in but it was a flexible needle. I didn’t understand how it worked really, I thought if it was a flexible needle and it sort of shoots it into your arm, why doesn’t the flexible needle just bend away. I was also fascinated that it was only about 2mm long this needle and how could it monitor my glucose at that level” (female, G_4GPA_2)

“I had to place it in the mirror and then look away. Not that that you can see anything because it’s in the capsule but I didn’t like doing it. I was always relieved after I had done it and it wasn’t too bad. I suppose if I was doing it for a long period of time I would just get on with it and do it” (female, G_4GPA_2)

“Because when I first put it on here my arm really bled to start off with. So, we had to take it off and put it somewhere different” (female, PA_4GPA_2)

“So, when I was away, after the first two weeks I was away, I had to take it off and put it on when I was away. So, I was really worried that the same thing was going to happen. So, I had got a spare one with me if it did. So, I had to take that all with me in the luggage and everything. And then just putting it on, it was, I don’t know, I don’t like it. And my daughter, it freaked her out. Even though she worked in the hospital, she kept saying ‘oh I don’t like this, I feel really sick’, she thought it was a needle permanently stuck in. She didn’t like it. And I was conscious of it in bed, sort of lying on it, I just didn’t like it. It’s quite sort of cumbersome really” (female, PA_4GPA_2)

“I was a chicken when it had to be put in. If you speak to [the researcher] she’ll tell you, I was crazy. It took about 20 minutes and she was asking if I was ready and I just kept saying no. She then asked me to do it myself and I couldn’t do it, I was just stood there in the mirror like this and she said do you want me to do it, I said yes but not yet, I’m not ready. I apologised and eventually she did it for me and then it fell off. I was thinking sod’s law this would be me, the one who hates it” (female, PA_4GPA_2)

“I was still nervous when she had to take it off and I was quite a baby coming in and taking it off. I wasn’t a very good candidate in that way” (female, PA_4GPA_2)

“I did consider if I was ever perhaps going to get one in the future” (male, GPA_6)

"I wouldn't be persuaded to carry on using it but you could do, if you wanted to, I suppose" (male, G_4GPA_2)

"It was fine. It was actually, even the first time I thought this is going to hurt because when you see the size of the needle, even though I am an acupuncturist but actually you don't feel a thing. It ached a little bit the first time, my arm, but otherwise it was fine" (female, GPA_6)

"I got Alan, my husband, to just locate it for me a little bit because it's an awkward place, yeah, and that's it really… Yeah, I got him to place it and then I pressed it on… Because he could tell…, well I could have gone upstairs in the bathroom and done it myself and located but it was just easier to, he was sitting next to me on the sofa, I said just put it around there and he made sure it was in the area of the previous one, so yeah. It's quite easy" (female, GPA_6)

"And it is quite big. I mean I know it's only... but it is quite big. But I thought the whole, the packaging and the insertion thing and everything, that is so clever. Maybe over-engineered, but very clever" (female, PA_4GPA_2)

"I'm a complete wimp about needles, so I think if I could do it, everybody could do it" (female, GPA_6)

"The first time I put it on was at the research facility and it went on perfect" (male, G_4GPA_2)

"Fine, no problems and it was very painless. I didn't notice inserting it, really… Yes, I'd do it or the wife would help me on occasion. No problem with that at all. Quite frankly, you couldn't feel it hardly" (male, GPA_6)

"Couldn't have been easier with the machine. There was no pain at all from the pin going into the skin. The pain, well not pain but uncomfortness, was the outer rim of the machine, the tool you use to put it in with and that's just a bit of uncomfort. As soon as you took it off, that disappeared anyway" (male, PA_4GPA_2)

"When you look at it, it's quite a big needle actually. It's maybe going in with a spring-loaded effect. It seemed fine, to be honest" (male, GPA_6)

"Not so keen about the jabbing though. I don't mind the Fitbit but those needles, otherwise everything was fine" (female, PA_4GPA_2)

"I had a bit of an ache there to begin with when the first one went in but it soon went away within a day and the subsequent times I didn't feel it at all" (female, GPA_6)

"The first time you are aware of it. No, you don't know it's there because it's not pain and it's like someone does that and it's over and done, it doesn't hurt" (male, G_4GPA_2)

"I think the fact that it goes into your, at the moment you have got that little sensor going into your arm as such, means you are limited where you can have it. Because if you had it anywhere and you are low, then you couldn't catch it. I get there's a thing. Obviously, I don't know, I am not a medic, where else measures, which is easy to measure. But if you had something connected to the bottom of the watch, doesn't actually make a hole in your arm" (male, G_4GPA_2)

"The only thing that caused me any discomfort was the plasters or the covers put over the top because sometimes they stretched. The second time I put it in, I decided to shave my arm as well because it perhaps made it a bit easier, especially taking it off" (male, GPA_6)

Requirement to scan/sync

"That was something I found a little bit onerous having to check the time as the day went through, as well as the hours. It's surprising how quickly the hours in a day go. I was thinking good grief it's time for my 4 o'clock" (female, GPA_6)

"When you go to sleep, seven hours isn't all that long really. You generally need more than seven hours' sleep and not everybody would wake up in the night. I do but not everybody would, so that wasn't quite long enough" (female, GPA_6)

"The memory. Maybe they'll get better on that. Maybe it would be better if it wasn't quite so short as seven hours" (female, GPA_6)

“It gives you an audible warning, which is good. It’s like a text coming through, so that was helpful because then you’re prompted to look at it and see what’s happening. I soon got to know that that was telling me that it needed scanning. I didn’t have a problem with that” (female, GPA_6)

“I just thought, ‘I’m not doing it enough’ because it was saying ‘average of three or five’, although I think I’ve gone down at the end anyway. Sometimes it was because I had left my phone somewhere” (female, GPA_6)

“The only thing I did notice, I don’t know whether I did something to the timer or something, but it didn’t always go through the night. So, I would scan it before I went to bed, but sometimes there was a break in the night. So, when I scanned it in the morning, there was a gap. So, I don’t know what I done” (female, PA_4GPA_2)

“It probably got lots of dots and big dashes then a dot or lots of every hour. It is just remembering, it would be good if it was automatically scanning all the time, which it probably was before” (female, PA_4GPA_2)

“When I was doing the readings at night time, it wasn’t a problem it was just remembering to do one and the other” (male, PA_4GPA_2)

“I think if you look at my record, you will find a few gaps but the gaps were never more than a small gap. I sometimes failed if I collapsed into the tent at night and went to sleep at nine, I probably didn’t do a reading seven hours later. I was quite conscientious about it. It’s surprising how time flies when you’re really involved in a thing like that. The seven hours goes by in almost no time at all, I think” (male, G_4GPA_2)

Charging

“Mostly, I charged it every night but sometimes I didn’t worry if I hadn’t put it on charge every night. Before I came home, for instance, I charged it the night before and then I knew it would last a few days, so I didn’t bother until the last night I charged it again” (female, GPA_6)

“It’s good that it does have at least a few days’ charge because sometimes it’s not convenient or something happens that you can’t get it on charge” (female, GPA_6)

“I sometimes forgot but it seemed to go for days without needing charged so I didn’t worry too much but it was always at night when I charged it. It didn’t take it off to charge it during the day” (female, G_4GPA_2)

“It's good because it's got a long charge on it so I would generally put the Fitbit on charge when say I was watching telly, I knew I was going to be sitting down watching telly for a couple of hours so I knew that I wouldn't be doing anything anyway and it charges up really quickly. So, that’s how I incorporated that” (female, GPA_6)

“Battery life seemed to be quite good. It wasn’t a nuisance to have to think about it. Overnight was fine” (female, GPA_6)

“I can take it off at night and charge it at night so there's no issues with that. I put it on charge every second night really. Charging a Fitbit lasts about five days so if I put it on every second night it was fine; never had no issues with it running out at all” (male, PA_4GPA_2)

“It didn’t ever discharge. I think it would last five days or so, without being recharged” (male, G_4GPA_2)

“It was fine, yes. I just used to take it off at night time. I was charging it every night to start with but then I stopped bothering with that. I think it would probably last a week without charging it anyway. I put it on charge when it dropped down a bit. That was fine, really” (male, GPA_6)

“Yes, sort of when it drops down I was just putting it on. I think it shows you that it’s orange on the app when it’s dropped down a bit and I just put it on charge overnight when it had dropped down to orange” (male, GPA_6)

“More with my Fitbit, that was the worst one, I forgot it three times, so that didn’t go on...Putting it on charge at night, you’ve got to remember to pick it up in the morning before you go out. It was usually the days I went, out if I was in a rush, I quite often forgot to unplug it and put it on, so that was a bit of a pain. I would have preferred to just have left it on and not have to do that every night...A couple of times this week I’ve forgotten to put this on until lunchtime and then it’s like, ‘oh no’” (female, GPA_6)

“Going back to the app, there was the daily measures as well. I didn’t tend to use it so much on the sleeping because I tended to keep it charged up overnight, even though quite often I don’t think it really went down below 80% anyway, if I did sleep in it” (male, GPA$_6$)
“I put it to charge every night as [the researcher] told me, and because the lead on it is so very short, it’s only about 12 inches long, I had to have it on the floor on the other side of my bed with the idea that I would put it on as soon as I got up in the morning but I kept forgetting...I would get up and I would be having my breakfast and I would think I have probably done 200 steps and I haven’t put it on. I would have certainly done 2 staircases as well and it hadn’t recorded them. I found that quite annoying. Then I would be a bit disheartened for that day” (female, G$_4$GPA$_2$)
“It’s got a very short lead as well. I think that might be a problem for some people. I think it’s only got about 30cm length for that lead. Depending on where your plug is, you can’t leave it dangling, you need to have to have it on a surface. It your plug’s not in the right place you can’t put something under it, it would be difficult, so maybe a longer lead would be a good idea” (female, GPA$_6$)
**WEARABILITY**
**Comfort**
“It irritated me for the first two or three days. It was when the weather was really hot and it got really sweaty. It irritated me to start off with but otherwise it was fine, it was just like having a watch on” (female, GPA$_6$)
“It was fine though, I think it’s brilliant. I forgot it was even there and in fact I’m a bit lost without it now it’s not there, it is quite strange” (female, GPA$_6$)
“I did have a problem with it sticking on for a while but that was when it was really hot” (female, GPA$_6$)
“Although I had got the Tegaderm to go over the top, when it was really hot I had to ask [the researcher] to bring me some more Tegaderm, because I ran out” (female, GPA$_6$)
“I did have slight problem with the second one I put in. Whether it was just settling into a comfortable place, it was a little bit uncomfortable for the first day but otherwise that really made even more nervous because I would forget about it. Then I would suddenly remember and think God, it is still there?” (female, GPA$_6$)
“I thought it was something magical, it was wonderful, just amazing. I didn’t know of its existence at all. I thought, ‘what a marvellous device’. When it’s in, you’re not aware of it” (female, GPA$_6$)
“I did find that it gets very sweaty under there with these rubber … On hot days, I didn’t particularly like that bit because it’ll suddenly stick but generally, it was quite good” (female, GPA$_6$)
“Having the senor on was fine. I didn’t really know it was there, unless you looked. It was quite unobtrusive. You didn’t feel it, so in that way, that was good” (female, GPA$_6$)
“The only thing with this is in the hot weather [the Fitbit] does get hot and sweaty” (female, GPA$_6$)
“I’ve got very sensitive skin and the buckle metal bits where irritating me at times. Especially if I got a bit sweaty, that was unpleasant. I wouldn’t say often but just if I felt warm. I would find sometimes at night I would sit scratching at it, I found myself putting my fingers under the buckle so it wasn’t actually touching my wrist” (female, G$_4$GPA$_2$)
“And I didn’t like the, I just didn’t like the feel of it, I didn’t like it there… I was aware it was there all the time” (female, PA$_4$GPA$_2$)
“And when I was taking the last one off, I don’t know if I had not put it on properly, but it was quite wet underneath. Whether the water had got in when I went swimming or something? Even though it had got the clear things on the top. So, whether that was why it was itching. But it was really itchy towards the end. I was worried that it had sort of quite lifted a bit, and the needle had came out and sort of. That’s why, in Email, [the researcher] says is the data still coming through, and it was. I was worried that it was not properly in. I just didn’t like the thought of it. It felt a little bit bruised. Yeah, a bit bruised” (female, PA$_4$GPA$_2$)

“The Fitbit because it's a plastic strap, it does get a bit sticky, a bit sweaty at times so that's irritated me at times, I've loosened it off at times a bit, but apart from that” (female, GPA_6)
“It is ok, she did it the first time, the first tab I had and it was bleeding. I had to go back to the office and the girls were asking what I had done and I said I had a tab put in. They then asked why it was all bleeding down my arm and I replied, I hadn’t looked at it. It was fine after I had cleaned it” (female, PA_4GPA_2)
“It was a bit uncomfortable the first few days. The first day I had it, I walked into the door. In the evenings, it was fine. I think the first few days I was conscious about sleeping on it, because I knew there was a needle there I think it was that fear that the needle was going to poke into me, but I knew it was safe. After the first few days I forgot about it, after I got used to having some alien thing stuck in my arm” (female, PA_4GPA_2)
“The first couple of days I was worried about sleeping on it but after that I forgot about it and sleeping was fine and it didn’t bother me” (female, PA_4GPA_2)
“The first sensor, I have had 3 sensors haven’t I. The first sensor was uncomfortable, I mean not massively, but I could feel it every so often. But the other two I have not felt them at all apart from when you bang them on a doorway and it’s not comfortable. I have not felt them at all. I haven’t thought about them. They have not irritated me. They have been fine” (female, PA_4GPA_2)
“It was perfectly fine, it was much more comfortable than I thought it would be. I didn’t really notice it a lot of the time. I wasn’t really conscious it was there” (female, GPA_6)
“In fact, people would say, ‘what’s that?’ and I’d think, ‘oh yes, of course, I’m wearing it’” (female, GPA_6)
“No, no in fact you tend to forget it is there so it didn’t really irritate me at all except when I caught it on something” (male, G_4GPA_2)
“Yeah, on the odd occasion I put long sleeve shirt on it was like yeah odd. More noticeable it was there” (male, G_4GPA_2)
“Fine, yes. I don’t notice it… The only thing with it was with the probe, I didn’t realise I bumped into so many things so often because you’re conscious of the fact and you’d hear it” (male, GPA_6)
“I think it was a bit more to do with the coverings and that caused me a little bit of irritation” (male, GPA_6)
“The only thing was the cover plasters irritated my skin a bit and they’re a bit difficult to put on sometimes. They tend to stick to themselves and are totally useless, not being practised at the technique” (male, GPA_6)
“No problem wearing it. You can hardly see it, well I can’t see it and if I can’t see it, it doesn’t bother me. That was the only thing, the positioning. You’re not aware of it and you forget it’s there sometimes because it’s fairly comfortable to wear” (male, GPA_6)
“I couldn’t really relax and I wasn’t very relaxed in the bath because I had to keep my elbow out of the water...because I had lost one, knowing how expensive they were, I was thinking no, don’t do that again. I was thinking of keeping this in place and I was being very careful getting dressed and undressed trying to make sure I didn’t move it or lose it” (female, GPA_6)
“Was a bit worried about the thing sitting there in my arm and I was worried about knocking it, I did knock it a few times but it was fine, it didn’t hurt or anything” (female, G_4GPA_2)
**Visibility**
“It was more about that but when I had the glucose monitor on, for a start, when it was hot, I put sleeveless on but then I was getting, what’s that, what’s that, what’s that? So, I started wearing things with just sleeves on” (female, PA_4GPA_2)
“I wasn’t really aware of it. I wore just the same clothes as I would normally” (female, GPA_6)
“I’ve found it quite interesting with all this. I’ve really enjoyed checking up on my glucose. Obviously, not having this. You couldn’t wear short sleeves with that right there” (female, GPA_6)
“The first time, I wore a cardigan if we went out in the evening. I suppose it was the social aspect. In the day, it didn’t matter but in the evening when you’ve got something nice on, you don’t particularly want a great big blip on your arm” (female, GPA_6)

"Although it seemed to stick very well there and it was good to do that, I think it would be better if it was somewhere else…I suppose you could have it on your leg but I don't know whether that would be bad as well…In a lot of ways, it was a good place to have it because it was stuck there but it is a little unsightly in the summer. In the winter, it wouldn't have been a problem" (female, GPA_6)

"Yeah, I started sort of covering it over" (female, PA_4GPA_2)

"I was a bit pleased in some respects because I was walking around town with the monitor stuck on my arm which people could see, I was a bit conscious of people looking. If I was with my wife she would say that's where I plug him in at night and make a joke of it. It wasn't a problem and just when she said no, don't worry about it, I thought, great it's off now and back to normal, whatever that is" (male, PA_4GPA_2)

"You know what it's like, walking along and somebody has got a white disc stuck to their arm and they are going to look and say what is that? They wouldn't say anything to me, I'm not embarrassed by it but if I could have had t-shirts with longer sleeves, bearing in mind the weather has been nice and I wouldn't have worn a jumper or anything. Within the first couple of weeks when I was in the garden I wore a sport support to cover it, but it was a bit tight and I could feel the pressure on my arm. I think you still have been getting the readings" (male, PA_4GPA_2)

"But one imagines as life, as technology improves, they will find a slightly less intrusive sensor. Which will perhaps be attached to the watch itself. You know where you don't have that, and literally people can do, an alarm goes off on the watch or on the phone, and it says your blood sugar level, you know if you are having to treat it for one or two, your blood sugar level is at this level, you need to take action." (male, G_4GPA_2)

"Possibly but you can see it through the shirt anyway to a certain extent. Even with short sleeves on it was covered up anyway" (male, GPA_6)

"I was a bit paranoid about knocking it and also with my granddaughter, I had to end up wearing long-sleeved tops because I didn't want her to see it. She was just going to pull it. In that way, it was a pain but generally, it was fine" (female, GPA_6)

"I think, whether I had put it in the wrong place or, it was quite noticeably when it was sort of here. And because it was the summer and it was hot, in the winter it wouldn't be an issue. Because it was, obviously I like wearing sleeveless tops and short sleeves, it was just noticeable. In the photographs I have got this big white patch on me. Which isn't great" (female, PA_4GPA_2)

"It's the fact because it was rigid plastic, and you could feel it. It just wasn't really a nice thing to wear. But, if it had been the winter time it probably wouldn't have been so bad. I think because it was hot and the fact you could see it in all the pictures. I never thought about it. If I had thought it I could have stood the other way around" (female, PA_4GPA_2)

"I have moved them up my arm progressively because I didn't like it being too low, because when you wear a short-sleeved top someone can see it. But you know what I don't really care, I wasn't really... and it was quite nice to sort of say to somebody... 'I am taking part in this research... it's really interesting'. So, I think it's amazing" (female, PA_4GPA_2)

"Yeah, it was quite amusing. I suppose that you don't really look at it, it's a bit out of the way, it's quite low down so short sleeve tops or whatever. I could see people looking at it but I'm used to it now" (female, G_4GPA_2)

Aesthetics

"I suggested that the sticky patch you put over it should have some kind of pattern on it, so it was like a tattoo, so it was like the centre of a flower or something...I thought that might make it look like the middle of a flower and then petals round it on the plaster. You could stick that on and it would disguise the fact … It would just look like a tattoo. That might help people if they were feeling conscious about it and wanted to cover it up when they are swimming or anything like that...It might be for some people, if you weren't really confident. Some people don't like evidence of their problems being on show, do they? If it was disguised like that it might help because I would imagine it would be quite easy to print something on that transparent plaster. Because it's transparent it would look like a tattoo, as it's a very thin layer. That's my invention by the way. I'm going to patent that one. Nobody nick that...It didn't bother me but I thought other people might be a bit … Some people are a bit nervous, especially younger people, if you are on the beach or if you've got a sleeveless top or something. Tattoos are so accepted now, especially the coloured ones. They look wonderful, so I thought that might be perhaps a good thing" (female, GPA_6)

“I wear watches jewellery as well as timekeeping pieces but I wear it as a piece of jewellery and I found that really difficult, I've really missed wearing watches, I've got two or three watches, and I found that quite difficult...I got used to it in the end and my mum said would you like one for your birthday and I said no. I like it from the point of view of monitoring, particularly the steps, that was interesting, but from a point of view of wearing something like that, that doesn't appeal to me at all. I'm just funny like that” (female, GPA_6)
“The only drawback to it is that it’s not aesthetically very nice. I would like it to be a bit nicer to look at...I don’t know but it’s just a bit boring. I just would like something a bit more … maybe if you could change the strap. You couldn’t do that because of the things at the back, the green light things...that’s just a woman’s point of view. It’s not very pretty. You wouldn’t want to go out for dinner in it. That’s the only drawback really...When I went to Liverpool to see my friend, we went out for dinner one night and I didn’t wear it. I had done my 10,000 steps, so I didn’t feel guilty. I took it off because it didn’t go with what I was wearing because it was somewhere very nice...I was just thinking it would be better if it was something you could hide under your clothes. Like a pendant, like for people that have to get help. You could tuck it away and you could still … I don’t know if it would work...What I tend to do is wear it further up and cover it up and I can still wear my other jewellery then” (female, PA_4GPA_2)
“Maybe it would be different for men but for women, if that was flesh-coloured, I think you wouldn’t be as conscious of it” (female, PA_4GPA_2)
“Make it slimmer. Because it sticks out quite a bit. And perhaps a flesh coloured, because it’s bright white. And I suppose it’s got to be rigid plastic hasn’t it, if it was softer I don’t know. If there was any way it could have been like bendy. But I suppose it wouldn’t do the job would it” (female, PA_4GPA_2)
“The device is quite big, I don’t know if you would want something a bit more discreet. I thought it was quite a good little device to have. I think I’ll be missing it now” (female, PA_4GPA_2)
“Because I have got all these lovely watches at home that I can’t wear, because I have got that. Somebody at work has got one of those on, I noticed she has got it on her other hand. She has got her watch on one hand and her Fitbit on other hand. I thought well that’s one way of doing it. But I couldn’t wear it on there. I wouldn’t get one because I want to wear a watch watch. A proper watch... I only kept that on for the research. I lived with it, except at night. The only time I took it off was at night” (female, GPA_6)
“I think it’s amazing technology. Absolutely amazing. Fitbit however, I guess I have got issues with that. One is I always wear a normal watch anyway. I can’t bear digital time, I have to have analogue. So, you have to wear two watches, and it’s like, ‘why are you wearing two’” (female, PA_4GPA_2)
“I guess the frustrations that I had, I guess one of the questions I was thinking was ‘Do you have to have the Tegaderm on it’ and I don’t know whether that was just for this research to do, and to keep it a bit more waterproof or whether you could do without that, because that’s a bit unsightly. Especially when it’s been on a few days, it gets quite raggedy. Whether you could just have it without that” (female, PA_4GPA_2)
“It was quite chunky, chunkier than my normal watch would be but that was the only downside really” (female, GPA_6)
“I did enjoy both of them though and my daughter has a spare Fitbit and I might ask her if I can use it, I don’t know. I did find it a bit more comfortable to wear. It was quite a chunky one whereas I know you can get much more elegant ones” (female, G_4GPA_2)
“It’s a comfortable thing to wear and it looks quite smart” (female, GPA_6)
"The device is quite big, I don’t know if you would want something a bit more discreet. I thought it was quite a good little device to have. I think I’ll be missing it now" (female, PA_4GPA_2)
**RELIABILITY**
**FL accuracy**
“I think when I first got it, it was quite low actually for the first… well for the first day, it was below the 4 and I was thinking ooh but then it went down to the normal sort of range. And actually, the last one a week ago and the same thing actually, that’s been lower, I don’t know if it’s the device or if I’ve done something different or what” (female, G_4GPA_2)

“So, since I’ve put this one it’s been quite low, whereas before, so that’s where I put the new one in. I don’t know if that’s just a variation between the sensors or we can do something differently” (female, G_4GPA_2)
“I noticed when the sensors are new they drop below into the red and I really did drop into the red” (male, G_4GPA_2)
“I dropped into the red there. That was in the night during sleep. So, I have went into the red there but it could have been a new sensor. A new sensor yeah, it was” (male, G_4GPA_2)
“It’s quite interesting looking at the technology because I’m sure on occasions it didn’t read properly because it was showing me as below the … it didn’t measure anything, it just said ‘low’. There’s no change in the way I felt, I felt fine” (male, GPA_6)
“I did go through all the low events. I’d just look at them and I’d think, ‘the monitor’s going wrong a bit there'” (male, GPA_6)
“I wore it first of all on this arm, then on here, then I had a problem with this arm, so it had to go on that again and then on here. I felt the readings I got from my left-hand side, on the whole, were lower than what I got from my right-hand side. I don’t know whether that is the case but looking at it, I felt that was the case, which is no science at all. I don’t know whether you’ve got all this data but you might look and see if there is a pattern” (male, G_4GPA_2)
“There was just that feeling that this side tended to five lower readings but I don’t know” (male, G_4GPA_2)
“I noticed obviously a strong correlation between, on some occasions, going below the green channel and how it felt to be low on glucose. Sometimes it wasn’t that noticeable and I must confess that I did tend to suspect that maybe the sensor was getting towards the end of its span. It might have been faulty but it’s easy to blame the tool. I don’t know whether that would have been the case or not. Do you find sometimes that sensors do tend to degrade somewhat towards the end of their period?” (male, G_4GPA_2)
“It was very reliable and the last one worked up to the very end as well” (male, G_4GPA_2)
"I don't know if the last monitor was reading lower, but I was ill, sorry, I will go back to the beginning there, I had soup that first Friday because you know we left to get to Taunton, and that night my blood sugar really dipped. So, I don't know what that was about" (female, GPA_6)
**FB accuracy**
“With regard to the calories, I looked at the calories burned and initially, I couldn’t understand because I have never burned that many calories. That was quite surprising to see how many calories you can burn without particularly doing much. I was a bit dubious about that one” (female, GPA_6)
“Occasionally I did find it a little bit inaccurate. On the days I have done marathons, my overall kilometres and the end of the day has come out at round about thirty-nine” (female, GPA_6)
“Obviously a marathon is forty-two and I have walked bits either side of it and also occasionally when I was in the car it thought I was on a bike, it would give me calories for having ridden a bicycle when in fact, I was sitting in a car” (female, GPA_6)
“I have heard people say this before, but one of the other things I do is that I knit a lot. I was actually picking up steps for knitting because the wrist and arms movements, when I wasn’t actually going anywhere” (female, GPA_6)
“Not really, it just made me feel that perhaps it’s not really as accurate as it should be. I have no idea if it would be possible to adjust the Fitbit, whether you have to tell it your stride length over a certain distance, I really didn’t go into any of that” (female, GPA_6)
“I did accidentally, record me as sleeping once. I got up one day, it could have been one of the days after the marathons, and I was a bit wrecked. I got up and I put the Fitbit on but then I went back to bed. It had me sleeping for just over an hour I think, but that was the only bit sleep it recorded but I wasn’t actually asleep I was just lying there on the bed relaxed” (female, GPA_6)
“An earlier one I got was the longest London Underground line which was interesting, I do like odd facts. Obviously, I ran or walked that distance to give me those steps. Again, I am a little bit suspicious of that because of the inaccuracies I found for example when I was knitting and not going any distance and just confusing the Fitbit” (female, GPA_6)

“Sometimes it didn’t seem to respond so well. It didn’t seem to recognise all these steps and cheated me out of a few. I know you were able on the app to make it record quite strenuous activity, like cycling, can’t you? I didn’t do any cycling or running but sometimes I was digging my garden and it was giving me no credit for it at all. I suppose I should have turned something on maybe, I don’t know. I thought I’m using all this energy and calories and it doesn’t know about it because I am only doing a few steps. I suppose that is my fault, I probably should have investigated how I record that” (female, G_4GPA_2)
“I’m not obsessed by it but when it’s there and you have got the information you think, this is what happened overnight when I was totally unaware of it and there it is recording. I didn’t understand how it could record whether you were in a light sleep or a deep sleep or whether it’s something to do with your pulse. Anyway… I did just find it interesting to see” (female, G_4GPA_2)
“I've also thought I walked quite fast, even with the dog and when the dog's off the lead I tend to walk quite fast and my husband tells me and I always was led to believe that if you, this'll sound silly, I told [the researcher] this sometimes, I sometimes sing when I'm walking and then if it's difficult, if you can't just sing but you're starting to puff then I've always understood that your heart is working at a good rate and the Fitbit didn't seem to indicate that I was which concerned me” (female, GPA_6)
“Actually after a few nights of recording the REM, it did not give me the detail. I kept it on and then it would, it didn’t seem to… If I go to that, I don't know, I can show you. Yeah, it just sort of said how many hours slept but it didn’t give me this kind of read out” (female, GPA_6)
“The calories, I think calories expended. I don’t really know how you could do that. It could be figure just worked out and that’s it. I don’t know how you get 5,000 calories from walking around” (male, G_4GPA_2)
“What I have been doing there is looking at my resting heart rate and exercise rate, I know it’s not accurate but it’s an indication and it’s great” (male, PA_4GPA_2)
“So, that’s, I haven't been looking at steps particularly on that simply because I wear another one and but I have noticed, when I have looked, on occasion that, that there seems to be a difference, I don’t know if there’s much, much difference in, what’s the word, accuracy between these two, but I did notice that there was a difference in steps and I can only presume, unless you told me different that the dominant had, non-dominant had there, it makes the big difference, hence I’m glad to put that on my left wrist today” (male, PA_4GPA_2)
“...so if I wave my arms around a bit more, I might do a few more steps” (male, PA_4GPA_2)
“There is a guy who I help out on a Saturday with his garden for most of the day and when I said I had done so many steps, he said well I don’t believe you and I replied look it’s there. I might have done 10,000 and on his it was only 4 because his is actual steps. I don’t try and work it out, it is what it is. When I look at that and it says you have done 22,000 steps and you have walked for 20 kilometres, I mean I would be on my back if I walked 20 kilometres” (male, PA_4GPA_2)
“I did some ironing and because I’m right handed, the other hand which I am wearing the Fitbit is doing nothing” (male, PA_4GPA_2)
“I didn’t find it that accurate in terms of flights of stairs because I have been doing a lot of moving house this last week and I did a lot of stair climbing and it wasn’t registering the amount of stairs I climbed and I thought that’s a lot, that was good and then some are long walks. It was registering walking and I wasn’t walking too maybe, I think just general movement it mistakes for walking or maybe that’s how it works” (male, G_4GPA_2)
“Only recently, as I say, I was moving a lot of stuff into my mum’s house and driving down so for the best part of the day I didn’t do that much walking and it sort of said I had done a lot of walking” (male, G_4GPA_2)
“If I went on a cycle or just to see ... I like to correlate things, so I’d see how close that was to the hand-held heart monitors on the gym equipment. Obviously, I don’t know which is going to be most accurate anyway. They were fairly consistent. I noticed that there were times when one was different to the other, slightly. Well, significantly, ten heartbeats” (male, GPA_6)
“I’m not too sure how accurate the pacing is. In the gym the last few days I’ve been alright. I’ve been back on the treadmills and it must be measuring the paces, I should imagine, by the movement in my arms. That was measuring a lot less. I suppose your walking stride is a lot less than your running stride. If I did a 6k in the gym, it was showing about 4k, so a little bit out” (male, GPA_6)

"I've just looked at it there. You see the calorific measure - I didn't really look at that much because I'm not quite sure how accurate that would be" (male, GPA_6)
"I'm doing 20k steps a day, although I'm not sure about this. If I go to the bathroom, get the towel and turn around it gives me three steps. You could keep doing that" (male, G_4GPA_2)
"I know you can register when you're doing a bike ride but it doesn't seem to then record anything for the bike ride, other than it seems to think I've been going up and down stairs a lot of times. That's how it seems to react to the bike ride, in terms of the syncing onto the device. When I came to see [the researcher], I got up, came here, went home on the bike and it told me I'd been up 20 flights of stairs. Well, I hadn't but I had been on the bike, so whether it recorded that" (male, G_4GPA_2)
"I think sometimes it's been quite generous with steps. Turning around to get the towel is giving me steps and some days it said, 'you've walked 'x' miles' and I thought, 'I can't have'. Some days it's told me I've walked eleven miles and I thought, 'have I walked eleven miles?'. Eleven miles is quite a … it's less to Leicester, isn't it? I don't feel I've done that. That might be walking round Beacon Hill, which is two to three miles, a couple of miles in the morning, then it's adding up the rest in bits and pieces, so I'm not sure it's accurate to that extent" (male, G_4GPA_2)
"I did notice that you can set them up to put in what you eat and the calorific intake. I thought there is perhaps room for a lot of inaccuracy there. I might be being unfair to the makers of this device but I thought that could be a lot of effort for not a very accurate return" (male, G_4GPA_2)
"(It's just done something, look.) It's given me steps for sitting and talking to you. Better rub that out" (male, G_4GPA_2)
"But the bit that wasn't accurate was the flights of stairs" (male, G_4GPA_2)
**Technology**
"Fine but for some reason the little film bit didn't go in, it went side wards, so [the researcher] has got that one, I think that one was a dud one" (female, GPA_6)
"That wasn't easy, actually. I would scan it and I'd think I'd put it in the right place on the phone and then it would go 'error' and I'd have to press it again. I'd put it where I thought was the same place and then it would still do it" (female, GPA_6)
"Sometimes it took quite a few times before it actually scanned. Towards the end, a couple of times there was no scan because it stopped working. It started again, so that's why there are probably some gaps at the end. I don't know what happened with it then, it wasn't in any particularly different place or doing anything different" (female, GPA_6)
"I'm a very patient sort of person. It doesn't really annoy me. It's just it would have been good if it did it right the first time but I wasn't quite sure where on the back of the phone you're supposed to place it in relation to the sensor? I think it was somewhere near the bottom but I never quite worked out where it was. I did it and then it would say 'error' and then I would press it and put it back again and then it might say 'error' again. You'd have to take it off and press it until you finally got it in the right place. I don't think I actually got it right the first time more than once or twice" (female, GPA_6)
"Most of the time it was like that. Then sometimes when you started it, it would just do nothing. So, you would take it off and there are two choices on it but you had to take it off in order to press the choice. When you pressed the choice, it couldn't sense, so that was a bit silly. I don't know why. I touched the correct app so it knew what I wanted to do. I scanned it but then after that it gave me a choice of two things and I didn't really need a choice of two things because I'd already indicated what I wanted to do. I wasn't quite sure about that. I can't remember the two choices now but one was the app symbol and there was another one next to it. I can't remember, not with my phone not here" (female, GPA_6)
"I did find it was quite difficult to sync the Fitbit because you pull it down and sometimes it didn't stay down. Sometimes you'd pull it down and nothing happened. You'd pull it down and it said, 'syncing is scheduled' so you didn't know whether you've got to stay there and be with it or not. That wasn't as straightforward as I thought it should be" (female, GPA_6)
"Sometimes, to sync it, you have to do it two or three times before it'll sync, whereas that it so immediate, it's just there" (female, PA_4GPA_2)

"I had a couple of issues… sometimes it's a bit difficult to link it to my phone, it just takes a bit of perseverance rather than everything happening on one flick of the screen, it might take two or three minutes just to catch on the Bluetooth. It's not a major issues, it's just a little niggle, that's probably a better word" (male, PA_4GPA_2)
"It's not been syncing very well for the last few days so I don't know what information there is there, I didn't follow it up with [the researcher]. Yes, you see there's days when there's nothing there and I don't know why" (female, GPA_6)
"And I might add that I had to manually sync and sometimes it would need two or three goes before it would actually sync, hence, hence, [the researcher] was aware that it's not syncing within five days at a time automatically" (male, PA_4GPA_2)
"The other thing is the syncing always took about two or three drawdowns to sync it. The sync was put in on a timing as well, so I didn't have to sync it myself all the time" (male, GPA_6)
"We were away last week and I was having trouble getting it to sync because the wifi on the campsite where we were was very poor" (male, G_4GPA_2)
"It was a bit temperamental. I don't know whether that was the Bluetooth on my phone or not. My phone was being temperamental anyway. I think it's got too much on it and it's struggling to cope with things, so it may have been the phone just struggling" (male, GPA_6)
"The first session, I guess the technology issues. To be fair [the researcher] did ask me before I even came the first time, can you download/install the app, does it do this, does it do that. You know, we thought we were covered. I guess that's just a learning curve for [the researcher] more than anything. I guess, it's not just the app. You have got to make sure the app is compatible with people's phones, and they are two different things aren't they. So that was a huge frustration" (female, PA_4GPA_2)
**DURABILITY**
**Knock/dropped off**
"It very nearly knocked off a couple of times after coming out of the shower whereas before I was patting it and thinking I've still got this thing on" (female, GPA_6)
"Fine, no problems, the only problem I had was with the second one, I actually caught it as I got into the car three days before it was supposed to come off" (female, PA_4GPA_2)
"It took a bit of getting used to because I kept knocking it, I knocked it off once" (female, G_4GPA_2)
"The second one a fortnight later, the probe dropped out" (male, G_4GPA_2)
"I think it was a fault with the manufacture. I put it back in and inserted it myself. Rebuilt it, put it back and it worked perfect. It could be a problem in the future with manufacture, I'm not sure, but it certainly had to be refitted" (male, G_4GPA_2)
"The fact that I had this last two weeks, I don't know how many days I had the monitor on for, when it was working it would have been a good three or four days. Then for some reason it stopped and I got another one on Monday evening. Plugged it into my arm and I went through the routine of cleaning it. My wife helps me to secure it in a place where I can access it easily and see what I'm doing. I stabbed myself, I have done this several times before. You have it for two weeks and just after the first week when I was working in very hot weather conditions and I was perspiring and it came away. Again, I got a replacement so I have done it a few times. On Monday night, I put it on nice and gently pulled it out and the whole thing came out, it didn't stick to my arm, because it was still there I went in again and just held it there hoping that it would stick and it did stick…" (male, PA_4GPA_2)
"It then began to tell me to replace the monitor and that's when I got in touch with [the researcher] again and said I would be here on Thursday and did she need me to put another one in. I asked her if it was an issue with my phone, or if I was doing something wrong" (male, PA_4GPA_2)
"I had an accident with one of them because I think I fitted it too high. I caught it on the roof of my car, getting into my car and it came off and I couldn't get it back on no matter how much I tried to put the thing back in the hole there was just no chance so I got in touch with [the researcher] and she organised another one" (male, G_4GPA_2)

"I did pull one out prematurely, just because it got caught on some clothing when I was changing, which was a bit annoying" (male, GPA_6)

"I had a couple of issues, the first one I was putting a climbing harness on and I pulled it off, caught it and pulled it off, so I had to come and get another one and then about two weeks ago, I was crawling about under the generator in a confined space and I caught it again and had to come and get another one. But apart from that, it's fine. I mean if it could be maybe a little bit smaller but the technology is strict on that or I could wear it on the inside maybe" (male, PA_4GPA_2)

"I did knock one off but I don't know that I knocked off it just like that. But it wasn't, because it's so easy to put another one in, it wasn't a massive problem" (male, G_4GPA_2)

"The only issue I had was it came out one day after showering a bit too vigorously. I tried to protect it but even so after … and it was coming to the end of its life, it just came out. We tried, very naively, to put it back in again, like this" (male, G_4GPA_2)

"It didn't bother me that much. Trouble is I did catch it a few times and I damaged a couple of the sensors, so I had to get a replacement" (male, GPA_6)

"I caught one on the car doorframe when I was getting something in and out of the car. The other one, I walked into a doorframe and caught it and it just bent the needle and it stopped working" (male, GPA_6)

"I lasted a day-and-a-half. I live in an old house, so I must have bashed my arm against the door and pulled it out, so I kept putting it back in. It kept falling off and it came off, so I had to get another one. Then I was a bit paranoid about it because I thought I was going to knock it and it was going to come off but it's been fine for the rest of the six-week trial...Unless it wasn't stuck on properly but it came off so, [the researcher] had to get me another one" (female, GPA_6)

"[the researcher] told me to put it here but the first one came out after a day because I caught it with my bra strap. I said to her, 'can I move it round?' because I caught it and it pulled the needle out. Maybe they didn't think of that when they designed it" (female, PA_4GPA_2)

"Yeah, when I was walking through doors and things I kept catching it. And I was worried it was going to sort of half come off or something" (female, PA_4GPA_2)

"With the first one, when it knocked off, a friend had just come around to look at the new kitten and I went, 'oh, it's just come off and I've got to put it back on'. She helped me with the plaster bit and helped me take it off as well. After that with the new one, I just did it all myself, so it was easy. It was fine" (female, GPA_6)

"The bit fell off, it fell off for around 2 days and I had to get [the researcher] to put another one on for me." (female, PA_4GPA_2)

"It got wet in the shower and it just fell off. I think I probably stayed in there for too long. She said it didn't matter too much so hopefully it hasn't jeopardised the report too much" (female, PA_4GPA_2)

"I didn't find it irritating at all. Just the odd time I caught it. Also, occasionally I would catch it in the door, walk past a door. It wasn't like….no problem" (male, G_4GPA_2)

"I was also quite worried about it because I actually had a bit of an incident with the first one because of how rainy it's been and obviously, if you are doing a run you get very wet and sweaty, the temaderm thing…it loosened and my t shirt caught it and pulled the whole thing off. After that I was quite intense about it. My daughter actually suggested why don't you pin t shirt sleeve out of the way and that just seemed to help. It survived and I felt much better after that" (female, GPA_6)

"If you get it slightly too far round, it becomes a problem in showers and if you're banging your way through doors, you could catch it. Just getting it round the back is quite helpful because then it is out of the way and you're less liable to catch it. Having said that, you have to have it in a slightly different place, so it's got to roam around a little bit. I think advice to push it round the back a bit might help, so it's then out of sight, out of mind. I couldn't say what anybody else would need, they're not difficult to use" (male, G_4GPA_2)

"I wanted to get ten out of ten from Maxine and then it started playing up so I gave up" (male, PA_4GPA_2)

Memory

"It's absolutely incredible. I think it's amazing. I just think it's annoying that it only lasts 14 days. I don't know whether that's because presumably it's got a chip in it, the chip maybe only has the capacity, or whether it's because of an infection risk, I don't know, that's annoying" (female, PA_4GPA_2)
"Glucose Monitor, yeah, I thought it might have a better memory, last a day or two but because I kept forgetting to, because I am so busy I just kept forgetting to scan it so there are gaps and also at night time I go to bed like 9, 10 o'clock at night time and then get up sort of 12 hours later after a really good sleep and there are huge gaps so" (male, G_4GPA_2)
"And, occasionally it didn't work. It didn't scan. It had an error or something and I had to do it again. And it only lasts for, is it 7 hours. So, you do lose a little bit of data" (female, PA_4GPA_2)
**PRIVACY**
"I forgot about it, I hate to think what you're going to find. No, it's not that bad. You will probably see that I'm constantly on my phone. Spend a lot of time of my phone, I'm like a teenager actually, terrible. I knew it was there, as soon as I switched the phone on" (female, GPA_6)
"I suppose it depends what they have on their phone. I wouldn't have thought so. I don't feel like it's big brother watching you at all. I didn't have a problem at all with any of it" (female, GPA_6)
"It was interesting that you could have all this stuff going on on your phone but it also is a bit spooky as well because people are watching your performance. I suppose that, in real life, can be quite scary. It is anyway - you don't know who's watching you at any point" (female, GPA_6)
"It's amazing the way it picks up what you're doing. How does it know I'm doing an outdoor bike ride?" (female, GPA_6)
"When I am away I use Google Maps as well and I have the tracking on. I look at that and I know it will tell me how I've been travelling, if I've been walking, on Google Maps. It's all a bit spooky because it seems to have a record of everywhere I've been for the past eight years, or something. I don't think a lot of people know that's there, that the record is there" (male, GPA_6)
"I think if you've got nothing to hide, you're not bothered. It's a bit like identity cards. I should imagine some people, when they find out, might find it a bit challenging. I do show it to people and they don't know. Sometimes they don't have the tracking on but I think they do find it quite alarming when they suddenly see it. They don't realise it's on Google Maps. I forget what the actual thing's called, I know how to find it though" (male, GPA_6)
"I'm not bothered about monitoring. Privacy issues don't particularly affect me. If you haven't done anything wrong, you're not going to get into trouble. I think the fact that she noticed that I hadn't synced just alerted me to the fact that they were looking at my records but it didn't bother me at all. I think there's far more subversive monitoring goes on through Wi-Fi at home. Down to the fact that people can detect where you are in the house by the changes in your Wi-Fi fields. It's nothing like that, so I wasn't particularly bothered about that at all. The things that Google monitor you on are probably far worse. You don't know about half of them or a lot of people don't" (male, GPA_6)
"No doubt, there will be people talking about privacy. That's fine, deal with that, don't get involved if you know what I mean. People are happy and friends of mine are a lot more active than I am. You see them with the latest bike ride or the latest run or whatever. And they are happy to do that" (male, G_4GPA_2)
"if I give you this example... 'Your glucose is low, you need to do something about it'. That for me has got to be a good thing. And also, there is value in the big data that will produce. We all worry about big data, because it has been used in bad ways as well as good ways. Because if you have big medical data like that, and Anonymise and all those things. Google for instance is the best example of that. People go, oh, they get reviews of things. Well they don't jump out the air, that's people. Google understand big data, well they didn't invent it but they are there at the forefront of it. They understand it has a value bigger than the individuals. Big data tells you something" (male, G_4GPA_2)
"What I'm saying is if you trust somebody it's fine but then you start thinking, 'who else is getting in on my phone?' and I suppose that's worrying but I think most of us have those worries anyway" (female, GPA_6)

“But I am of the opinion that sometimes that’s worth what you get back. I always say about Google, and people talk about Google. You get a free Sat Nav, you get Facebook for nothing, you get Messenger for nothing. You have a phone, you only pay for the phone and the data. All that technology is for nothing. They don’t charge you for Android. You get these massively complicated bits of software that cost millions of pounds to develop. What you give for that, you give a little bit of privacy, but actually it’s not that intrusive. They aren’t interested, not on an individual basis. They are interested in why all of us are there, and that big data thing” (male, G_4GPA_2)
**PREFERENCE**
**Physical activity features of the FB**
“I think the only thing I would have been interested in is the sleep bit but that was switched off. I think it is on here but it wasn’t activated on there. I was thinking how many hours I actually do sleep because my daughter was telling me she has had this much of REM and this much of that, and this much of proper sleep. I know it can do it, but it wasn’t set up to do it” (female, PA_4GPA_2)
“I tried to, I mean I had a look at the heart rate thing. I did try to use the sleep thing actually, that was quite interesting” (female, GPA_6)
“So, I would say the heart rate data for me has been more interesting. And I have looked at that more than any of the other data. I wasn’t really, I didn’t really pay any attention to the steps a huge amount” (female, PA_4GPA_2)
“I found the step goal quite easy, I met that most days” (female, GPA_6)
“The calorie goal was very hard, very few days was I meeting that” (female, GPA_6)
“I use an activity tracker anyway so I track my steps if nothing else, it’s my steps which is an indicator so I don’t really look at distances or play with the GPS I mean” (male, PA_4GPA_2)
“Before putting this on I wasn’t bothered about monitoring the heart rate and I just thought if I was a serious athlete or sick there would be reasons, otherwise I just don’t think the average person needs to suddenly monitor the resting heart rate but I was doing it because occasionally I feel breathless at rest which is a little bit concerning for me so I was checking it and generally there was no relation” (male, PA_4GPA_2)
“In a way, I’m now a new convert to wanting a heart rate monitor because I think, if I’m unwell in any way at least I know it’s there, I can look at a glance and I know that one of the things may be a really high heart rate” (male, PA_4GPA_2)
“I’ve learned is that I value being able to read my heart rate and being able to read my steps which I already had anyway so, but I probably would have, I haven't been accessing any of the other activities on her because I’ve not really not doing much and if they use gym equipment it’s too irregular for me to then monitor, it just wouldn’t work, so I suppose that I’ve just learned that those two elements are good” (male, PA_4GPA_2)
“Yeah, I think I may look for a device similar. Obviously not the glucose device but something that, a wearable tech that will tell me how many miles I’ve walked or how many steps, something that way” (male, G_4GPA_2)
“I haven’t liked the Fitbit at all, really haven’t enjoyed using that. I think because I do so much running I feel a little bit resentful of my wrist watch telling me to get up and do some more walking. You know the two hundred and fifty steps, nine hours a day target... I didn’t really enjoy the Fitbit, I wouldn’t go and buy one now, having had that experience of one” (female, GPA_6)
“The steps per hour bit I wasn’t that interested in really. Calories burnt I felt quite good and the distance covered in kilometres yeah” (male, G_4GPA_2)
“I’d quite often look at my heartbeat because that was the most interesting thing on there” (male, GPA_6)
“I look more at the steps. That’s what I had on the big screen and it was the steps I focussed on, nothing else. I occasionally glanced at the calories and it was always about 3300-3600 I was consuming” (male, G_4GPA_2)
“The heart rate, I noticed I was doing that. I noticed my resting rate went up, actually, during the End to End, which I suppose it would do but it had been lower prior to that. So, I kind of wanted that too” (male, G_4GPA_2)

“Probably the steps and the active time more than anything” (male, GPA_6)
“I seemed to have always hit the calorie count. I don’t know whether that was I found the settings perhaps a bit low or I seemed to be well over the calorie count or not. I didn’t have any problem hitting that at all” (male, GPA_6)
“I quite like the heart monitor if you’re doing any exercise. I used to go on a cross trainer quite a bit but it would be nice to have something that monitored your heart rate and kept track of where you were with that” (male, GPA_6)
“Yes, well I didn’t understand why it only wanted me to do them between 9 in the morning and 6 in the afternoon. 250 steps are quite easy to do, isn’t it? It’s no distance at all really. I don’t lead a very structured life every day is different and it was during the summer when the nights were light so I would often go gardening until 9 at night and it didn’t want to know about that. It was only interested in stuff before 6 o’clock. I did feel a bit cheated” (female, G_4GPA_2)
“The only one I didn’t really do was the calories, I just completely ignored the calories” (female, GPA_6)
“The pulse was quite interesting and how many calories you burn was quite interesting as well” (female, GPA_6)
“I don’t understand the ‘relax’ bit. Generally, it’s just the steps” (female, GPA_6)
“I liked the steps and the other thing I used was the active minutes was quite interesting I thought, that one I used quite a lot. So, the steps and the active minutes I thought were quite useful” (female, GPA_6)
“The Fitbit, I was interested in sleep and obviously steps but much more than that, I didn't really look at, partly because I didn't understand it and partly time and lack of interest really...Calories didn't interest me really, I don't know why, I didn't trust it really” (female, GPA_6)
“Basically, I just use it for the steps and used it to see how many minutes are higher, it’s not very much. I haven’t used all these extra bits and pieces and this sort of hours in the day, to get an idea” (female, G_4GPA_2)
“But, that is a prompt, it definitely is a prompt to actually look at it and say okay and I like the fact that it recorded cycling as well” (female, GPA_6)
“It was alright, that was probably one of the least useful goals for me because my activity doesn’t really work like that. I might sit around for a bit and then do a Zumba class, which would be loads of steps. Also, I commute, so that does often mean more than an hour in the car at a time. It was a nice reminder that you’d been sitting around for a bit. Why not wander about?” (female, GPA_6)
“I believe I got some merits, something gimmicky. I hated these little comments that said, ‘let’s get going’ and silly, childish motivational comments. I hated them. I thought, ‘I do not need these’. They put me off really” (female, GPA_6)
“the fact that it buzzes when you do steps, I'd have guessed that didn't really interest me. The fact that it does tell you to get up and walk, I think that's very good with certainly doing a desk job a lot of the time, I think that is quite a good thing to prompt you so yes, I did like that aspect of it...I think it is useful, yes, we spend so much time on the computer these days that generally I think it's a good aspect of the Fitbit to get you doing that” (female, GPA_6)
“While I was aware of it, it probably didn’t vibrate hard enough” (male, G_4GPA_2)
“When I got the full function of it, it was a bit disappointing really, the battery thing was good and the relaxed thing I didn’t really get that at all. And the vibrating was irritating. Yeah, that was reminding oh you’ve got to do this so I don’t know” (male, G_4GPA_2)
“When I did for the first time I thought, what is going off on my arm, it’s like fireworks and I thought it wasn’t going to stop and it just kept doing it. That was quite funny to actually see that” (female, GPA_6)
“You don’t want to make it something that is always on your case. Computers are on your case often enough anyway without wearing one. I don’t mind it rewarding me but I don’t want it telling me off or having to be fed with data about what I eat” (male, G_4GPA_2)

"I think it is a good thing in a way as it does prompt you to do things. I may treat myself to one, I'll see how it goes" (female, PA_4GPA_2)

"Not really, no, I'd just ignore it but every couple of hours might have been better. I'm not sure what the idea between every hour, whether it's good to have exercise every hour or what. It might be more achievable for people if it was perhaps every couple of hours" (male, GPA_6)

"I mean I did look at my heart rate. When I would be out walking I would be like, Oh I am in Fat-burn you know. What was I doing the other day, I wasn't doing anything but I was stressed and it said i was in Fatburn, and i wasn't bloody doing anything" (female, PA_4GPA_2)

"I thought I don't wasn't to get, I am obsessive enough without obsessing. I am tempted to buy one that monitors your heart rate and things actually, that's quite useful" (female, GPA_6)

Non-physical activity features of the FB

"But what else did I try? Yeah, I didn't pay too much attention about weight actually, I didn't really, I didn't get round to that actually" (female, GPA_6)

"If it could have done cycling as well it would have been brilliant, yes... I suppose swimming as well. If you got your exercise that way, it would be good as well because I did do some swimming in the sea... It would be good to have had one that would work for other types of exercise. For people that can't do weight-bearing exercise, that do swimming or cycling. That would be motivational to them to have something because if it doesn't register the types of exercise you're doing, you wouldn't use it. You'd be disheartened and give up" (female, GPA_6)

"I couldn't quite work out what these exercises where. You know when you.... I presume... I don't know what you are meant to do with that. Do you sort of just hold it as you are doing your exercise or? I think I used the stopwatch a couple of times timing stuff. And I did the relaxing thing" (female, PA_4GPA_2)

"I just didn't bother in the end. I could have done I suppose, but I used to be, he gave me I think I was on his fitness pal, My Fitness Pal app, that was much better than this one… It was just, I think their database must be a lot better. So certainly, the bar code scanner and then you can set, you can even put in recipes and you would work out your calories but the scanner was brilliant actually. You just need to scan everything and it would immediately tell you. You could even choose, even if it didn't scan you could say muesli and then it would give you like Sainsburys, Waitrose, whatever. So, it seemed a lot more easy to use and comprehensive than this one" (female, GPA_6)

"I didn't fill in any of the food diaries. Too much trouble, probably too embarrassing as well. It's hard to remember because it's one of those things you just look at on the app" (male, GPA_6)

"The other thing, I never used the relax thing … well I did, I used it about twice and thought it was a bit of a fad… the relaxing thing obviously you get all these sparkles coming up by doing certain behaviour. I found that a bit silly, really, which probably said more about me than the app" (male, G_4GPA_2)

"I started off doing the water but I do that on Weight Watchers, so I didn't do that. I didn't do the eating bit either because I do it on the Weight Watchers site" (female, GPA_6)

But you couldn't even join friends on this one. I tried to think friends up but I suppose because it's not my account it wouldn't let me link with anybody" (female, PA_4GPA_2)

"Once I start with some of these things I can get a bit sucked in. I didn't really want to go down the Fitbit and the Strava and Movescount, which is the one I use for my particular running watch. I'm on a running website called Fetch Everyone, which a running group website, adding the Fitbit on top of these things would just be a waste of time. It wouldn't really give me any extra information that I could do anything with or that I would use" (female, GPA_6)

"You know, it's just lack of time in some ways like, recording how much you drink. If they had an easier way of doing it, I don't know, I might have logged that" (female, GPA_6)

“The only thing was that sometimes I’d do a reading and then I’d think oh, I’ll put a note in but then I think once you’ve sort of gone and done something else, you can’t go in and do a note afterwards later on when you remember, unless I just don’t know how to do that. Yeah, I don’t think I could go in now and put a note in” (female, G_4GPA_2)
**Features of the FL**
“It gives you those daily reports which are helpful. And I thought it was helpful that you could add in a note when you had eaten something. So, you could then align, I mean in the end you didn’t really need to because you could see the patterns for yourself, but to start with that’s useful that you could put in when you had eaten something. Or put a note in of some sort which is helpful” (female, PA_4GPA_2)
“Oh yeah, the other thing that I found useful, and I guess it’s probably even more specific to this research. It allows you to look at the data over 7 days or 14 days… I guess that it doesn’t give you much control over that though. So, what I couldn’t do, I couldn’t look at one portion of 7 days and then a different portion of 7 days. I could only look at 7 and 14 and then is it 28 or 30 or something. So, I guess I would want to be able to portion up the data a bit more...” (female, PA_4GPA_2)
“I looked at some of the averages and things like that. So, if I used it in that way, I could start monitoring weight loss and improved cardio and all that” (female, GPA_6)
“No, it was estimated A1c, I think, so that doesn’t really give you much information about that at all… Yes, the other thing is you’ve got this band that you’re trying to keep in but it would be nice to know what a normal sort of pattern is for going in and out of the band as well. That’s about the only things, really, I could think about it” (male, GPA_6)
“I did like that app on the phone that you could see the graphs or compare weeks to see how not active I'd been or active and what counted towards that so, yes, I found those bits quite interesting on that… Being a numbers person, I quite like graphs, bar charts and things so I thought that was quite interesting” (female, GPA_6)
“I like the traffic light idea. It’s a bit like the food labels. Immediately you know if it’s red it’s too low, green you’re okay and amber you’re ‘ham sandwich’, whereas you don’t have that with the Fitbit app” (female, PA_4GPA_2)
“And, I know this sounds really petty now, but the blobs on the graph as well, they are so big you can’t actually see the line most of the time, which annoys me… And then it shows you the black line, but then you have got the green amber, I am assuming there is a red, I never got a red. Especially when at the beginning I was taking lots and lots of readings, it almost obliterates the line because the blobs are too big” (female, PA_4GPA_2)
“I think patients want to be in control of their information, their health. I feel that that’s kind of, it’s close, but not quite close enough. It’s kind of tantalising. And I can see it, but I can’t get hold of it. And so, it will plot it, and I can see that it was more than 9, but I can’t see what. Is it 9.1, 9.2, 9.3? Because it’s so small and you can’t see. Does it matter? I don’t know. But it just frustrated me that I couldn’t tell” (female, PA_4GPA_2)
"Technology while I was in the nursing was just monitoring rather than predicting. This is predicting which is something that is I haven’t really done in the past. Using this technology to predict, it didn’t take into account my own perception of me" (male, G_4GPA_2)
**FB versus FL**
“I wasn’t bowled over by it. I was fascinated by the technology but as I say, I found the information from the glucoses much more interesting” (female, GPA_6)
“I have actually enjoyed the glucose monitor results, looking at the graphs and charts more than the Fitbit” (female, GPA_6)
“Yes, probably the glucose is more interesting for me. Whereas there are other ways to know your activity, aren't there, and things like that whereas that was very specific to this study for me so, yes, as a preference that was more interesting” (female, GPA_6)
“I think, if anything, I prioritised the blood sugar level, just because it’s more interesting and perhaps I didn’t know what it was going to do” (male, GPA_6)

"I could have delved a lot more deeply into the Fitbit but I was more interested in the app and the glucose levels… I've never monitored my glucose before and I was fascinated by the whole think. I was amazed how the device worked and all sorts of things about it. How it keeps scoring up the last seven hours' data and how it transferred to the phone. I've never used the proximity device before, I don't use Samsung Pay or Apple Pay or anything, so the whole thing was technologically interesting to me" (male, G_4GPA_2)
"I'd say I probably focussed more on one than the other. The Fitbit, I probably switched off a bit from that, with having the glucose monitor on because the glucose monitor, you've got to do it every seven hours, so it almost pushes you to look at it rather than the Fitbit, you can just put it on and forget about it. So, I probably focussed more on the glucose monitor, when I had them both on. I still did look at the Fitbit but I didn't find it any problem having the two lots of data" (male, GPA_6)
**COST**
"I imagine the cost of them is not cheap" (female, GPA_6)
"I know they're expensive so it's not available for everybody to do that… I think the fact that it's not on the National Health means it will be out of reach of most of the people I know at any rate, which is sad… It's amazing technology. Worth every penny, tell the National Health" (female, GPA_6)
"It is a lot. I am sure they will come down as people use them more and more, but, and they only last two weeks, so you know it is a lot of money" (female, PA_4GPA_2)
"And they're bloody expensive. Dear to buy… Well it's because of the cost, they are quite expensive but like I say if I had an implant. If there was something injected in your arm that would last 12 months then that would be very interesting yeah. I would be up for that" (male, G_4GPA_2)
"I think if you said to someone you are going to have to do this till you die - somebody my age has just been diagnosed with Type 2 Diabetes or younger, and you certainly might do that for 60 years, and it's twice a month for 60 years. That might be a harder sell" (male, G_4GPA_2)
