# Supplementary material for: Examining the Use of Glucose and Physical Activity Self-Monitoring Technologies in Individuals at Moderate to High Risk of Developing Type 2 Diabetes: Randomized Trial
Source: JMIR Mhealth Uhealth. 2019 Oct 28;7(10):e14195. doi: 10.2196/14195 (PMC6913728; doi:10.2196/14195)
Supplement: Multimedia Appendix 4 [file mhealth_v7i10e14195_app4.pdf]

Freestyle Libre – data capture (%)/missing data in min

Table. An outline of data capture from the Freestyle Libre stratified by group allocation, reported as mean±SD

	**Week 1**	**Week 2**	**Week 3**	**Week 4**	**Week 5**	**Week 6**
**Total data capture (%)**						
Group 1: G_4GPA_2	87.6±2.7	87.4±15.7	87.2±16.5	82.0±21.5	85.8±14.7	83.1±17.5
Group 2: PA_4GPA_2					80.2±11.7	73.2±28.6
Group 3: GPA_6	86.7±4.8	93.7±4.5	91.9±6.5	91.1±9.1	89.0±11.6	89.6±10.8
**Amount of missing data (minutes/day)**						
Group 1: G_4GPA_2	27.4±6.5	27.1±31.7	26.8±33.6	37.7±44.0	29.8±30.0	35.1±35.7
Group 2: PA_4GPA_2					41.6±23.3	55.3±58.5
Group 3: GPA_6	28.0±9.8	13.0±9.2	17.1±13.0	18.8±18.6	22.7±23.8	21.6±22.1
