# Supplementary material for: Examining the Use of Glucose and Physical Activity Self-Monitoring Technologies in Individuals at Moderate to High Risk of Developing Type 2 Diabetes: Randomized Trial
Source: JMIR Mhealth Uhealth. 2019 Oct 28;7(10):e14195. doi: 10.2196/14195 (PMC6913728; doi:10.2196/14195)
Supplement: Multimedia Appendix 5 [file mhealth_v7i10e14195_app5.pdf]

Physical activity – steps taken, number of active minutes, floors and reminders to move

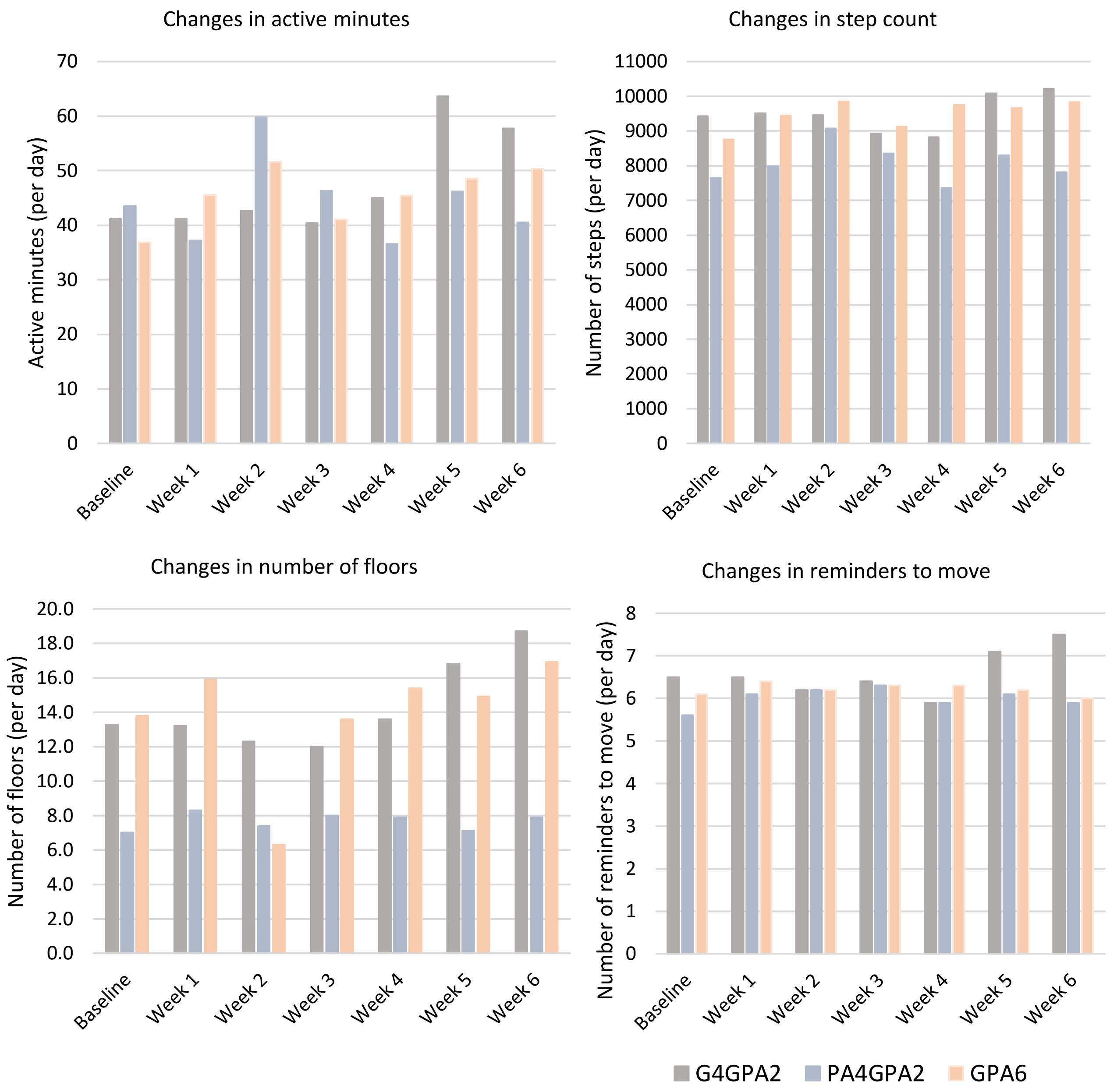

Figure. An overview of number of active minutes, step count, number of floors and reminders to move recorded by the Fitbit, adjusted for wear time.

Physical activity feedback – number of prompts and number of steps taken after a prompt

Only having access to physical activity feedback during the final two weeks of the intervention, participants in G_4GPA_2 achieved 250 steps before the hour in 24.2±17.5% and 26.3±16.6% of events where a prompt occurred, and participants averaged 82±55 and 82±51 steps after being prompted (in the 10-minute window), respectively.

After being prompted to move, participants in group PA_4GPA_2 reached 250 steps before the hour on 20.7% (week 1), 19.3% (week 2), 20.2% (week 3), 21% (week 4), 16.5% (week 5) and 22.5% (week 6) of events. After being prompted, participants averaged 64±38, 67±45, 57±26, 59±42, 45±20 and 69±43 steps (in the 10-minute window), respectively.

Similarly, participants in GPA_6 achieving 250 steps before the hour after a prompt on 24.9% (week 1), 27.8% (week 2), 28.2% (week 3), 24.6% (week 4), 28.2% (week 5) and 20.3% (week 6) of cases of prompts. Participants then averaged 99±91, 102±149, 60±36, 93±126, 78±88 and 73±58 steps after being prompted (in the 10-minute window), respectively.
