# Supplementary material for: Examining the Use of Glucose and Physical Activity Self-Monitoring Technologies in Individuals at Moderate to High Risk of Developing Type 2 Diabetes: Randomized Trial
Source: JMIR Mhealth Uhealth. 2019 Oct 28;7(10):e14195. doi: 10.2196/14195 (PMC6913728; doi:10.2196/14195)
Supplement: Multimedia Appendix 6 [file mhealth_v7i10e14195_app6.pdf]

Interstitial glucose – average level (mmol/L)

Table. An outline of interstitial glucose levels recorded over the six weeks stratified by group allocation, reported as mean±SD.

	**Week 1**	**Week 2**	**Week 3**	**Week 4**	**Week 5**	**Week 6**
Group 1: G_4GPA_2	5.7±0.9	5.6±0.9	5.6±0.7	5.5±0.6	5.5±0.4	5.4±0.5
Group 2: PA_4GPA_2	[redacted]				5.6±0.5	5.5±0.5
Group 3: GPA_6	5.5±0.4	5.5±0.5	5.5±0.7	5.5±0.5	5.5±0.7	5.5±0.6

Glucose feedback – time in range / rate of change

In an effort to display what information participants were presented via the LibreLink app, glucose characteristics are presented in the below figures.

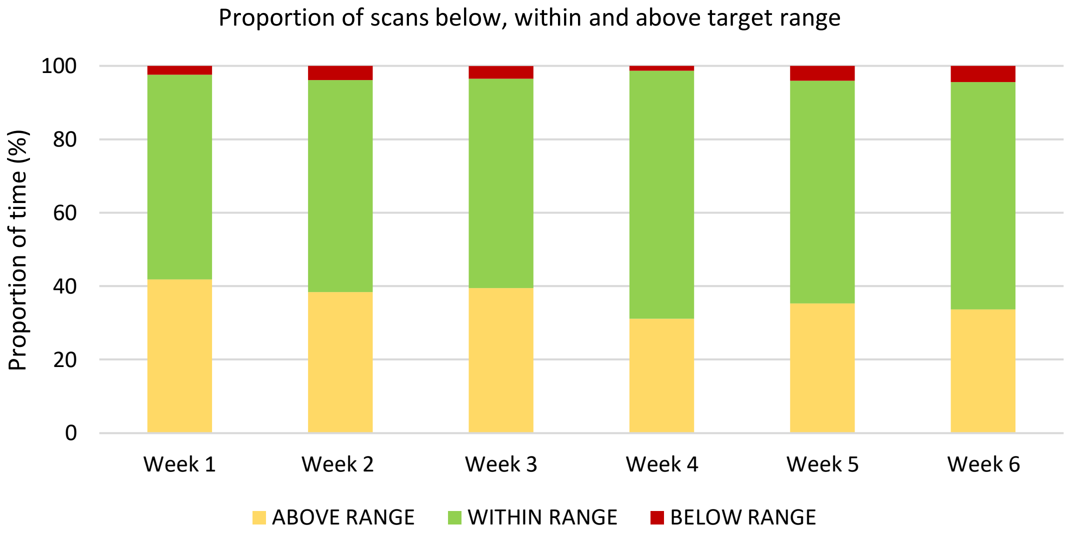

Figure. An overview of the proportion of scans recorded as being within, below or above target range. *all participants except weeks 1 to 4 during which there is no data for PA_4GPA_2 participants.

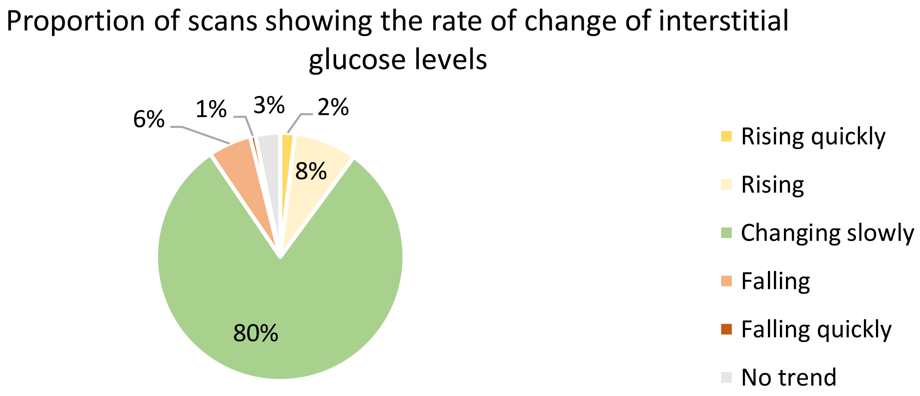

Figure. The proportion of scans that showed participants were experiencing a ‘changing slowly’, ‘rising’, ‘falling’ (and so on) direction of change arrow via the Freestyle Libre application.
