# Supplementary material for: Examining the Use of Glucose and Physical Activity Self-Monitoring Technologies in Individuals at Moderate to High Risk of Developing Type 2 Diabetes: Randomized Trial
Source: JMIR Mhealth Uhealth. 2019 Oct 28;7(10):e14195. doi: 10.2196/14195 (PMC6913728; doi:10.2196/14195)
Supplement: Multimedia Appendix 7 [file mhealth_v7i10e14195_app7.pdf]

Box. All quotes pertinent to the wider study.

**POSITIVE EXPERIENCE**
“I've really enjoyed it, I really hope that the results make a difference, certainly with the glucose monitor; I really hope that people are able to take notice of the results. I hope that people have had similar experiences to the ones I've had and that people that are in charge of letting these devices go on the NHS are able to take note, yes” (female, GPA_6)
“I really enjoyed it. I really learned a lot from it and I’m keen to hear what the outputs are” (male, G_4GPA_2)
“But as far as the research is, I don’t think you could fault it. No. I thought it was simple, there was nothing more I, it was absolutely the bee’s knees. I really do” (female, GPA_6)
“I’ve met [the researcher] and she’s been totally professional and competent. It was clear what the study was about, the protocols were clear and I was treated, each time I met her, with respect and humour. I enjoyed doing the physical jerks at the beginning. I wouldn’t change anything, particularly” (male, G_4GPA_2)
“No, I think it was run as well as it could be. Appointment timing and everything were great especially with working and I managed to fit my appointments in around my programme at work. So, no, it was fine” (male, PA_4GPA_2)
“I enjoyed doing it, I just enjoy doing the whole thing” (female, GPA_6)
“I don’t think there is anything else I can say about it, apart from that it was interesting and I enjoyed it” (female, PA_4GPA_2)
“It was quite a fascinating six weeks” (female, GPA_6)
“I just enjoyed it, which I expected” (male, G_4GPA_2)
"Just meandering about, it is still moving. It was enjoyable to the degree that I wanted to do it rather than it was a pain or it was drudgery, it wasn’t I did enjoy it" (male, G_4GPA_2)
**WANTING FULL ACCESS TO FEEDBACK**
“I was lucky I was in group three” (female, GPA_6)
“Yes, I was really pleased that I was in the six-week group because I thought that will give me a lot to go on, whereas two weeks is kind of neither here nor there. I thought six weeks is a reasonable amount of time to assess things. I thought that was good for me and it led me to sort of draw some conclusions about glucose and how I was using it” (female, GPA_6)
“The first week, I just had to Fitbit and then I couldn’t see it. That drove me mad the first week because I wasn’t allowed to see it. That was a tough one that week, so it was nice that I wasn’t on the group where you weren’t allowed to see it at all.” (female, GPA_6)
“I would have been really annoyed [if allocated to another study group]. It’s been quite nice being able to look at it all” (female, GPA_6)
“No because it gave me access, I was quite pleased to be in the group that had access to all that information” (female, GPA_6)
“I found the first week very frustrating when you had this new bit of technology and there was a bit of tape round it. Having said I'm not really into technology, that's just my nature, don't give me something and then I can't see it; if I've got to wear it, I want to know what it is. That was frustrating so I was quite pleased to be in the group that had access to the information” (female, GPA_6)
“Yes, the first two weeks I couldn’t see it. I didn’t like that and I never cheated once” (female, PA_4GPA_2)
“I had this in for four weeks where I couldn’t see it and I have to say I preferred it when I could because you always think, ‘I wonder what it’s doing?’. I do like to know what’s what” (female, PA_4GPA_2)

“I’d like to have had more access to it earlier. I presume this must be a dual-purpose thing – the diabetes side of it was why I was on my glucose monitor and also how people interact with the technology. I would like to have had that access from the start because I’m quite sad that it’s finishing” (female, PA$_4$GPA$_2$)
“That, in fact, the first week, the first week I had here, [the researcher] put a little black sticker on it, so I couldn’t see anything. When I got home I put another one back on for a week. Somebody said to me, ‘You really ought to look at it’... and I thought, well it was on there for a reason. It was probably about five or six days. It wasn’t probably quite as long as a week. I didn’t want to know what it was telling me. Ignorance is bliss. That’s how I look at it. And then I thought, well they are obviously doing this for a reason” (female, GPA$_6$)
“And I was really annoyed that I didn’t get randomised to the arm that I wanted. Because I wanted the blood glucose measurements for the whole six weeks. And I only got it for two weeks. But anyway, I got it so” (female, PA$_4$GPA$_2$)
“I guess, yes, lasting longer that two weeks would be good” (female, PA$_4$GPA$_2$)
“So yeah, I would have liked, I guess I would have liked more than two weeks. That was only, Thursday to Thursday, where there two weekends in that?” (female, PA$_4$GPA$_2$)
**STUDY DURATION TOO SHORT**
“It is difficult to do that over six weeks. Perhaps over several months you might be able to pin it down to what you eat and when you eat it. Perhaps keeping a food and exercise diary and linking them together, but we weren’t asked to do that” (female, GPA$_6$)
“It would have been interesting to see it over a longer period of time, say three months. I’ve only been able to see it for a month out of seven weeks and I would like to have got that access for that because it would have been interesting to see if the steps went up or I lost weight. For me, it’s quite short. It’s probably fine for your purposes but for my benefit, I would have liked it to have been longer” (female, PA$_4$GPA$_2$)
“So, one of the other things where, the other thing, and I don’t know whether this is, and I haven’t had enough time to play about with it. I really want to have it longer, which is really frustrating. I don’t know whether it’s what I eat that it seems as though my blood sugar spikes at breakfast then doesn’t spike as much at lunch and doesn’t spike as much at dinner. And I don’t know whether that’s because of what my diet, what I have, or whether that’s just because that’s what my body does at those times. And I haven’t had enough time to work that out yet. I have orange juice for breakfast and I know that will spike it. But I would like more time to play with it really” (female, PA$_4$GPA$_2$)
“To have only had that for two weeks … I would have loved to have seen how it panned out over a longer period” (female, PA$_4$GPA$_2$)
“It has been really eye-opening it has. I am glad I did it. I am just annoyed I haven’t got it for longer” (female, PA$_4$GPA$_2$)
“I think, I don’t think the amount of time that you gave people is enough to really see the differences” (female, PA$_4$GPA$_2$)
“Six weeks isn’t a huge amount of time, so if I wore it for a year I don’t know if I’d still be thinking that” (female, GPA$_6$)
“It’s quite a short study, so almost whatever you did in that time shouldn’t be having a massive effect if you’re not already ill, which I know you check at the beginning, that you’re not already diabetic” (female, GPA$_6$)
“Yeah. I don’t know, maybe it is. But I guess that’s just a personal thing because of what I was going through, and I feel that you haven’t seen a representative me, if that makes sense. Maybe it is long enough. But I didn’t feel that I had long enough because of what I was going through, for you to see a representative me” (female, PA$_4$GPA$_2$)
“No, because it was quite a short amount of time. I think if I had one and if it was, say, three months or something, yes, I might have got more out of it, in the sense of I’d have set more challenging goals.” (female, GPA$_6$)
“I think if I had one for longer I would put it in different places as well. I would want to move it I don’t know. My abdomen, back of my shoulder, my leg or somewhere different so it’s not so obvious” (female, PA$_4$GPA$_2$)
**ISSUES WITH ELIGIBILITY CRITERIA**

Who

"I think probably more so for people that are at risk, perhaps if people that, like my father, who was told a couple of years ago that he was at risk, perhaps if you had one of these and you were able to monitor your levels for a while then it might make a difference to changing your lifestyle" (female, GPA_6)
"I think it is probably good if you are pre-diabetic or you are at risk I think they are a brilliant idea as it doesn't interfere" (female, GPA_6)
"It's quite good. I imagine it's very useful if you actually do have diabetes, to manage it" (male, GPA_6)
"To introduce it to people with diabetes. Demonstrate to them how their body was handling, or not handling the glucose that they may or may not have. It would be a good tool for that" (male, G_4GPA_2)
"Yes. Not necessarily for non-diabetes but for those that need the insulin. They could see, even if they were trained as nurses or doctors, they would be able to see the effect that the insulin, or lack of is having, in front of them rather than joining dots or a graph" (male, G_4GPA_2)
"They do these Desmond courses, where people go along when they get diagnosed, to manage it. You could maybe do a similar instruction with a phone or there might be some other implement. They don't have to have a phone, they could have an ipad or a tablet. It's just a thought" (female, PA_4GPA_2)
"Yes, I did discuss it with her quickly because she said she'd been trying to get hold of the little probes for quite a long time. She said they won't supply them on the Health Service and she'd had a great amount of difficulty getting them. She said, 'It's typical you go on a study for the university and you don't even need one and you get them shoved in your arm straight away' and she'd been wanting to use them for monitoring purposes" (male, GPA_6)
"I think you have to pay for it if you're a diabetic. You've got to self-fund for that but wouldn't it be great if all the diabetics could have that on the National Health Service. It would really give them such an accurate measurement" (female, GPA_6)
"I think if you are diabetic or you are looking at whether you might need the insulin or there are things that you can do that will change that then having all that information means you're not relying on going to the doctor's all the time to check stuff, it's very self-monitoring and I think it would help, well it did help me, it was interesting and plus if you are under the doctor or anything like that, it would be very useful for them as well" (female, GPA_6)
"Well, I just think it's useful, it helps people and if you were diabetic and you could use that, why wouldn't you?" (female, GPA_6)
"And, again, with it going low as well, I mean not for people who are at risk but for people that are diabetic definitely to know that oh ey up, I need to eat something. So, yes, I can't see any wrong with it at all, I think it's fantastic" (female, GPA_6)
"I think it would be good for people that do have diabetes, to constantly have that tracker on them to know when they are going down. It would be helpful but I suppose people with diabetes kind of know when their sugars are going down. If they had a warning before they actually got to that point it would be good" (female, PA_4GPA_2)
"Right now, I think it's absolutely amazing technology. I think it's incredible and I know now that they are on the drugs list aren't they now for Type 1 Diabetes, and they absolutely should be. They are just astounding. I know they are not cheap but... Yeah, there is an argument there isn't there, about whether, should people who, like me.... well clearly, I have got pre-Diabetes. You can see that from the data. Should we be given that sort of thing so that we can look after our blood sugar better" (female, PA_4GPA_2)
"Someone who monitors daily could probably benefit from a sensor like this rather than pricking your finger daily" (male, PA_4GPA_2)
"Yet, these machines enable my wife to get a little bit of that confidence and know-how. She can use this and she knows whether to eat or not eat or take more insulin or not. It's instant and it's really helped her" (male, G_4GPA_2)
"And also, you could tell people easy ways they could do it. You know at dinner time when you want to go, walk a different way you know. Instead of going to that shop there, walk a different way. Take the stairs, take the steps. Park the car at the other end

of the car park, things like that. They are all a bit of an abstract thought. With this, you would be able to say actually, and there's the benefit. The benefit is the amount of steps you done. Oh look, because you did that extra 1,000 steps today your blood sugar level's a little lower. And I really think that will work" (male, G_4GPA_2)
"But I can see for people who do have Diabetes, in fact there is a lad in one of the offices I work in, he has Type 1 Diabetes, he is from the Asian community. And he was really interested in it because he knew about these sensors, he is aware of those... He was really interested and he said, well actually, yeah, particular part 2, Type 2, because I could see what would be really useful for people. Because, for one thing, it's a motivator. If you say to somebody, you have got Type 2 Diabetes. You can either take medication for the rest of your life or you might be able to drive it down just by being more active." (male, G_4GPA_2)
**Android**
"I had a compatible phone as well, which I don't think is a particularly good selection criteria. It was a Samsung S6 and it worked with the apps" (male, GPA_6)
"I don't know how biased the sample was towards people with compatible phones, for a start. I've got two phones, my own phone, which is a Samsung S6 and I've got a work phone, which is a pure Google Nexus one and she said that wasn't compatible. If I didn't have my own phone and I only had my work one, that would have knocked me out of the study a bit, so I think that the sample wasn't exactly random, on that basis, which I suppose you would be looking for" (male, GPA_6)
"My friend, who is a Sister on the respiratory ward at Glenfield, she was going to do it but she doesn't have an android, she has an iPhone. That seemed to be quite an issue" (female, GPA_6)
"I said to [the researcher] you would have to change what app you use or something because I think you potentially would get a lot more people involved with it if it was compatible with an iPhone as well" (female, GPA_6)
"And I think, I know it would cost a whole lot of money. But maybe there is mileage in providing devices for people that you know will work. So, would it, you know, giving me a mobile phone, with, so for instance, with the Fitbit, with the Lebra link or whatever it's called, on it, would that have been helpful. I don't necessarily have an answer to that. It's a question more than anything. So, then you would know it was compatible, I would know it was compatible, and we wouldn't have had all the 'phaff'. But I know there is huge cost implications around that, and other people might not want that" (female, PA_4GPA_2)
"I suppose the only downfall is so many people couldn't join this because they haven't got android" (female, GPA_6)
"Because their fancy expensive iPhone probably does everything, and makes the dinner at the same time. Whereas, my really old knackered phone doesn't. So that was frustrating" (female, PA_4GPA_2)
**UNCERTAINTY OF WHAT WAS EXPECTED FROM THEM**
**Encouragement to change lifestyle**
"If the study was again, and it said for the 6 weeks, every day or every week you have to come in and do 30 minutes exercise, that might be different. I might think okay, something I would do because I know I would have to do it as part of the study. For example, when we done the fit test. I was thinking oh my god I've got to do steps and if that was incorporated in part of the research, then I would probably do it because I know I have to" (female, PA_4GPA_2)
"I mean you could have just not bothered with it at all and just monitored it and had the Fitbit and not done anything to change. But if you were motivated, like I think I was quite motivated to use the information to help myself. I suppose you could say to somebody if you wanted to use the information to do something about it, if you are at risk, then you could try these things and then leave that option to them, what they did or maybe recommend reading if they wanted to find out more. There's a lot of information out there now" (female, GPA_6)
"I would have just, even if I wasn't working I wouldn't have, so I, you know, I can see how if a doctor or hospital or somebody said to me, 'follow whatever that says because it's going to be... I can see it's going to do me good', but until somebody tells me, 'Michelle you are going to be seriously ill unless you follow this advice that that's given you, then I would have done it" (female, GPA_6)

“I have not thought about that. You know, I could have changed it myself. I could have changed it to something lower. It didn’t occur to me. And I guess I didn’t really, if someone had said to me ‘We want you to try everything you possibly can to try and do 10,000 steps a day’, as part of this research then maybe I would have done it. But they didn’t. That’s not how it felt to me that that’s what it was about” (female, PA_4GPA_2)
“If I had been told, like what we are looking for is you to increase your activity because we are after this certain, we are trying to see this thing, then I would have gone for it” (male, G_4GPA_2)
“But if I wasn’t that person, if I was somebody else, they would say, well look what we are looking for is to have a look at your glucose levels, see if there is any, if you do have activity then people go, oh hey, you know I will try that. Well actually, I will go out on my bike. I will do an extra, you know I normally do five miles, I will do seven miles, and see what difference it makes. Oh crikey, look, it kept my glucose down. Or looking at the diet, saying you need to, as I do, I have too much starch. I am aware of that. You eat too much starch, if you had less starch you might reduce, Oh Christ, you’re right, yeah” (male, G_4GPA_2)
“But people think they have control over things and this would give you that. We would say, well actually, as part of this study, you can drive down the glucose by doing, eating different things, or exercising. People would love it” (male, G_4GPA_2)
"I think with me, if you have got to do something, I will do it. If I know I don’t have to then I will just think forget it, I don’t have to do it. It would depend if there was going to be something different in the research and if there was a bit more activity included then maybe" (female, PA_4GPA_2)
**Clear instructions were given**
“I thought the instructions were clear, what was expected of you was clear” (female, GPA_6)
“I didn’t need any more help or support, the paperwork was clear, [the researcher’s] instructions were clear and I was fine” (male, G_4GPA_2)
“She walked and talked me through putting the apps on my phone and how to use them. When I left here after everything had been set up, I felt confident that I could use it as it needed to be used” (male, PA_4GPA_2)
**Not much instruction was provided**
“Perhaps a bit more detailed, again just how to just sort of really make the best use of it would be better” (female, PA_4GPA_2)
“I just left them as they were because I didn’t know whether they were deliberately put there for the purpose of the Signal study or whether I should change them or not” (male, GPA_6)
“I suppose I understand what came up on the Fitbit but I would have liked some help on knowing how to personalise the app for me” (female, G_4GPA_2)
“I notice on your Fitbit you can change them if you want to. I didn’t change them, I don’t know if I could have but I didn’t try to” (female, GPA_6)
“I just thought by the time I had looked around and thought, ‘oh, I could do that’, I think there were only two weeks left so I thought, ‘there’s probably much point now’. If, at the beginning, somebody had said, ‘oh, have a look at your goals and if you’re meeting them all the time you might think about changing them’, I might have thought earlier” (female, GPA_6)
“I suppose if they could give you an idiots’ guide to ‘look out, your glucose has gone up more last night than average’ or something. That’s just off the top of my head now, talking to you. I wouldn’t have any expectation of that” (male, G_4GPA_2)
“There could perhaps be more literature or perhaps some examples of what sort of patterns you should expect from a normal activity. It did drop a couple of times just below the four but only very minor that was. Really just an idea of what a regular pattern is” (male, GPA_6)
“The only thing I was a bit stressed when I’d damaged one of the sensors, I wasn’t quite sure what to do. I managed to get hold of [the researcher]. I think there could perhaps be some information about if something goes wrong, what to do perhaps. That’s about the only thing, really, I can think of... I thought, ‘what should I do? Should I take it off and stick on another one or should I

just leave it?'. It didn't stop straight away, it just reported an error to start with. I think it just bent the needle and it wasn't probably getting its blood supply. It was just, 'what shall I do with it now?'" (male, GPA$_6$)

"Well I suppose I would find out whether it's, because when [the researcher] set the range, she said, 'Oh I will just set it at this'. Presumably it was compared to, because when she took my blood at the beginning, perhaps it would just need tweaking. I don't know. Yeah, I would find out whether it's normal for me to be outside the range or, I wouldn't just ignore it I don't think" (female, PA$_4$GPA$_2$)

Help with interpretation

"There's not really that much information to go with the monitor to tell you how to interpret the data" (male, GPA$_6$)

"I suppose I would be looking for what a normal, average, healthy person's graph would look like compared with mine and the interpretation of why mine might be different. That would have been very good, yes, because unless you can interpret what you're seeing you don't really know whether you should be adapting your eating and exercise and all of that" (female, G$_4$GPA$_2$)

"Perhaps if I had some information really had to actually make the best use of it. That would have probably helped. And then sort of explain what the... because I didn't really know what the readings meant, the numbers. I didn't know whether that's good or bad, or what an average person, what it should be. So, I didn't really know quite what I was looking at. That's why I just looked at the wavy lines. I suppose if something was incorporated into the app, it would probably... or... the booklet is just an extra thing, whereas if it was on the app it's there, you can refer to it" (female, PA$_4$GPA$_2$)

"I suppose one thing, now that you've mentioned it, is you could have somebody my age, with the same characteristics as me, to see what the average is across the whole population but that would be quite complicated, I think. Nonetheless, it could be another comparator that might help" (male, G$_4$GPA$_2$)

"Probably the glucose monitor, it's hard to really take anything from that. It looked like I was in the normal category for that but without being an expert on it, it's hard to actually know for that, so it's hard to take anything from that data specifically. The Fitbit is a bit more easy to understand than … I'd probably 0:23:15.7 from the Fitbit to be honest. It would probably be my favourite one, the Fitbit" (male, GPA$_6$)

"I think mainly it's the glucose, having some better understanding of what data to expect and what sort of ranges to expect. That's probably the main thing I'd say now" (male, GPA$_6$)

"I think it's more of a monitoring thing, it's probably best to have an idea of where you should be and what sort of pattern you should expect" (male, GPA$_6$)

"The app does have some information bars on it, so you can actually look at some information on it but it doesn't really tell you what's a good value or a bad value or anything like that. It might just have been nice to have some indication of that" (male, GPA$_6$)

"Yes, it does go up when you've eaten but I'm just really curious as to how much it should actually be allowed to go outside of the band that you're targeting going in round. It doesn't give you any, it does actually give you some charts I think, for time and target and stuff like that. It does give you bands for each one but what good figures are I would perhaps be more interested in as well... Yes, it just tells you basically what it's showing you, really. It doesn't tell you how to interpret the data. Perhaps on the information bar it could actually tell you, 'yes, this is a good target to be in each of the bands for'" (male, GPA$_6$)

"They say, 'what does that mean?' and you say, 'I don't really know, I'm supposed to stay in the green line'" (male, GPA$_6$)

Liked not being told

"But [the researcher] did say at the start, use this as you want it's your, your thing to use as you want. So that was good" (male, G$_4$GPA$_2$)

"I thought the study parameters for the participants were quite relaxed. There weren't very many rules about what you ought to do or not to do. I haven't been told to do that or not to do it and I haven't been told to change my behaviour or not to change my behaviour, so I can change it if I want to carry on. Also, I don't have to monitor it myself, so I could have scanned it a couple of times and then not looked at it. There was nobody forcing me to check on any of it or to log activities the Fitbit didn't log on its

own. I did guess that the data would be more useful, the more of it there was because the Fitbit can't know I've been swimming unless I put that in and that sort of thing" (female, GPA_6)
"I know it is quite hard to change your behaviour and that was one of the things that I liked – that I could but equally I could not" (female, GPA_6)
**Engagement**
"I thought it was more for monitoring here, rather than my own target-setting" (male, GPA_6)
"I try not to eat too unhealthily again, I thought since this isn't really a study that's looking at what people eat and presumably you wouldn't want me to change too much of what I was doing if you were just measuring certain things in the study which meant I could go ahead as normal" (female, GPA_6)
"I was a bit vague as to whether I should go and do that because as I understand it, the study is about how the wearer this technology adapts their routine because of what they see but I didn't know whether it was what you can actually see or whether you should be able to fully interpret what you see, if you follow me?" (female, G_4GPA_2)
"No, unless I could have been given some help with interpreting what I was seeing but as I say I didn't know whether I was supposed to have that. I didn't know whether I was supposed to understand what I was seeing because I thought it might make me change my behaviour and I didn't know if you wanted me to change my behaviour" (female, G_4GPA_2)
"I think my engagement was very limited because I think I was remotely monitored most of the time. Obviously, stuff went up to the internet and then I know that [the researcher] noticed once that the Fitbit hadn't synchronised, so she actually contacted me. Apart from that, I didn't feel like I was engaged in doing anything because I wasn't aware that I had to do anything other than what I did normally, so I think that was fairly random there. There was no active engagement. There were a few emails back and forth with [the researcher] for certain things but that was about it, really. It was more just the monitoring I suppose" (male, GPA_6)
"I suppose because I have been quite well informed about diet, diabetes, exercise affecting…, I suppose it depends what you were aiming to find out because it wasn't really about us, what it was going to help us. I mean it was optional, wasn't it, for us" (female, GPA_6)
"Not really because you're not told why you're doing it… you are told a little bit why you are doing it" (female, GPA_6)
"A lot of people have said to me, 'what is it for?' and I said, 'I think it's to do with people who have got diabetes' because one of the criteria was somebody in the family had to have it. Secondly, it's about how all this is collated on your phone and how you interact with it and how often you interact with it. I said, 'I think that's it but I don't know exactly'. I think I would like to have known" (female, PA_4GPA_2)
