# Supplementary material for: Examining the Use of Glucose and Physical Activity Self-Monitoring Technologies in Individuals at Moderate to High Risk of Developing Type 2 Diabetes: Randomized Trial
Source: JMIR Mhealth Uhealth. 2019 Oct 28;7(10):e14195. doi: 10.2196/14195 (PMC6913728; doi:10.2196/14195)
Supplement: Multimedia Appendix 8 [file mhealth_v7i10e14195_app8.pdf]

CONSORT-EHEALTH (V 1.6.1) - Submission/Publication Form

The CONSORT-EHEALTH checklist is intended for authors of randomized trials evaluating web-based and Internet-based applications/interventions, including mobile interventions, electronic games (incl multiplayer games), social media, certain telehealth applications, and other interactive and/or networked electronic applications. Some of the items (e.g. all subitems under item 5 - description of the intervention) may also be applicable for other study designs.

The goal of the CONSORT EHEALTH checklist and guideline is to be
a) a guide for reporting for authors of RCTs,
b) to form a basis for appraisal of an ehealth trial (in terms of validity)

CONSORT-EHEALTH items/subitems are MANDATORY reporting items for studies published in the Journal of Medical Internet Research and other journals / scientific societies endorsing the checklist.

Items numbered 1., 2., 3., 4a., 4b etc are original CONSORT or CONSORT-NPT (non-pharmacologic treatment) items.
Items with Roman numerals (i., ii, iii, iv etc.) are CONSORT-EHEALTH extensions/clarifications.

As the CONSORT-EHEALTH checklist is still considered in a formative stage, we would ask that you also RATE ON A SCALE OF 1-5 how important/useful you feel each item is FOR THE PURPOSE OF THE CHECKLIST and reporting guideline (optional).

Mandatory reporting items are marked with a red *.
In the textboxes, either copy & paste the relevant sections from your manuscript into this form - please include any quotes from your manuscript in QUOTATION MARKS,
or answer directly by providing additional information not in the manuscript, or elaborating on why the item was not relevant for this study.

YOUR ANSWERS WILL BE PUBLISHED AS A SUPPLEMENTARY FILE TO YOUR PUBLICATION IN JMIR AND ARE CONSIDERED PART OF YOUR PUBLICATION (IF ACCEPTED).
Please fill in these questions diligently. Information will not be copyedited, so please use proper spelling and grammar, use correct capitalization, and avoid abbreviations.

DO NOT FORGET TO SAVE AS PDF _AND_ CLICK THE SUBMIT BUTTON SO YOUR ANSWERS ARE IN OUR DATABASE !!!

Citation Suggestion (if you append the pdf as Appendix we suggest to cite this paper in the caption):
Eysenbach G, CONSORT-EHEALTH Group
CONSORT-EHEALTH: Improving and Standardizing Evaluation Reports of Web-based and Mobile Health Interventions
J Med Internet Res 2011;13(4):e126
URL: http://www.jmir.org/2011/4/e126/
doi: 10.2196/jmir.1923
PMID: 22209829

* Required

Your name *
First Last

Whelan Maxine

Primary Affiliation (short), City, Country *
University of Toronto, Toronto, Canada

University of Oxford, Oxford, United King

Your e-mail address *
abc@gmail.com

maxine.whelan@phc.ox.ac.uk

Title of your manuscript *
Provide the (draft) title of your manuscript.

Findings of Sensing Interstitial Glucose to Nudge Active Lifestyles (SIGNAL): A randomised trial examining user engagement with glucose and physical activity self-monitoring technologies in individuals at moderate-to-high risk of developing type 2 diabetes

Name of your App/Software/Intervention *
If there is a short and a long/alternate name, write the short name first and add the long name in brackets.

SIGNAL intervention (Sensing Interstitial

Evaluated Version (if any)
e.g. "V1", "Release 2017-03-01", "Version 2.0.27913"

Your answer

Language(s) *

What language is the intervention/app in? If multiple languages are available, separate by comma (e.g. "English, French")

English

URL of your Intervention Website or App

e.g. a direct link to the mobile app on app in appstore (itunes, Google Play), or URL of the website. If the intervention is a DVD or hardware, you can also link to an Amazon page.

Your answer

URL of an image/screenshot (optional)

Your answer

Accessibility *

Can an enduser access the intervention presently?

- ○ access is free and open
- ○ access only for special usergroups, not open
- ○ access is open to everyone, but requires payment/subscription/in-app purchases
- ○ app/intervention no longer accessible
- ◉ Other: Technologies used in SIGNAL intervention accessible

Primary Medical Indication/Disease/Condition *

e.g. "Stress", "Diabetes", or define the target group in brackets after the condition, e.g. "Autism (Parents of children with)", "Alzheimers (Informal Caregivers of)"

Prevention of diabetes

Primary Outcomes measured in trial *

comma-separated list of primary outcomes reported in the trial

Participant engagement

Secondary/other outcomes

Are there any other outcomes the intervention is expected to affect?

Feasibility and acceptability

Recommended "Dose" *

What do the instructions for users say on how often the app should be used?

- (x) Approximately Daily
- ( ) Approximately Weekly
- ( ) Approximately Monthly
- ( ) Approximately Yearly
- ( ) "as needed"
- ( ) Other:

Approx. Percentage of Users (starters) still using the app as recommended after 3 months *

- (selected) unknown / not evaluated
- 0-10%
- 11-20%
- 21-30%
- 31-40%
- 41-50%
- 51-60%
- 61-70%
- 71%-80%
- 81-90%
- 91-100%
- Other:

Overall, was the app/intervention effective? *

- ( ) yes: all primary outcomes were significantly better in intervention group vs control
- ( ) partly: SOME primary outcomes were significantly better in intervention group vs control
- ( ) no statistically significant difference between control and intervention
- ( ) potentially harmful: control was significantly better than intervention in one or more outcomes
- ( ) inconclusive: more research is needed
- (x) Other: Not powered to assess effectiveness

Article Preparation Status/Stage *

At which stage in your article preparation are you currently (at the time you fill in this form)

- ( ) not submitted yet - in early draft status
- (x) not submitted yet - in late draft status, just before submission
- ( ) submitted to a journal but not reviewed yet
- ( ) submitted to a journal and after receiving initial reviewer comments
- ( ) submitted to a journal and accepted, but not published yet
- ( ) published
- ( ) Other:

Journal *

If you already know where you will submit this paper (or if it is already submitted), please provide the journal name (if it is not JMIR, provide the journal name under "other")

- ( ) not submitted yet / unclear where I will submit this
- (x) Journal of Medical Internet Research (JMIR)
- ( ) JMIR mHealth and UHealth
- ( ) JMIR Serious Games
- ( ) JMIR Mental Health
- ( ) JMIR Public Health
- ( ) JMIR Formative Research
- ( ) Other JMIR sister journal
- ( ) Other:

Is this a full powered effectiveness trial or a pilot/feasibility trial? *

- (x) Pilot/feasibility
- ( ) Fully powered

Manuscript tracking number *

If this is a JMIR submission, please provide the manuscript tracking number under "other" (The ms tracking number can be found in the submission acknowledgement email, or when you login as author in JMIR. If the paper is already published in JMIR, then the ms tracking number is the four-digit number at the end of the DOI, to be found at the bottom of each published article in JMIR)

- (x) no ms number (yet) / not (yet) submitted to / published in JMIR
- ( ) Other:

TITLE AND ABSTRACT

1a) TITLE: Identification as a randomized trial in the title

1a) Does your paper address CONSORT item 1a? *

I.e does the title contain the phrase "Randomized Controlled Trial"? (if not, explain the reason under "other")

- ( ) yes
- (•) Other: It states "randomized trial"

1a-i) Identify the mode of delivery in the title

Identify the mode of delivery. Preferably use “web-based” and/or “mobile” and/or “electronic game” in the title. Avoid ambiguous terms like “online”, “virtual”, “interactive”. Use “Internet-based” only if Intervention includes non-web-based Internet components (e.g. email), use “computer-based” or “electronic” only if offline products are used. Use “virtual” only in the context of “virtual reality” (3-D worlds). Use “online” only in the context of “online support groups”. Complement or substitute product names with broader terms for the class of products (such as “mobile” or “smart phone” instead of “iphone”), especially if the application runs on different platforms.

	1	2	3	4	5	
subitem not at all important	( )	( )	( )	(•)	( )	essential

Does your paper address subitem 1a-i? *

Copy and paste relevant sections from manuscript title (include quotes in quotation marks "like this" to indicate direct quotes from your manuscript), or elaborate on this item by providing additional information not in the ms, or briefly explain why the item is not applicable/relevant for your study

"glucose and physical activity self-monitoring technologies"

1a-ii) Non-web-based components or important co-interventions in title

Mention non-web-based components or important co-interventions in title, if any (e.g., “with telephone support”).

	1	2	3	4	5	
subitem not at all important	( )	( )	( )	(•)	( )	essential

Does your paper address subitem 1a-ii?

Copy and paste relevant sections from manuscript title (include quotes in quotation marks "like this" to indicate direct quotes from your manuscript), or elaborate on this item by providing additional information not in the ms, or briefly explain why the item is not applicable/relevant for your study

Not relevant for this study.

1a-iii) Primary condition or target group in the title

Mention primary condition or target group in the title, if any (e.g., "for children with Type I Diabetes") Example: A Web-based and Mobile Intervention with Telephone Support for Children with Type I Diabetes: Randomized Controlled Trial

	1	2	3	4	5	
subitem not at all important	○	○	○	●	○	essential

Does your paper address subitem 1a-iii? *

Copy and paste relevant sections from manuscript title (include quotes in quotation marks "like this" to indicate direct quotes from your manuscript), or elaborate on this item by providing additional information not in the ms, or briefly explain why the item is not applicable/relevant for your study

"individuals at moderate-to-high risk of developing type 2 diabetes"

1b) ABSTRACT: Structured summary of trial design, methods, results, and conclusions

NPT extension: Description of experimental treatment, comparator, care providers, centers, and blinding status.

1b-i) Key features/functionalities/components of the intervention and comparator in the METHODS section of the ABSTRACT

Mention key features/functionalities/components of the intervention and comparator in the abstract. If possible, also mention theories and principles used for designing the site. Keep in mind the needs of systematic reviewers and indexers by including important synonyms. (Note: Only report in the abstract what the main paper is reporting. If this information is missing from the main body of text, consider adding it)

	1	2	3	4	5	
subitem not at all important	○	○	○	●	○	essential

Does your paper address subitem 1b-i? *

Copy and paste relevant sections from the manuscript abstract (include quotes in quotation marks "like this" to indicate direct quotes from your manuscript), or elaborate on this item by providing additional information not in the ms, or briefly explain why the item is not applicable/relevant for your study

"Each participant was provided with a behavioural (Fitbit Charge 2) and physiological (Freestyle Libre flash glucose monitor) monitor for six weeks; masked according to group allocation... Participant engagement (time spent on the applications, number of glucose scans, number of syncs and changes to physical activity goals) was the primary outcome. Secondary outcomes were the feasibility (including recruitment, eligibility and number of sensor displacements) and acceptability (including monitor wear time and missing data) of the intervention. Semi-structured qualitative interviews were conducted at the 6-week follow up appointment to explore their experience of using the technology and study participation."

1b-ii) Level of human involvement in the METHODS section of the ABSTRACT

Clarify the level of human involvement in the abstract, e.g., use phrases like “fully automated” vs. “therapist/nurse/care provider/physician-assisted” (mention number and expertise of providers involved, if any). (Note: Only report in the abstract what the main paper is reporting. If this information is missing from the main body of text, consider adding it)

	1	2	3	4	5	
subitem not at all important	○	○	◉	○	○	essential

Does your paper address subitem 1b-ii?

Copy and paste relevant sections from the manuscript abstract (include quotes in quotation marks "like this" to indicate direct quotes from your manuscript), or elaborate on this item by providing additional information not in the ms, or briefly explain why the item is not applicable/relevant for your study

Details found in main Methods section of the paper.

1b-iii) Open vs. closed, web-based (self-assessment) vs. face-to-face assessments in the METHODS section of the ABSTRACT

Mention how participants were recruited (online vs. offline), e.g., from an open access website or from a clinic or a closed online user group (closed usergroup trial), and clarify if this was a purely web-based trial, or there were face-to-face components (as part of the intervention or for assessment). Clearly say if outcomes were self-assessed through questionnaires (as common in web-based trials). Note: In traditional offline trials, an open trial (open-label trial) is a type of clinical trial in which both the researchers and participants know which treatment is being administered. To avoid confusion, use “blinded” or “unblinded” to indicated the level of blinding instead of “open”, as “open” in web-based trials usually refers to “open access” (i.e. participants can self-enrol). (Note: Only report in the abstract what the main paper is reporting. If this information is missing from the main body of text, consider adding it)

	1	2	3	4	5	
subitem not at all important	○	○	○	◉	○	essential

Does your paper address subitem 1b-iii?

Copy and paste relevant sections from the manuscript abstract (include quotes in quotation marks "like this" to indicate direct quotes from your manuscript), or elaborate on this item by providing additional information not in the ms, or briefly explain why the item is not applicable/relevant for your study

Details found in main Methods section of the paper.

1b-iv) RESULTS section in abstract must contain use data

Report number of participants enrolled/assessed in each group, the use/uptake of the intervention (e.g., attrition/adherence metrics, use over time, number of logins etc.), in addition to primary/secondary outcomes. (Note: Only report in the abstract what the main paper is reporting. If this information is missing from the main body of text, consider adding it)

	1	2	3	4	5	
subitem not at all important	○	○	○	◉	○	essential

Does your paper address subitem 1b-iv?

Copy and paste relevant sections from the manuscript abstract (include quotes in quotation marks "like this" to indicate direct quotes from your manuscript), or elaborate on this item by providing additional information not in the ms, or briefly explain why the item is not applicable/relevant for your study

"Engagement: Time spent on the Fitbit and Freestyle Libre applications declined over the six weeks for all groups. Of the Freestyle Libre sensor scans conducted by participants, 17% recorded rising or falling trends in glucose, and 13 of 45 participants (23.9%) changed ≥1 of the physical activity goals. Feasibility: 22 of 45 participants (48.9%) completed the study using the minimum number of Freestyle Libre sensors and a total of 41 sensors were declared faulty or displaced during the study. Acceptability: Participants wore the Fitbit for 40.1±3.2 days and 9 of 45 (20%) and 24 of 45 participants (53.3%) received a prompt by email to charge or sync the Fitbit, respectively. Interviews unearthed participant perceptions on the study design, by suggesting refinements to the eligibility criteria as well as highlighting important issues about the usability, wearability, reliability, durability, privacy, features and cost of the technologies."

1b-v) CONCLUSIONS/DISCUSSION in abstract for negative trials

Conclusions/Discussions in abstract for negative trials: Discuss the primary outcome - if the trial is negative (primary outcome not changed), and the intervention was not used, discuss whether negative results are attributable to lack of uptake and discuss reasons. (Note: Only report in the abstract what the main paper is reporting. If this information is missing from the main body of text, consider adding it)

	1	2	3	4	5	
subitem not at all important	○	○	○	◉	○	essential

Does your paper address subitem 1b-v?

Copy and paste relevant sections from the manuscript abstract (include quotes in quotation marks "like this" to indicate direct quotes from your manuscript), or elaborate on this item by providing additional information not in the ms, or briefly explain why the item is not applicable/relevant for your study

"Modifications are required to the study and to commercially available technologies"

INTRODUCTION

2a) In INTRODUCTION: Scientific background and explanation of rationale

2a-i) Problem and the type of system/solution

Describe the problem and the type of system/solution that is object of the study: intended as stand-alone intervention vs. incorporated in broader health care program? Intended for a particular patient population? Goals of the intervention, e.g., being more cost-effective to other interventions, replace or complement other solutions? (Note: Details about the intervention are provided in “Methods” under 5)

	1	2	3	4	5	
subitem not at all important	○	○	○	◉	○	essential

Does your paper address subitem 2a-i? *

Copy and paste relevant sections from the manuscript (include quotes in quotation marks "like this" to indicate direct quotes from your manuscript), or elaborate on this item by providing additional information not in the ms, or briefly explain why the item is not applicable/relevant for your study

"prevalence of diabetes was estimated to be more than three million in England in 2017" "need to prioritise prevention over cure for long-term conditions" "new opportunities for people to actively participate in their healthcare in non-clinical settings" "opportunity for individuals to see these relationships in real-time" "this technology has not been examined in the context of diabetes prevention"

2a-ii) Scientific background, rationale: What is known about the (type of) system

Scientific background, rationale: What is known about the (type of) system that is the object of the study (be sure to discuss the use of similar systems for other conditions/diagnoses, if appropiate), motivation for the study, i.e. what are the reasons for and what is the context for this specific study, from which stakeholder viewpoint is the study performed, potential impact of findings [2]. Briefly justify the choice of the comparator.

	1	2	3	4	5	
subitem not at all important	○	○	○	◉	○	essential

Does your paper address subitem 2a-ii? *

Copy and paste relevant sections from the manuscript (include quotes in quotation marks "like this" to indicate direct quotes from your manuscript), or elaborate on this item by providing additional information not in the ms, or briefly explain why the item is not applicable/relevant for your study

"Early studies have demonstrated reductions in hypoglycaemia, increased time spent in the target range and greater levels of patient satisfaction compared with traditional fingerstick monitoring for individuals living with type 1 and 2 diabetes.[7], [8]" "In the laboratory setting, brief bouts of physical activity have resulted in acute reductions in postprandial glucose and insulin in normal weight, overweight and obese adults.[11]–[13]"

2b) In INTRODUCTION: Specific objectives or hypotheses

Does your paper address CONSORT subitem 2b? *

Copy and paste relevant sections from the manuscript (include quotes in quotation marks "like this" to indicate direct quotes from your manuscript), or elaborate on this item by providing additional information not in the ms, or briefly explain why the item is not applicable/relevant for your study

"examined user engagement with FGM and physical activity self-monitoring technologies for people identified as moderate-to-high risk of developing type 2 diabetes. Secondary objectives were to assess the feasibility and acceptability of the trial design, methodology and the technology"

METHODS

3a) Description of trial design (such as parallel, factorial) including allocation ratio

Does your paper address CONSORT subitem 3a? *

Copy and paste relevant sections from the manuscript (include quotes in quotation marks "like this" to indicate direct quotes from your manuscript), or elaborate on this item by providing additional information not in the ms, or briefly explain why the item is not applicable/relevant for your study

"this trial was a randomised, three-arm feasibility trial" "with 1:1:1 allocation"

3b) Important changes to methods after trial commencement (such as eligibility criteria), with reasons

Does your paper address CONSORT subitem 3b? *

Copy and paste relevant sections from the manuscript (include quotes in quotation marks "like this" to indicate direct quotes from your manuscript), or elaborate on this item by providing additional information not in the ms, or briefly explain why the item is not applicable/relevant for your study

No important changes to the study were made after trial commencement.

3b-i) Bug fixes, Downtimes, Content Changes

Bug fixes, Downtimes, Content Changes: ehealth systems are often dynamic systems. A description of changes to methods therefore also includes important changes made on the intervention or comparator during the trial (e.g., major bug fixes or changes in the functionality or content) (5-iii) and other “unexpected events” that may have influenced study design such as staff changes, system failures/downtimes, etc. [2].

	1	2	3	4	5	
subitem not at all important	○	○	○	◉	○	essential

Does your paper address subitem 3b-i?

Copy and paste relevant sections from the manuscript (include quotes in quotation marks "like this" to indicate direct quotes from your manuscript), or elaborate on this item by providing additional information not in the ms, or briefly explain why the item is not applicable/relevant for your study

No changes to the two self-monitoring technologies were made during the

4a) Eligibility criteria for participants

Does your paper address CONSORT subitem 4a? *

Copy and paste relevant sections from the manuscript (include quotes in quotation marks "like this" to indicate direct quotes from your manuscript), or elaborate on this item by providing additional information not in the ms, or briefly explain why the item is not applicable/relevant for your study

"participants were aged ≥40 years and owned a compatible Android smartphone"
"Interested individuals were directed to complete The Leicester Risk Assessment[15] to determine level of risk for type 2 diabetes via an online survey (Qualtrics, Provo, Utah, USA) (Appendix 1). After completing the online survey, individuals who received a score of moderate (16-24 points) or high (≥25 points out of 47) risk were contacted by a researcher and sent the participant information sheet."
Readers are referred also to the published protocol - "The full protocol has been published[14] and the study was registered prospectively (ISRCTN17545949)"

4a-i) Computer / Internet literacy

Computer / Internet literacy is often an implicit “de facto” eligibility criterion - this should be explicitly clarified.

	1	2	3	4	5	
subitem not at all important	○	○	○	●	○	essential

Does your paper address subitem 4a-i?

Copy and paste relevant sections from the manuscript (include quotes in quotation marks "like this" to indicate direct quotes from your manuscript), or elaborate on this item by providing additional information not in the ms, or briefly explain why the item is not applicable/relevant for your study

Due to the feasibility nature of the study, no criteria for computer/internet literacy was incorporated into the eligibility criteria.

4a-ii) Open vs. closed, web-based vs. face-to-face assessments:

Open vs. closed, web-based vs. face-to-face assessments: Mention how participants were recruited (online vs. offline), e.g., from an open access website or from a clinic, and clarify if this was a purely web-based trial, or there were face-to-face components (as part of the intervention or for assessment), i.e., to what degree got the study team to know the participant. In online-only trials, clarify if participants were quasi-anonymous and whether having multiple identities was possible or whether technical or logistical measures (e.g., cookies, email confirmation, phone calls) were used to detect/prevent these.

	1	2	3	4	5	
subitem not at all important	○	○	○	●	○	essential

Does your paper address subitem 4a-ii? *

Copy and paste relevant sections from the manuscript (include quotes in quotation marks "like this" to indicate direct quotes from your manuscript), or elaborate on this item by providing additional information not in the ms, or briefly explain why the item is not applicable/relevant for your study

"Participants were recruited between May and September 2017 by circulating posters, letters and emails across Leicestershire, UK" "After eligibility was ascertained via email or telephone by a researcher, an appointment was scheduled at Loughborough University" "Interviews were completed at the six-week follow up appointment by a member of the research team independent from the quantitative data collection procedures and intervention delivery"

4a-iii) Information giving during recruitment

Information given during recruitment. Specify how participants were briefed for recruitment and in the informed consent procedures (e.g., publish the informed consent documentation as appendix, see also item X26), as this information may have an effect on user self-selection, user expectation and may also bias results.

	1	2	3	4	5	
subitem not at all important	○	○	○	◉	○	essential

Does your paper address subitem 4a-iii?

Copy and paste relevant sections from the manuscript (include quotes in quotation marks "like this" to indicate direct quotes from your manuscript), or elaborate on this item by providing additional information not in the ms, or briefly explain why the item is not applicable/relevant for your study

"After completing the online survey, individuals who received a score of moderate (16-24 points) or high (≥25 points out of 47) risk were contacted by a researcher and sent the participant information sheet. After eligibility was ascertained via email or telephone by a researcher, an appointment was scheduled at Loughborough University."

4b) Settings and locations where the data were collected

Does your paper address CONSORT subitem 4b? *

Copy and paste relevant sections from the manuscript (include quotes in quotation marks "like this" to indicate direct quotes from your manuscript), or elaborate on this item by providing additional information not in the ms, or briefly explain why the item is not applicable/relevant for your study

"by circulating posters, letters and emails across Leicestershire, UK" "an appointment was scheduled at Loughborough University"

4b-i) Report if outcomes were (self-)assessed through online questionnaires

Clearly report if outcomes were (self-)assessed through online questionnaires (as common in web-based trials) or otherwise.

	1	2	3	4	5	
subitem not at all important	○	○	○	○	◉	essential

Does your paper address subitem 4b-i? *

Copy and paste relevant sections from the manuscript (include quotes in quotation marks "like this" to indicate direct quotes from your manuscript), or elaborate on this item by providing additional information not in the ms, or briefly explain why the item is not applicable/relevant for your study

All questionnaires were self-reported and paper-based.

4b-ii) Report how institutional affiliations are displayed

Report how institutional affiliations are displayed to potential participants [on ehealth media], as affiliations with prestigious hospitals or universities may affect volunteer rates, use, and reactions with regards to an intervention.(Not a required item – describe only if this may bias results)

	1	2	3	4	5	
subitem not at all important	○	○	○	○	○	essential

Does your paper address subitem 4b-ii?

Copy and paste relevant sections from the manuscript (include quotes in quotation marks "like this" to indicate direct quotes from your manuscript), or elaborate on this item by providing additional information not in the ms, or briefly explain why the item is not applicable/relevant for your study

Institutional affiliations were presented as logos on participant-facing documents.

5) The interventions for each group with sufficient details to allow replication, including how and when they were actually administered

5-i) Mention names, credential, affiliations of the developers, sponsors, and owners

Mention names, credential, affiliations of the developers, sponsors, and owners [6] (if authors/evaluators are owners or developer of the software, this needs to be declared in a “Conflict of interest” section or mentioned elsewhere in the manuscript).

	1	2	3	4	5	
subitem not at all important	○	○	◉	○	○	essential

Does your paper address subitem 5-i?

Copy and paste relevant sections from the manuscript (include quotes in quotation marks "like this" to indicate direct quotes from your manuscript), or elaborate on this item by providing additional information not in the ms, or briefly explain why the item is not applicable/relevant for your study

Various devices and software were used in this trial including: "online survey (Qualtrics, Provo, Utah, USA)" "Fitbit Charge 2 (Fitbit Inc., San Francisco, CA" "Freestyle Libre (Abbott Diabetes Care, Alameda, CA)" "Fitabase (Small Steps Labs LLC., California, USA)" "Kinesoft version 3.3.80 (Kinesoft, Loughborough, UK)" "Diasend (Diasend Inc., Illinois, USA)" "accelerometers (ActiGraph, Pensacola, FL, USA)" "ActiLife (ActiGraph, Florida, USA)" "Ethica Data (Kitchener, Ontario, Canada)"

5-ii) Describe the history/development process

Describe the history/development process of the application and previous formative evaluations (e.g., focus groups, usability testing), as these will have an impact on adoption/use rates and help with interpreting results.

	1	2	3	4	5	
subitem not at all important	○	○	○	◉	○	essential

Does your paper address subitem 5-ii?

Copy and paste relevant sections from the manuscript (include quotes in quotation marks "like this" to indicate direct quotes from your manuscript), or elaborate on this item by providing additional information not in the ms, or briefly explain why the item is not applicable/relevant for your study

Patient and public involvement was conducted for this trial, involving people with type 2 diabetes and people without to discuss the recruitment approach and the study design (duration of access to the self-monitoring technologies).

5-iii) Revisions and updating

Revisions and updating. Clearly mention the date and/or version number of the application/intervention (and comparator, if applicable) evaluated, or describe whether the intervention underwent major changes during the evaluation process, or whether the development and/or content was “frozen” during the trial. Describe dynamic components such as news feeds or changing content which may have an impact on the replicability of the intervention (for unexpected events see item 3b).

	1	2	3	4	5	
subitem not at all important	○	○	○	○	◉	essential

Does your paper address subitem 5-iii?

Copy and paste relevant sections from the manuscript (include quotes in quotation marks "like this" to indicate direct quotes from your manuscript), or elaborate on this item by providing additional information not in the ms, or briefly explain why the item is not applicable/relevant for your study

No changes were made during the study.

5-iv) Quality assurance methods

Provide information on quality assurance methods to ensure accuracy and quality of information provided [1], if applicable.

	1	2	3	4	5	
subitem not at all important	○	○	○	◉	○	essential

Does your paper address subitem 5-iv?

Copy and paste relevant sections from the manuscript (include quotes in quotation marks "like this" to indicate direct quotes from your manuscript), or elaborate on this item by providing additional information not in the ms, or briefly explain why the item is not applicable/relevant for your study

Your answer

5-v) Ensure replicability by publishing the source code, and/or providing screenshots/screen-capture video, and/or providing flowcharts of the algorithms used

Ensure replicability by publishing the source code, and/or providing screenshots/screen-capture video, and/or providing flowcharts of the algorithms used. Replicability (i.e., other researchers should in principle be able to replicate the study) is a hallmark of scientific reporting.

	1	2	3	4	5	
subitem not at all important	○	○	○	◉	○	essential

Does your paper address subitem 5-v?

Copy and paste relevant sections from the manuscript (include quotes in quotation marks "like this" to indicate direct quotes from your manuscript), or elaborate on this item by providing additional information not in the ms, or briefly explain why the item is not applicable/relevant for your study

The online survey questions used to identify people at moderate-to-high risk of developing type 2 diabetes is presented in Multi-media Appendix 1. The self-monitoring technologies are clearly named in the manuscript "Fitbit Charge 2" and "Freestyle Libre" and commercially available so no screenshots are provided.

5-vi) Digital preservation

Digital preservation: Provide the URL of the application, but as the intervention is likely to change or disappear over the course of the years; also make sure the intervention is archived (Internet Archive, webcitation.org, and/or publishing the source code or screenshots/videos alongside the article). As pages behind login screens cannot be archived, consider creating demo pages which are accessible without login.

	1	2	3	4	5	
subitem not at all important	○	○	○	◉	○	essential

Does your paper address subitem 5-vi?

Copy and paste relevant sections from the manuscript (include quotes in quotation marks "like this" to indicate direct quotes from your manuscript), or elaborate on this item by providing additional information not in the ms, or briefly explain why the item is not applicable/relevant for your study

Screenshots are not provided in this paper.

5-vii) Access

Access: Describe how participants accessed the application, in what setting/context, if they had to pay (or were paid) or not, whether they had to be a member of specific group. If known, describe how participants obtained “access to the platform and Internet” [1]. To ensure access for editors/reviewers/readers, consider to provide a “backdoor” login account or demo mode for reviewers/readers to explore the application (also important for archiving purposes, see vi).

	1	2	3	4	5	
subitem not at all important	○	○	○	◉	○	essential

Does your paper address subitem 5-vii? *

Copy and paste relevant sections from the manuscript (include quotes in quotation marks "like this" to indicate direct quotes from your manuscript), or elaborate on this item by providing additional information not in the ms, or briefly explain why the item is not applicable/relevant for your study

"The full protocol has been published[14]" "owned a compatible Android smartphone"

5-viii) Mode of delivery, features/functionalities/components of the intervention and comparator, and the theoretical framework

Describe mode of delivery, features/functionalities/components of the intervention and comparator, and the theoretical framework [6] used to design them (instructional strategy [1], behaviour change techniques, persuasive features, etc., see e.g., [7, 8] for terminology). This includes an in-depth description of the content (including where it is coming from and who developed it) [1]," whether [and how] it is tailored to individual circumstances and allows users to track their progress and receive feedback" [6]. This also includes a description of communication delivery channels and – if computer-mediated communication is a component – whether communication was synchronous or asynchronous [6]. It also includes information on presentation strategies [1], including page design principles, average amount of text on pages, presence of hyperlinks to other resources, etc. [1].

	1	2	3	4	5	
subitem not at all important	○	○	○	○	◉	essential

Does your paper address subitem 5-viii? *

Copy and paste relevant sections from the manuscript (include quotes in quotation marks "like this" to indicate direct quotes from your manuscript), or elaborate on this item by providing additional information not in the ms, or briefly explain why the item is not applicable/relevant for your study

"The full protocol has been published[14]" "Interstitial glucose levels were categorised as below range (<4.0 mmol/L), normal (4.0-5.9 mmol/L) or above range (>5.9 mmol/L).[16] Scans were also characterised as rising quickly, rising, changing slowly, falling, falling quickly or no trend arrow (determined by proprietary algorithms)." "Default Fitbit physical activity goals were 10,000 steps, 30 active minutes, 10 flights of stairs, 2500 calories and 8 kilometres per day."

5-ix) Describe use parameters

Describe use parameters (e.g., intended "doses" and optimal timing for use). Clarify what instructions or recommendations were given to the user, e.g., regarding timing, frequency, heaviness of use, if any, or was the intervention used ad libitum.

	1	2	3	4	5	
subitem not at all important	○	○	○	○	◉	essential

Does your paper address subitem 5-ix?

Copy and paste relevant sections from the manuscript (include quotes in quotation marks "like this" to indicate direct quotes from your manuscript), or elaborate on this item by providing additional information not in the ms, or briefly explain why the item is not applicable/relevant for your study

"The full protocol has been published[14]" "Participants were encouraged to use the self-monitoring technologies as they wished with no expectation or judgement from the researchers." "Participants were asked to ensure the Fitbit had enough charge and synced regularly" "For the Freestyle Libre, participants were asked to scan the sensor once every 7-8 hours to minimise data loss but were not reminded by researchers if they failed to adhere to this." "Participants were instructed to wear them during waking hours and remove for water-based activities"

5-x) Clarify the level of human involvement

Clarify the level of human involvement (care providers or health professionals, also technical assistance) in the e-intervention or as co-intervention (detail number and expertise of professionals involved, if any, as well as "type of assistance offered, the timing and frequency of the support, how it is initiated, and the medium by which the assistance is delivered". It may be necessary to distinguish between the level of human involvement required for the trial, and the level of human involvement required for a routine application outside of a RCT setting (discuss under item 21 – generalizability).

	1	2	3	4	5	
subitem not at all important	○	○	○	○	◉	essential

Does your paper address subitem 5-x?

Copy and paste relevant sections from the manuscript (include quotes in quotation marks "like this" to indicate direct quotes from your manuscript), or elaborate on this item by providing additional information not in the ms, or briefly explain why the item is not applicable/relevant for your study

"Participants were encouraged to use the self-monitoring technologies as they wished with no expectation or judgement from the researchers." "Before participants left the appointment, verbal and written information was provided about how to apply, activate and scan the Freestyle Libre and how to sync and charge the Fitbit. "
Figure 1 outlines the study protocol, specifically the event of an additional appointment.

5-xi) Report any prompts/reminders used

Report any prompts/reminders used: Clarify if there were prompts (letters, emails, phone calls, SMS) to use the application, what triggered them, frequency etc. It may be necessary to distinguish between the level of prompts/reminders required for the trial, and the level of prompts/reminders for a routine application outside of a RCT setting (discuss under item 21 – generalizability).

	1	2	3	4	5	
subitem not at all important	○	○	○	◉	○	essential

Does your paper address subitem 5-xi? *

Copy and paste relevant sections from the manuscript (include quotes in quotation marks "like this" to indicate direct quotes from your manuscript), or elaborate on this item by providing additional information not in the ms, or briefly explain why the item is not applicable/relevant for your study

"For the Freestyle Libre, participants were asked to scan the sensor once every 7-8 hours to minimise data loss but were not reminded by researchers if they failed to adhere to this." "Participants were asked to ensure the Fitbit had enough charge and synced regularly with the Fitbit application during the intervention. Participants were notified in the event battery level reached <25% or if ≥5 days had passed since a previous sync (both remotely monitored by the researchers using Fitabase). For the Freestyle Libre, participants were asked to scan the sensor once every 7-8 hours to minimise data loss but were not reminded by researchers if they failed to adhere to this. This was not done for those masked to glucose feedback, as these participants wore a sham (non-functioning) monitor."

5-xii) Describe any co-interventions (incl. training/support)

Describe any co-interventions (incl. training/support): Clearly state any interventions that are provided in addition to the targeted eHealth intervention, as ehealth intervention may not be designed as stand-alone intervention. This includes training sessions and support [1]. It may be necessary to distinguish between the level of training required for the trial, and the level of training for a routine application outside of a RCT setting (discuss under item 21 – generalizability.

	1	2	3	4	5	
subitem not at all important	○	○	○	○	◉	essential

Does your paper address subitem 5-xii? *

Copy and paste relevant sections from the manuscript (include quotes in quotation marks "like this" to indicate direct quotes from your manuscript), or elaborate on this item by providing additional information not in the ms, or briefly explain why the item is not applicable/relevant for your study

None

6a) Completely defined pre-specified primary and secondary outcome measures, including how and when they were assessed

Does your paper address CONSORT subitem 6a? *

Copy and paste relevant sections from the manuscript (include quotes in quotation marks "like this" to indicate direct quotes from your manuscript), or elaborate on this item by providing additional information not in the ms, or briefly explain why the item is not applicable/relevant for your study

PROSPERO ISRCTN17545949.
"Primary outcome: Participant engagement with the Fitbit and Freestyle Libre were assessed by time spent on the associated applications using Ethica Data (Kitchener, Ontario, Canada). Engagement was also assessed by the frequency with which participants scanned the Freestyle Libre, the frequency with which the Fitbit was synced and the number (and type) of changes to the physical activity goals. Before participants left the appointment, verbal and written information was provided about how to apply, activate and scan the Freestyle Libre and how to sync and charge the Fitbit. Default Fitbit physical activity goals were 10,000 steps, 30 active minutes, 10 flights of stairs, 2500 calories and 8 kilometres per day. To record changes to these goals, the researchers checked the participant's study-specific Fitbit accounts daily via the web-based Fitbit platform." "Secondary outcomes: The indicators used to assess feasibility included the number of individuals who accessed and completed the online survey, the number of individuals deemed eligible, uptake and retention, the number of Freestyle Libre sensors provided to participants and non-usage attrition.[20] Notes were made to identify the number of additional sensors provided. For acceptability, the indicators used were Fitbit wear time (defined as the presence of a heart rate signal and not categorised as sleep by Fitbit's proprietary algorithm), the number of times the research team prompted participants to sync or charge the Fitbit, the number of minutes of missing data and the proportion of expected data for the Freestyle Libre."

6a-i) Online questionnaires: describe if they were validated for online use and apply CHERRIES items to describe how the questionnaires were designed/deployed

If outcomes were obtained through online questionnaires, describe if they were validated for online use and apply CHERRIES items to describe how the questionnaires were designed/deployed [9].

	1	2	3	4	5	
subitem not at all important	○	○	◉	○	○	essential

Does your paper address subitem 6a-i?

Copy and paste relevant sections from manuscript text

"Interested individuals were directed to complete The Leicester Risk Assessment[15] to determine level of risk for type 2 diabetes via an online survey (Qualtrics, Provo, Utah, USA)"
This online questionnaire was custom developed for the purposes of recruitment to the trial but the questions have been used elsewhere (by Diabetes UK) as a population level resource to identify level of risk for developing type 2 diabetes. The full list of questions has been provided in multi-media Appendix 1 for reference.

6a-ii) Describe whether and how “use” (including intensity of use/dosage) was defined/measured/monitored

Describe whether and how “use” (including intensity of use/dosage) was defined/measured/monitored (logins, logfile analysis, etc.). Use/adoption metrics are important process outcomes that should be reported in any ehealth trial.

	1	2	3	4	5	
subitem not at all important	○	○	○	○	◉	essential

Does your paper address subitem 6a-ii?

Copy and paste relevant sections from manuscript text

"Fitbit monitors were initialised using the Fitbit application and minute-level data were downloaded via Fitabase (Small Steps Labs LLC., California, USA) and processed using Kinesoft version 3.3.80 (Kinesoft, Loughborough, UK)." "Glucose levels were captured by the LibreLink app and extracted in 15-minute epochs using Diasend (Diasend Inc., Illinois, USA). Interstitial glucose levels were categorised as below range (<4.0 mmol/L), normal (4.0-5.9 mmol/L) or above range (>5.9 mmol/L).[16]" "Participants were notified in the event battery level reached <25% or if ≥5 days had passed since a previous sync (both remotely monitored by the researchers using Fitabase)." "For the Freestyle Libre, participants were asked to scan the sensor once every 7-8 hours to minimise data loss but were not reminded by researchers if they failed to adhere to this." "Participant engagement with the Fitbit and Freestyle Libre were assessed by time spent on the associated applications using Ethica Data (Kitchener, Ontario, Canada). Engagement was also assessed by the frequency with which participants scanned the Freestyle Libre, the frequency with which the Fitbit was synced and the number (and type) of changes to the physical activity goals." "To record changes to these goals, the researchers checked the participant's study-specific Fitbit accounts daily via the web-based Fitbit platform" "the number of individuals who accessed and completed the online survey, the number of individuals deemed eligible, uptake and retention, the number of Freestyle Libre sensors provided to participants and non-usage attrition.[20] Notes were made to identify the number of additional sensors provided." "Fitbit wear time (defined as the presence of a heart rate signal and not categorised as sleep by Fitbit's proprietary algorithm), the number of times the research team prompted participants to sync or charge the Fitbit, the number of minutes of missing data and the proportion of expected data for the Freestyle Libre."

6a-iii) Describe whether, how, and when qualitative feedback from participants was obtained

Describe whether, how, and when qualitative feedback from participants was obtained (e.g., through emails, feedback forms, interviews, focus groups).

	1	2	3	4	5	
subitem not at all important	○	○	○	◉	○	essential

Does your paper address subitem 6a-iii?

Copy and paste relevant sections from manuscript text

"Interviews were completed at the six-week follow up appointment by a member of the research team independent from the quantitative data collection procedures and intervention delivery."

6b) Any changes to trial outcomes after the trial commenced, with reasons

Does your paper address CONSORT subitem 6b? *

Copy and paste relevant sections from the manuscript (include quotes in quotation marks "like this" to indicate direct quotes from your manuscript), or elaborate on this item by providing additional information not in the ms, or briefly explain why the item is not applicable/relevant for your study

There were no changes to trial outcomes after the trial commenced.

7a) How sample size was determined

NPT: When applicable, details of whether and how the clustering by care provides or centers was addressed

7a-i) Describe whether and how expected attrition was taken into account when calculating the sample size

Describe whether and how expected attrition was taken into account when calculating the sample size.

	1	2	3	4	5	
subitem not at all important	○	○	○	◉	○	essential

Does your paper address subitem 7a-i?

Copy and paste relevant sections from manuscript title (include quotes in quotation marks "like this" to indicate direct quotes from your manuscript), or elaborate on this item by providing additional information not in the ms, or briefly explain why the item is not applicable/relevant for your study

Due to the feasibility nature of the trial, no sample size calculation was conducted.

7b) When applicable, explanation of any interim analyses and stopping guidelines

Does your paper address CONSORT subitem 7b? *

Copy and paste relevant sections from the manuscript (include quotes in quotation marks "like this" to indicate direct quotes from your manuscript), or elaborate on this item by providing additional information not in the ms, or briefly explain why the item is not applicable/relevant for your study

No interim analyses were conducted. Recruitment finished upon recruiting the 45th participant.

8a) Method used to generate the random allocation sequence

NPT: When applicable, how care providers were allocated to each trial group

Does your paper address CONSORT subitem 8a? *

Copy and paste relevant sections from the manuscript (include quotes in quotation marks "like this" to indicate direct quotes from your manuscript), or elaborate on this item by providing additional information not in the ms, or briefly explain why the item is not applicable/relevant for your study

"The full protocol has been published[14]" "An independent researcher produced a computer-generated randomisation list with 1:1:1 allocation"

8b) Type of randomisation; details of any restriction (such as blocking and block size)

Does your paper address CONSORT subitem 8b? *

Copy and paste relevant sections from the manuscript (include quotes in quotation marks "like this" to indicate direct quotes from your manuscript), or elaborate on this item by providing additional information not in the ms, or briefly explain why the item is not applicable/relevant for your study

"The full protocol has been published[14]"

9) Mechanism used to implement the random allocation sequence (such as sequentially numbered containers), describing any steps taken to conceal the sequence until interventions were assigned

Does your paper address CONSORT subitem 9? *

Copy and paste relevant sections from the manuscript (include quotes in quotation marks "like this" to indicate direct quotes from your manuscript), or elaborate on this item by providing additional information not in the ms, or briefly explain why the item is not applicable/relevant for your study

"The researcher was informed of the participant's allocation on the day of the appointment to ensure adequate preparation (study paperwork and equipment needs varied between treatment allocations)"

10) Who generated the random allocation sequence, who enrolled participants, and who assigned participants to interventions

Does your paper address CONSORT subitem 10? *

Copy and paste relevant sections from the manuscript (include quotes in quotation marks "like this" to indicate direct quotes from your manuscript), or elaborate on this item by providing additional information not in the ms, or briefly explain why the item is not applicable/relevant for your study

"An independent researcher produced a computer-generated randomisation list" "after the researcher confirmed elgibility, group allocation was revealed to the participant" "the researcher was informed of the participant's allocation on the day of the appointment"

11a) If done, who was blinded after assignment to interventions (for example, participants, care providers, those assessing outcomes) and how

NPT: Whether or not administering co-interventions were blinded to group assignment

11a-i) Specify who was blinded, and who wasn't

Specify who was blinded, and who wasn't. Usually, in web-based trials it is not possible to blind the participants [1, 3] (this should be clearly acknowledged), but it may be possible to blind outcome assessors, those doing data analysis or those administering co-interventions (if any).

	1	2	3	4	5	
subitem not at all important	○	○	○	○	◉	essential

Does your paper address subitem 11a-i? *

Copy and paste relevant sections from the manuscript (include quotes in quotation marks "like this" to indicate direct quotes from your manuscript), or elaborate on this item by providing additional information not in the ms, or briefly explain why the item is not applicable/relevant for your study

"The researcher was informed of the participant's allocation on the day of the appointment to ensure adequate preparation (study paperwork and equipment needs varied between treatment allocations); meaning it was not possible to blind the researcher or participant to treatment allocation."

11a-ii) Discuss e.g., whether participants knew which intervention was the "intervention of interest" and which one was the "comparator"

Informed consent procedures (4a-ii) can create biases and certain expectations - discuss e.g., whether participants knew which intervention was the "intervention of interest" and which one was the "comparator".

	1	2	3	4	5	
subitem not at all important	○	○	○	●	○	essential

Does your paper address subitem 11a-ii?

Copy and paste relevant sections from the manuscript (include quotes in quotation marks "like this" to indicate direct quotes from your manuscript), or elaborate on this item by providing additional information not in the ms, or briefly explain why the item is not applicable/relevant for your study

"After the researcher confirmed eligibility, group allocation was revealed to the participant"
A Participant Information Sheet was provided to participants which described each of the three groups as part of the trial.

11b) If relevant, description of the similarity of interventions

(this item is usually not relevant for ehealth trials as it refers to similarity of a placebo or sham intervention to a active medication/intervention)

Does your paper address CONSORT subitem 11b? *

Copy and paste relevant sections from the manuscript (include quotes in quotation marks "like this" to indicate direct quotes from your manuscript), or elaborate on this item by providing additional information not in the ms, or briefly explain why the item is not applicable/relevant for your study

Not applicable

12a) Statistical methods used to compare groups for primary and secondary outcomes

NPT: When applicable, details of whether and how the clustering by care providers or centers was addressed

Does your paper address CONSORT subitem 12a? *

Copy and paste relevant sections from the manuscript (include quotes in quotation marks "like this" to indicate direct quotes from your manuscript), or elaborate on this item by providing additional information not in the ms, or briefly explain why the item is not applicable/relevant for your study

"Descriptive statistics were reported as mean (standard deviation) or frequency (%) using Statistical Package for Social Sciences version 24.0 (SPSS Inc., Chicago, IL)."

12a-i) Imputation techniques to deal with attrition / missing values

Imputation techniques to deal with attrition / missing values: Not all participants will use the intervention/comparator as intended and attrition is typically high in ehealth trials. Specify how participants who did not use the application or dropped out from the trial were treated in the statistical analysis (a complete case analysis is strongly discouraged, and simple imputation techniques such as LOCF may also be problematic [4]).

	1	2	3	4	5	
subitem not at all important	○	○	○	◉	○	essential

Does your paper address subitem 12a-i? *

Copy and paste relevant sections from the manuscript (include quotes in quotation marks "like this" to indicate direct quotes from your manuscript), or elaborate on this item by providing additional information not in the ms, or briefly explain why the item is not applicable/relevant for your study

No imputation techniques were used. Amount of missing data reported as part of the feasibility assessment.

12b) Methods for additional analyses, such as subgroup analyses and adjusted analyses

Does your paper address CONSORT subitem 12b? *

Copy and paste relevant sections from the manuscript (include quotes in quotation marks "like this" to indicate direct quotes from your manuscript), or elaborate on this item by providing additional information not in the ms, or briefly explain why the item is not applicable/relevant for your study

Descriptive group comparisons were made (using mean, standard deviation or frequencies).

X26) REB/IRB Approval and Ethical Considerations [recommended as subheading under "Methods"] (not a CONSORT item)

X26-i) Comment on ethics committee approval

	1	2	3	4	5	
subitem not at all important	○	○	○	◉	○	essential

Does your paper address subitem X26-i?

Copy and paste relevant sections from the manuscript (include quotes in quotation marks "like this" to indicate direct quotes from your manuscript), or elaborate on this item by providing additional information not in the ms, or briefly explain why the item is not applicable/relevant for your study

"Loughborough University Ethics Advisory Committee provided ethical approval for the study."

x26-ii) Outline informed consent procedures

Outline informed consent procedures e.g., if consent was obtained offline or online (how? Checkbox, etc.?), and what information was provided (see 4a-ii). See [6] for some items to be included in informed consent documents.

	1	2	3	4	5	
subitem not at all important	○	○	○	◉	○	essential

Does your paper address subitem X26-ii?

Copy and paste relevant sections from the manuscript (include quotes in quotation marks "like this" to indicate direct quotes from your manuscript), or elaborate on this item by providing additional information not in the ms, or briefly explain why the item is not applicable/relevant for your study

"All participants provided written informed consent."

X26-iii) Safety and security procedures

Safety and security procedures, incl. privacy considerations, and any steps taken to reduce the likelihood or detection of harm (e.g., education and training, availability of a hotline)

	1	2	3	4	5	
subitem not at all important	○	○	○	◉	○	essential

Does your paper address subitem X26-iii?

Copy and paste relevant sections from the manuscript (include quotes in quotation marks "like this" to indicate direct quotes from your manuscript), or elaborate on this item by providing additional information not in the ms, or briefly explain why the item is not applicable/relevant for your study

Participants had access to a study telephone number in the event any issues or concerns arose. This was detailed in the Participant Information Sheet.

RESULTS

13a) For each group, the numbers of participants who were randomly assigned, received intended treatment, and were analysed for the primary outcome

NPT: The number of care providers or centers performing the intervention in each group and the number of patients treated by each care provider in each center

Does your paper address CONSORT subitem 13a? *

Copy and paste relevant sections from the manuscript (include quotes in quotation marks "like this" to indicate direct quotes from your manuscript), or elaborate on this item by providing additional information not in the ms, or briefly explain why the item is not applicable/relevant for your study

"In total, 525 people visited the online survey, 340 (64.8%) completed the survey and 58 individuals (17.1%; 11% of those visiting the survey) were eligible for the study. Forty-five individuals (77.6% of those eligible) consented to take part and no participants withdrew from the study (Figure 2)."

13b) For each group, losses and exclusions after randomisation, together with reasons

Does your paper address CONSORT subitem 13b? (NOTE: Preferably, this is shown in a CONSORT flow diagram) *

Copy and paste relevant sections from the manuscript (include quotes in quotation marks "like this" to indicate direct quotes from your manuscript), or elaborate on this item by providing additional information not in the ms, or briefly explain why the item is not applicable/relevant for your study

Reasons for excluded outlined in Figure 2 during recruitment but no participants withdrew during the trial itself.

13b-i) Attrition diagram

Strongly recommended: An attrition diagram (e.g., proportion of participants still logging in or using the intervention/comparator in each group plotted over time, similar to a survival curve) or other figures or tables demonstrating usage/dose/engagement.

	1	2	3	4	5	
subitem not at all important	○	○	○	○	◉	essential

Does your paper address subitem 13b-i?

Copy and paste relevant sections from the manuscript or cite the figure number if applicable (include quotes in quotation marks "like this" to indicate direct quotes from your manuscript), or elaborate on this item by providing additional information not in the ms, or briefly explain why the item is not applicable/relevant for your study

"From weeks 1 to 6, the groups G4GPA2 and GPA6 spent a lower amount of time on the Freestyle Libre application, going from 28.3 to 12.3 min/day and 11.5 to 5.5 min/day, respectively (Figure 3; Panel A). PA4GPA2 participants logged 7.1 min/day in week 5 and 4.7 min/day in week 6. A similar pattern was observed for the Freestyle Libre application whereby participants in groups PA4GPA2 and GPA6 observed a reduction in time spent on the Fitbit application, reducing from 6.7 to 3.4 min/day and 7.6 to 3.9 min/day, respectively (Figure 3; Panel B). Similarly, participants in G4GPA2 reduced their application usage from weeks 5 to 6 from 16.9 to 12.7 min/day (the only weeks when they could access it)." Visual depicts this in Figure 3.
"The average number of scans declined over time across all three groups (Figure 4). In the groups G4GPA2 and GPA6, participants logged on average 9.4 scans/day in week 1 and 6.8 scans/day in week 6. Across weeks 5 and 6, participants in PA4GPA2 conducted 6.3 scans/day and 5.6 scans/day, respectively. A number of Fitbit monitors unexpectedly restored to default settings during deployment which resulted in syncs being completed automatically." Visual depicts this in Figure 4.

14a) Dates defining the periods of recruitment and follow-up

Does your paper address CONSORT subitem 14a? *

Copy and paste relevant sections from the manuscript (include quotes in quotation marks "like this" to indicate direct quotes from your manuscript), or elaborate on this item by providing additional information not in the ms, or briefly explain why the item is not applicable/relevant for your study

"Participants were recruited between May and September 2017"

14a-i) Indicate if critical "secular events" fell into the study period

Indicate if critical "secular events" fell into the study period, e.g., significant changes in Internet resources available or "changes in computer hardware or Internet delivery resources"

	1	2	3	4	5	
subitem not at all important	○	○	○	◉	○	essential

Does your paper address subitem 14a-i?

Copy and paste relevant sections from the manuscript (include quotes in quotation marks "like this" to indicate direct quotes from your manuscript), or elaborate on this item by providing additional information not in the ms, or briefly explain why the item is not applicable/relevant for your study

No critical secular events took place.

14b) Why the trial ended or was stopped (early)

Does your paper address CONSORT subitem 14b? *

Copy and paste relevant sections from the manuscript (include quotes in quotation marks "like this" to indicate direct quotes from your manuscript), or elaborate on this item by providing additional information not in the ms, or briefly explain why the item is not applicable/relevant for your study

The trial was not stopped early.

15) A table showing baseline demographic and clinical characteristics for each group

NPT: When applicable, a description of care providers (case volume, qualification, expertise, etc.) and centers (volume) in each group

Does your paper address CONSORT subitem 15? *

Copy and paste relevant sections from the manuscript (include quotes in quotation marks "like this" to indicate direct quotes from your manuscript), or elaborate on this item by providing additional information not in the ms, or briefly explain why the item is not applicable/relevant for your study

"The sample was made up of more females (60%), had a mean±SD age of 56±8.7 years and were predominantly White British (88.9%) (Table 1). Seven participants (15.6%) were identified as being at high-risk of developing type 2 diabetes, 3 (6.7%) were classified as living with prediabetes, 17 (37.8%) were overweight and 23 (51.1%) had obesity. Forty participants (88.9%) did not comply with the UK physical activity guidelines at baseline (Table 2)."

15-i) Report demographics associated with digital divide issues

In ehealth trials it is particularly important to report demographics associated with digital divide issues, such as age, education, gender, social-economic status, computer/Internet/ehealth literacy of the participants, if known.

	1	2	3	4	5	
subitem not at all important	○	○	○	●	○	essential

Does your paper address subitem 15-i? *

Copy and paste relevant sections from the manuscript (include quotes in quotation marks "like this" to indicate direct quotes from your manuscript), or elaborate on this item by providing additional information not in the ms, or briefly explain why the item is not applicable/relevant for your study

Refer to Table 1.

16) For each group, number of participants (denominator) included in each analysis and whether the analysis was by original assigned groups

16-i) Report multiple “denominators” and provide definitions

Report multiple “denominators” and provide definitions: Report N’s (and effect sizes) “across a range of study participation [and use] thresholds” [1], e.g., N exposed, N consented, N used more than x times, N used more than y weeks, N participants “used” the intervention/comparator at specific pre-defined time points of interest (in absolute and relative numbers per group). Always clearly define “use” of the intervention.

	1	2	3	4	5	
subitem not at all important	○	○	○	●	○	essential

Does your paper address subitem 16-i? *

Copy and paste relevant sections from the manuscript (include quotes in quotation marks "like this" to indicate direct quotes from your manuscript), or elaborate on this item by providing additional information not in the ms, or briefly explain why the item is not applicable/relevant for your study

"Twenty-two participants (48.9%) completed the study using the minimum number of three Freestyle Libre sensors. Eleven of the 23 participants requiring extra sensors (47.8%) had one faulty or misplaced sensor, 8 (34.8%) had two, 2 (8.7%) had three and the remaining 2 (8.7%) participants had four faulty or displaced sensors. It was noted that 27 participants (60%) set the LibreLink application to remind them to scan the glucose sensor. There were no instances of non-usage attrition." "During the six weeks, 22 of 45 participants (48.9%) provided 42 days of valid Fitbit wear with all participants averaging a total of 40.1±3.2 valid days. Compliance with syncing the Fitbit data noted that 12 participants (26.7%) received a prompt from the researchers to sync (encourage data transfer) whilst 5 (11.1%), 3 (6.7%), 2 (4.4%) and 2 (4.4%) participants received 2, 3, 4 or 5 prompts, respectively. In terms of charging the Fitbit, 9 (20%) received a prompt to charge the Fitbit as battery status reached <25%. No data losses were recorded for the Fitbit. The level of data capture for the Freestyle Libre was high (an average of 87.6% (±3.8) and 82% (±19) in the first and sixth week, respectively (Appendix 4)." "From weeks 1 to 6, the groups G4GPA2 and GPA6 spent a lower amount of time on the Freestyle Libre application, going from 28.3 to 12.3 min/day and 11.5 to 5.5 min/day, respectively (Figure 3; Panel A). PA4GPA2 participants logged 7.1 min/day in week 5 and 4.7 min/day in week 6. A similar pattern was observed for the Freestyle Libre application whereby participants in groups PA4GPA2 and GPA6 observed a reduction in time spent on the Fitbit application, reducing from 6.7 to 3.4 min/day and 7.6 to 3.9 min/day, respectively (Figure 3; Panel B). Similarly, participants in G4GPA2 reduced their application usage from weeks 5 to 6 from 16.9 to 12.7 min/day (the only weeks when they could access it)." "The average number of scans declined over time across all three groups (Figure 4). In the groups G4GPA2 and GPA6, participants logged on average 9.4 scans/day in week 1 and 6.8 scans/day in week 6. Across weeks 5 and 6, participants in PA4GPA2 conducted 6.3 scans/day and 5.6 scans/day, respectively. A number of Fitbit monitors unexpectedly restored to default settings during deployment which resulted in syncs being completed automatically. " "Thirteen of 45 participants (28.9%) changed ≥1 of the physical activity goals from the default settings. Of these participants, 9 (69.2%) changed the daily step goal whilst the number of floors, active minutes, calories and distance goals were changed by 5 (38.5%), 3 (23.1%), 2 (15.4%) and 2 (15.4%) participants, respectively. Notably, the daily step goal was reduced by 7 participants (77.8%)."

16-ii) Primary analysis should be intent-to-treat

Primary analysis should be intent-to-treat, secondary analyses could include comparing only “users”, with the appropriate caveats that this is no longer a randomized sample (see 18-i).

	1	2	3	4	5	
subitem not at all important	○	○	◉	○	○	essential

Does your paper address subitem 16-ii?

Copy and paste relevant sections from the manuscript (include quotes in quotation marks "like this" to indicate direct quotes from your manuscript), or elaborate on this item by providing additional information not in the ms, or briefly explain why the item is not applicable/relevant for your study

Intention-to-treat analysis was not deemed suitable for the feasibility nature of the trial.

17a) For each primary and secondary outcome, results for each group, and the estimated effect size and its precision (such as 95% confidence interval)

Does your paper address CONSORT subitem 17a? *

Copy and paste relevant sections from the manuscript (include quotes in quotation marks "like this" to indicate direct quotes from your manuscript), or elaborate on this item by providing additional information not in the ms, or briefly explain why the item is not applicable/relevant for your study

No formal statistical analyses were conducted due to feasibility nature of the trial.

17a-i) Presentation of process outcomes such as metrics of use and intensity of use

In addition to primary/secondary (clinical) outcomes, the presentation of process outcomes such as metrics of use and intensity of use (dose, exposure) and their operational definitions is critical. This does not only refer to metrics of attrition (13-b) (often a binary variable), but also to more continuous exposure metrics such as “average session length”. These must be accompanied by a technical description how a metric like a “session” is defined (e.g., timeout after idle time) [1] (report under item 6a).

	1	2	3	4	5	
subitem not at all important	○	○	○	◉	○	essential

Does your paper address subitem 17a-i?

Copy and paste relevant sections from the manuscript (include quotes in quotation marks "like this" to indicate direct quotes from your manuscript), or elaborate on this item by providing additional information not in the ms, or briefly explain why the item is not applicable/relevant for your study

"Twenty-two participants (48.9%) completed the study using the minimum number of three Freestyle Libre sensors. Eleven of the 23 participants requiring extra sensors (47.8%) had one faulty or misplaced sensor, 8 (34.8%) had two, 2 (8.7%) had three and the remaining 2 (8.7%) participants had four faulty or displaced sensors. It was noted that 27 participants (60%) set the LibreLink application to remind them to scan the glucose sensor. There were no instances of non-usage attrition." "During the six weeks, 22 of 45 participants (48.9%) provided 42 days of valid Fitbit wear with all participants averaging a total of 40.1±3.2 valid days. Compliance with syncing the Fitbit data noted that 12 participants (26.7%) received a prompt from the researchers to sync (encourage data transfer) whilst 5 (11.1%), 3 (6.7%), 2 (4.4%) and 2 (4.4%) participants received 2, 3, 4 or 5 prompts, respectively. In terms of charging the Fitbit, 9 (20%) received a prompt to charge the Fitbit as battery status reached <25%. No data losses were recorded for the Fitbit. The level of data capture for the Freestyle Libre was high (an average of 87.6% (±3.8) and 82% (±19) in the first and sixth week, respectively (Appendix 4)." "From weeks 1 to 6, the groups G4GPA2 and GPA6 spent a lower amount of time on the Freestyle Libre application, going from 28.3 to 12.3 min/day and 11.5 to 5.5 min/day, respectively (Figure 3; Panel A). PA4GPA2 participants logged 7.1 min/day in week 5 and 4.7 min/day in week 6. A similar pattern was observed for the Freestyle Libre application whereby participants in groups PA4GPA2 and GPA6 observed a reduction in time spent on the Fitbit application, reducing from 6.7 to 3.4 min/day and 7.6 to 3.9 min/day, respectively (Figure 3; Panel B). Similarly, participants in G4GPA2 reduced their application usage from weeks 5 to 6 from 16.9 to 12.7 min/day (the only weeks when they could access it)." "The average number of scans declined over time across all three groups (Figure 4). In the groups G4GPA2 and GPA6, participants logged on average 9.4 scans/day in week 1 and 6.8 scans/day in week 6. Across weeks 5 and 6, participants in PA4GPA2 conducted 6.3 scans/day and 5.6 scans/day, respectively. A number of Fitbit monitors unexpectedly restored to default settings during deployment which resulted in syncs being completed automatically. " "Thirteen of 45 participants (28.9%) changed ≥1 of the physical activity goals from the default settings. Of these participants, 9 (69.2%) changed the daily step goal whilst the number of floors, active minutes, calories and distance goals were changed by 5 (38.5%), 3 (23.1%), 2 (15.4%) and 2 (15.4%) participants, respectively. Notably, the daily step goal was reduced by 7 participants (77.8%)."

17b) For binary outcomes, presentation of both absolute and relative effect sizes is recommended

Does your paper address CONSORT subitem 17b? *

Copy and paste relevant sections from the manuscript (include quotes in quotation marks "like this" to indicate direct quotes from your manuscript), or elaborate on this item by providing additional information not in the ms, or briefly explain why the item is not applicable/relevant for your study

Not applicable due to the feasibility nature of the trial.

18) Results of any other analyses performed, including subgroup analyses and adjusted analyses, distinguishing pre-specified from exploratory

Does your paper address CONSORT subitem 18? *

Copy and paste relevant sections from the manuscript (include quotes in quotation marks "like this" to indicate direct quotes from your manuscript), or elaborate on this item by providing additional information not in the ms, or briefly explain why the item is not applicable/relevant for your study

Not applicable due to the feasibility nature of the trial.

18-i) Subgroup analysis of comparing only users

A subgroup analysis of comparing only users is not uncommon in ehealth trials, but if done, it must be stressed that this is a self-selected sample and no longer an unbiased sample from a randomized trial (see 16-iii).

	1	2	3	4	5	
subitem not at all important	○	○	○	◉	○	essential

Does your paper address subitem 18-i?

Copy and paste relevant sections from the manuscript (include quotes in quotation marks "like this" to indicate direct quotes from your manuscript), or elaborate on this item by providing additional information not in the ms, or briefly explain why the item is not applicable/relevant for your study

All (descriptive) analyses were conducted at the original group level.

19) All important harms or unintended effects in each group

(for specific guidance see CONSORT for harms)

Does your paper address CONSORT subitem 19? *

Copy and paste relevant sections from the manuscript (include quotes in quotation marks "like this" to indicate direct quotes from your manuscript), or elaborate on this item by providing additional information not in the ms, or briefly explain why the item is not applicable/relevant for your study

"Twenty-two participants (48.9%) completed the study using the minimum number of three Freestyle Libre sensors. Eleven of the 23 participants requiring extra sensors (47.8%) had one faulty or misplaced sensor, 8 (34.8%) had two, 2 (8.7%) had three and the remaining 2 (8.7%) participants had four faulty or displaced sensors"

19-i) Include privacy breaches, technical problems

Include privacy breaches, technical problems. This does not only include physical "harm" to participants, but also incidents such as perceived or real privacy breaches [1], technical problems, and other unexpected/unintended incidents. "Unintended effects" also includes unintended positive effects [2].

	1	2	3	4	5	
subitem not at all important	○	○	○	○	●	essential

Does your paper address subitem 19-i?

Copy and paste relevant sections from the manuscript (include quotes in quotation marks "like this" to indicate direct quotes from your manuscript), or elaborate on this item by providing additional information not in the ms, or briefly explain why the item is not applicable/relevant for your study

"Comments relating to applying the Freestyle Libre emphasised the initial trepidation felt by participants, mostly due to the visible needle and concern that the insertion process would be painful. However, there was pleasant surprise with which the Freestyle Libre adhered to the skin and this nervousness typically subsided following the first application." "A few indicated issues with skin irritation from the Tegaderm and having trouble applying the sensor correctly." "The requirement of scanning the Freestyle Libre at least every 8 hours to avoid data loss was highlighted as a flaw with the technology, as some participants forgot to scan regularly enough, and several noticed periods of missing data during sleep." "multiple attempts were sometimes needed to scan the Freestyle Libre."

19-ii) Include qualitative feedback from participants or observations from staff/researchers

Include qualitative feedback from participants or observations from staff/researchers, if available, on strengths and shortcomings of the application, especially if they point to unintended/unexpected effects or uses. This includes (if available) reasons for why people did or did not use the application as intended by the developers.

	1	2	3	4	5	
subitem not at all important	○	○	○	○	◉	essential

Does your paper address subitem 19-ii?

Copy and paste relevant sections from the manuscript (include quotes in quotation marks "like this" to indicate direct quotes from your manuscript), or elaborate on this item by providing additional information not in the ms, or briefly explain why the item is not applicable/relevant for your study

Full qualitative feedback is provided in the Results section under the two key qualitative sub-headed sections.

DISCUSSION

22) Interpretation consistent with results, balancing benefits and harms, and considering other relevant evidence

NPT: In addition, take into account the choice of the comparator, lack of or partial blinding, and unequal expertise of care providers or centers in each group

22-i) Restate study questions and summarize the answers suggested by the data, starting with primary outcomes and process outcomes (use)

Restate study questions and summarize the answers suggested by the data, starting with primary outcomes and process outcomes (use).

	1	2	3	4	5	
subitem not at all important	○	○	○	○	◉	essential

Does your paper address subitem 22-i? *

Copy and paste relevant sections from the manuscript (include quotes in quotation marks "like this" to indicate direct quotes from your manuscript), or elaborate on this item by providing additional information not in the ms, or briefly explain why the item is not applicable/relevant for your study

"Engagement with the devices reduced over the six-week intervention period but remained higher than the minimum level of engagement needed to avoid data loss (e.g. scanning the Freestyle Libre every eight hours). Despite limitations with smartphone compatibility, it was feasible to conduct the study, with a high uptake rate and full retention of participants at the end of the trial. The study and technologies were acceptable to individuals at risk of type 2 diabetes, but areas of refinement were highlighted." "Scans per day reduced by ~28% between week 1 and week 6 whereas time spent on the app decreased by 52-57% for both groups accessing the Freestyle Libre for the full six weeks." "Eligibility of individuals completing the online risk survey was low (17%) with 63% of those who were ineligible for the study classified as having a low risk of developing type 2 diabetes and 32% having a non-compatible smartphone." "less than a fifth of the sample were deemed at 'high risk', only 7% were prediabetic and there was significant ethnic homogeneity with almost 90% of the sample White British; despite recruiting from an area with a multi-ethnic population"

22-ii) Highlight unanswered new questions, suggest future research

Highlight unanswered new questions, suggest future research.

	1	2	3	4	5	
subitem not at all important	○	○	○	●	○	essential

Does your paper address subitem 22-ii?

Copy and paste relevant sections from the manuscript (include quotes in quotation marks "like this" to indicate direct quotes from your manuscript), or elaborate on this item by providing additional information not in the ms, or briefly explain why the item is not applicable/relevant for your study

"This study highlights several important areas for future research, notably: (i) the inclusion of a more diverse pool of individuals at risk of developing type 2 diabetes or identified as living with prediabetes and (ii) the detection and presentation of teachable moments linking behavioural choices with acute physiological consequences in controlled and free-living settings. Key areas for industrial and research sectors to collectively consider include: (i) the need to integrate information collected by behavioural and physiological sensors; (ii) to provide intelligent feedback and automated insights by using multiple data sources in real-time; and (iii) improve device aesthetics."

20) Trial limitations, addressing sources of potential bias, imprecision, and, if relevant, multiplicity of analyses

20-i) Typical limitations in ehealth trials

Typical limitations in ehealth trials: Participants in ehealth trials are rarely blinded. Ehealth trials often look at a multiplicity of outcomes, increasing risk for a Type I error. Discuss biases due to non-use of the intervention/usability issues, biases through informed consent procedures, unexpected events.

	1	2	3	4	5	
subitem not at all important	○	○	○	◉	○	essential

Does your paper address subitem 20-i? *

Copy and paste relevant sections from the manuscript (include quotes in quotation marks "like this" to indicate direct quotes from your manuscript), or elaborate on this item by providing additional information not in the ms, or briefly explain why the item is not applicable/relevant for your study

"In addition to the limitations previously disclosed, the study had a relatively small sample size limiting insight from statistical analyses. Assessor and participant blinding to group allocations was not possible. Additionally, it was not possible to quantify what participants specifically looked at within the Fitbit and Freestyle Libre applications. Due to its raison d'être as an intervention tool, it was not possible to set up the Freestyle Libre in a masked mode (as achieved with the Fitbit). Consequently, no glucose data were collected from the PA4GPA2 group for the first 4 weeks of the intervention period."

21) Generalisability (external validity, applicability) of the trial findings

NPT: External validity of the trial findings according to the intervention, comparators, patients, and care providers or centers involved in the trial

21-i) Generalizability to other populations

Generalizability to other populations: In particular, discuss generalizability to a general Internet population, outside of a RCT setting, and general patient population, including applicability of the study results for other organizations

	1	2	3	4	5	
subitem not at all important	○	○	○	●	○	essential

Does your paper address subitem 21-i?

Copy and paste relevant sections from the manuscript (include quotes in quotation marks "like this" to indicate direct quotes from your manuscript), or elaborate on this item by providing additional information not in the ms, or briefly explain why the item is not applicable/relevant for your study

"After experiencing the Freestyle Libre for at least a couple of weeks, many participants saw the glucose monitor as having great potential for those already with type 2 diabetes. Aligning with this narrative is the Freestyle Libre since being made available on prescription by the NHS for people who meet a clear criteria (including currently undertake intensive monitoring >8 times daily and have an impaired awareness of hypoglycaemia) with national coverage from April 2019[33]."

21-ii) Discuss if there were elements in the RCT that would be different in a routine application setting

Discuss if there were elements in the RCT that would be different in a routine application setting (e.g., prompts/reminders, more human involvement, training sessions or other co-interventions) and what impact the omission of these elements could have on use, adoption, or outcomes if the intervention is applied outside of a RCT setting.

	1	2	3	4	5	
subitem not at all important	○	○	○	●	○	essential

Does your paper address subitem 21-ii?

Copy and paste relevant sections from the manuscript (include quotes in quotation marks "like this" to indicate direct quotes from your manuscript), or elaborate on this item by providing additional information not in the ms, or briefly explain why the item is not applicable/relevant for your study

"Participants expressed the need for further instruction and demonstration of the technologies and assistance with interpreting the feedback." "The need for a human element in such prevention approaches, either to provide education, demonstrate devices, prescribe exercise or motivate individuals to make positive lifestyle changes, is something current digital health technologies cannot replicate nor replace"

OTHER INFORMATION

23) Registration number and name of trial registry

Does your paper address CONSORT subitem 23? *

Copy and paste relevant sections from the manuscript (include quotes in quotation marks "like this" to indicate direct quotes from your manuscript), or elaborate on this item by providing additional information not in the ms, or briefly explain why the item is not applicable/relevant for your study

ISRCTN17545949

24) Where the full trial protocol can be accessed, if available

Does your paper address CONSORT subitem 24? *

Cite a Multimedia Appendix, other reference, or copy and paste relevant sections from the manuscript (include quotes in quotation marks "like this" to indicate direct quotes from your manuscript), or elaborate on this item by providing additional information not in the ms, or briefly explain why the item is not applicable/relevant for your study

https://bmjopen.bmj.com/content/7/10/e018282

25) Sources of funding and other support (such as supply of drugs), role of funders

Does your paper address CONSORT subitem 25? *

Copy and paste relevant sections from the manuscript (include quotes in quotation marks "like this" to indicate direct quotes from your manuscript), or elaborate on this item by providing additional information not in the ms, or briefly explain why the item is not applicable/relevant for your study

"This work was funded in part by philanthropic support received from the late Dr the Honourable David Saul. This work has also been supported in part by the Higher Education Institution Challenge for Patient Supported Quality Improvement and Education in Health and Social Care (funded by the East Midlands Academic Health Science Network) for the involvement of members of the public in research and by Loughborough University School of Sport, Exercise and Health Science for research facilitation funds."

X27) Conflicts of Interest (not a CONSORT item)

X27-i) State the relation of the study team towards the system being evaluated

In addition to the usual declaration of interests (financial or otherwise), also state the relation of the study team towards the system being evaluated, i.e., state if the authors/evaluators are distinct from or identical with the developers/sponsors of the intervention.

	1	2	3	4	5	
subitem not at all important	○	○	○	○	◉	essential

Does your paper address subitem X27-i?

Copy and paste relevant sections from the manuscript (include quotes in quotation marks "like this" to indicate direct quotes from your manuscript), or elaborate on this item by providing additional information not in the ms, or briefly explain why the item is not applicable/relevant for your study

The authors declare they have no competing interests.

About the CONSORT EHEALTH checklist

As a result of using this checklist, did you make changes in your manuscript? *

- ( ) yes, major changes
- ( ) yes, minor changes
- (•) no

What were the most important changes you made as a result of using this checklist?

Your answer

How much time did you spend on going through the checklist INCLUDING making changes in your manuscript *

2 hours

As a result of using this checklist, do you think your manuscript has improved? *

- (•) yes
- ( ) no
- ( ) Other:

Would you like to become involved in the CONSORT EHEALTH group?

This would involve for example becoming involved in participating in a workshop and writing an "Explanation and Elaboration" document

- ( ) yes
- (•) no
- ( ) Other:

Any other comments or questions on CONSORT EHEALTH

Your answer

STOP - Save this form as PDF before you click submit

To generate a record that you filled in this form, we recommend to generate a PDF of this page (on a Mac, simply select "print" and then select "print as PDF") before you submit it.

When you submit your (revised) paper to JMIR, please upload the PDF as supplementary file.

Don't worry if some text in the textboxes is cut off, as we still have the complete information in our database. Thank you!

Final step: Click submit !

Click submit so we have your answers in our database!

SUBMIT

Never submit passwords through Google Forms.

This content is neither created nor endorsed by Google. Report Abuse - Terms of Service

Google Forms
